# Global, regional, and national deaths, prevalence, disability-adjusted life years, and years lived with disability for chronic obstructive pulmonary disease and asthma, 1990–2015: a systematic analysis for the Global Burden of Disease Study 2015

**DOI:** 10.1016/S2213-2600(17)30293-X

**Published:** 2017-09

**Authors:** GBD 2015 Chronic Respiratory Disease Collaborators, Joan B Soriano, Amanuel Alemu Abajobir, Kalkidan Hassen Abate, Semaw Ferede Abera, Anurag Agrawal, Muktar Beshir Ahmed, Amani Nidhal Aichour, Ibtihel Aichour, Miloud Taki Eddine Aichour, Khurshid Alam, Noore Alam, Juma M Alkaabi, Fatma Al-Maskari, Nelson Alvis-Guzman, Alemayehu Amberbir, Yaw Ampem Amoako, Mustafa Geleto Ansha, Josep M Antó, Hamid Asayesh, Tesfay Mehari Atey, Euripide Frinel G Arthur Avokpaho, Aleksandra Barac, Sanjay Basu, Neeraj Bedi, Isabela M Bensenor, Adugnaw Berhane, Addisu Shunu Beyene, Zulfiqar A Bhutta, Stan Biryukov, Dube Jara Boneya, Michael Brauer, David O Carpenter, Daniel Casey, Devasahayam Jesudas Christopher, Lalit Dandona, Rakhi Dandona, Samath D Dharmaratne, Huyen Phuc Do, Florian Fischer, Ayele Geleto, Aloke Gopal Ghoshal, Richard F Gillum, Ibrahim Abdelmageem Mohamed Ginawi, Vipin Gupta, Simon I Hay, Mohammad T Hedayati, Nobuyuki Horita, H Dean Hosgood, Mihajlo (Michael) B Jakovljevic, Spencer Lewis James, Jost B Jonas, Amir Kasaeian, Yousef Saleh Khader, Ibrahim A Khalil, Ejaz Ahmad Khan, Young-Ho Khang, Jagdish Khubchandani, Luke D Knibbs, Soewarta Kosen, Parvaiz A Koul, G Anil Kumar, Cheru Tesema Leshargie, Xiaofeng Liang, Hassan Magdy Abd El Razek, Azeem Majeed, Deborah Carvalho Malta, Treh Manhertz, Neal Marquez, Alem Mehari, George A Mensah, Ted R Miller, Karzan Abdulmuhsin Mohammad, Kedir Endris Mohammed, Shafiu Mohammed, Ali H Mokdad, Mohsen Naghavi, Cuong Tat Nguyen, Grant Nguyen, Quyen Le Nguyen, Trang Huyen Nguyen, Dina Nur Anggraini Ningrum, Vuong Minh Nong, Jennifer Ifeoma Obi, Yewande E Odeyemi, Felix Akpojene Ogbo, Eyal Oren, Mahesh PA, Eun-Kee Park, George C Patton, Katherine Paulson, Mostafa Qorbani, Reginald Quansah, Anwar Rafay, Mohammad Hifz Ur Rahman, Rajesh Kumar Rai, Salman Rawaf, Nik Reinig, Saeid Safiri, Rodrigo Sarmiento-Suarez, Benn Sartorius, Miloje Savic, Monika Sawhney, Mika Shigematsu, Mari Smith, Fentaw Tadese, George D Thurston, Roman Topor-Madry, Bach Xuan Tran, Kingsley Nnanna Ukwaja, Job F M van Boven, Vasiliy Victorovich Vlassov, Stein Emil Vollset, Xia Wan, Andrea Werdecker, Sarah Wulf Hanson, Yuichiro Yano, Hassen Hamid Yimam, Naohiro Yonemoto, Chuanhua Yu, Zoubida Zaidi, Maysaa El Sayed Zaki, Christopher J L Murray, Theo Vos

## Abstract

**Background:**

Chronic obstructive pulmonary disease (COPD) and asthma are common diseases with a heterogeneous distribution worldwide. Here, we present methods and disease and risk estimates for COPD and asthma from the Global Burden of Diseases, Injuries, and Risk Factors (GBD) 2015 study. The GBD study provides annual updates on estimates of deaths, prevalence, and disability-adjusted life years (DALYs), a summary measure of fatal and non-fatal disease outcomes, for over 300 diseases and injuries, for 188 countries from 1990 to the most recent year.

**Methods:**

We estimated numbers of deaths due to COPD and asthma using the GBD Cause of Death Ensemble modelling (CODEm) tool. First, we analysed data from vital registration and verbal autopsy for the aggregate category of all chronic respiratory diseases. Subsequently, models were run for asthma and COPD relying on covariates to predict rates in countries that have incomplete or no vital registration data. Disease estimates for COPD and asthma were based on systematic reviews of published papers, unpublished reports, surveys, and health service encounter data from the USA. We used the Global Initiative of Chronic Obstructive Lung Disease spirometry-based definition as the reference for COPD and a reported diagnosis of asthma with current wheeze as the definition of asthma. We used a Bayesian meta-regression tool, DisMod-MR 2.1, to derive estimates of prevalence and incidence. We estimated population-attributable fractions for risk factors for COPD and asthma from exposure data, relative risks, and a theoretical minimum exposure level. Results were stratified by Socio-demographic Index (SDI), a composite measure of income per capita, mean years of education over the age of 15 years, and total fertility rate.

**Findings:**

In 2015, 3·2 million people (95% uncertainty interval [UI] 3·1 million to 3·3 million) died from COPD worldwide, an increase of 11·6% (95% UI 5·3 to 19·8) compared with 1990. There was a decrease in age-standardised death rate of 41·9% (37·7 to 45·1) but this was counteracted by population growth and ageing of the global population. From 1990 to 2015, the prevalence of COPD increased by 44·2% (41·7 to 46·6), whereas age-standardised prevalence decreased by 14·7% (13·5 to 15·9). In 2015, 0·40 million people (0·36 million to 0·44 million) died from asthma, a decrease of 26·7% (−7·2 to 43·7) from 1990, and the age-standardised death rate decreased by 58·8% (39·0 to 69·0). The prevalence of asthma increased by 12·6% (9·0 to 16·4), whereas the age-standardised prevalence decreased by 17·7% (15·1 to 19·9). Age-standardised DALY rates due to COPD increased until the middle range of the SDI before reducing sharply. Age-standardised DALY rates due to asthma in both sexes decreased monotonically with rising SDI. The relation between with SDI and DALY rates due to asthma was attributed to variation in years of life lost (YLLs), whereas DALY rates due to COPD varied similarly for YLLs and years lived with disability across the SDI continuum. Smoking and ambient particulate matter were the main risk factors for COPD followed by household air pollution, occupational particulates, ozone, and secondhand smoke. Together, these risks explained 73·3% (95% UI 65·8 to 80·1) of DALYs due to COPD. Smoking and occupational asthmagens were the only risks quantified for asthma in GBD, accounting for 16·5% (14·6 to 18·7) of DALYs due to asthma.

**Interpretation:**

Asthma was the most prevalent chronic respiratory disease worldwide in 2015, with twice the number of cases of COPD. Deaths from COPD were eight times more common than deaths from asthma. In 2015, COPD caused 2·6% of global DALYs and asthma 1·1% of global DALYs. Although there are laudable international collaborative efforts to make surveys of asthma and COPD more comparable, no consensus exists on case definitions and how to measure disease severity for population health measurements like GBD. Comparisons between countries and over time are important, as much of the chronic respiratory burden is either preventable or treatable with affordable interventions.

**Funding:**

Bill & Melinda Gates Foundation.

**Research in context**

**Evidence before this study**

Chronic obstructive pulmonary disease (COPD) and asthma have been identified as important contributors to fatal and non-fatal disease burden in all iterations of the Global Burden of Disease study (GBD). Since the 1990s, two landmark epidemiological studies in asthma, the International Study of Asthma and Allergies in Childhood and the European Community Respiratory Health Survey, provided comparable evidence of the asthma prevalence in children and adults, respectively, but in a limited number of countries. Similarly, for COPD, international initiatives such as PREPOCOL, PLATINO, BOLD, IBERPOC, and EPI-SCAN used standardised population spirometry to quantify COPD and its severity. An absence of consensus on case definitions and other sources of measurement bias between data sources complicates their estimations.

**Added value of this study**

In this study, we provide details on the methods used in GBD to minimise measurement error introduced by heterogeneous cause of death and prevalence data on COPD and asthma. We also provide an analysis of how sociodemographic development has a different effect on the burden of COPD and asthma. We show that mortality but not prevalence of asthma is strongly related to sociodemographic development. For COPD, the burden increases from low sociodemographic development to the mid-range of our Socio-demographic Index and decreases with increasing development, most likely through the pathways of exposure to smoking and environmental risks. We also present risk factor estimates and discuss potential new risks that can be added in future GBD iterations.

**Implications of all the available evidence**

COPD and asthma are important contributors to the burden of non-communicable diseases. Although much of the burden is either preventable or treatable with affordable interventions, these diseases have received less attention than other non-communicable diseases. Up-to-date population information on these diseases is key to policy making to improve access to and quality of existing intervention strategies.

## Introduction

Chronic respiratory diseases are among the leading causes of mortality and morbidity worldwide. Of all chronic respiratory diseases, chronic obstructive pulmonary disease (COPD) and asthma are the most common. These diseases ranked among the top 20 conditions causing disability globally and were ranked eighth (COPD) and 23rd (asthma) as causes of disease burden as measured by disability-adjusted life years (DALYs) in 2015.^1^, ^2^ Yet the measurement of mortality, prevalence, and other population indicators of these two diseases is complicated by misclassification and an absence of consensus about case definitions. Both death rates and prevalence of COPD steeply increase with age. The age pattern of asthma mortality resembles that of COPD rather than the relatively steady prevalence in adults seen in asthma surveys and health service encounter data. This difference in age patterns between cause of death and prevalence data sources has been attributed to a range of factors including the commonly reported misclassification of asthma in the elderly as COPD, variable and temporal effects of smoking, and an actual overlap of asthma and COPD (asthma COPD overlap; ACO).^3^, ^4^ However, no consensus exists on the definition of ACO to date.^5^ Also, evidence from a longitudinal study^6^ did not show a larger reduction in lung function in those patients with COPD and asthma than those without asthma, whereas others have challenged the concept of ACO altogether.^7^, ^8^

Spirometry is the fundamental tool used to define and stage COPD and, accordingly, establish population prevalence in surveys. Under the umbrella of the burden of obstructive lung disease (BOLD) initiative, surveys have been done in 29 countries, with surveys in a further nine countries still in progress.^9^ Two previous initiatives in five Colombian cities (PREPOCOL)^10^ and in five Latin American capital cities (PLATINO)^11^ provided more population estimates. Although all these studies used comparable methods, there is still no universal consensus about the thresholds of spirometry findings to define COPD.^12^, ^13^ The two dominant case definitions for airflow limitation compatible with COPD are a value of less than 0·70 for the ratio of FEV_1_ and forced vital capacity (FVC), or the lower limit of normal (LLN) method of deriving a threshold as the fifth percentile of FEV_1_:FVC in a healthy reference population.^14^ No universal LLN threshold exists because it is thought to vary between populations.^15^ Because most people identified with COPD based on spirometry findings report not having been diagnosed prior to survey, population screening and case-finding in symptomatic smokers have been suggested to provide an opportunity for smoking cessation interventions before the disease has progressed.^16^, ^17^

Most surveys of asthma use a case definition based on self-report of a diagnosis of asthma by a physician and wheeze (with other respiratory symptoms) in the past 12 months.^18^ Others have suggested that wheezing symptoms in the past year and bronchial hyperresponsiveness to inhalation of methacholine or histamine that is reversible with a bronchodilator is a better case definition for clinically relevant asthma.^19^ This case definition has been used to measure asthma prevalence in a few surveys, but has not been universally adopted, partly for logistical reasons, but also because of concern about poor specificity and poor prediction of future risk of asthma in individuals without symptoms.^20^ However, the use of biological measurements to improve the validity of the asthma definition depends on the aim of the study. For instance, bronchial hyper-responsiveness has similar or better specificity, but much worse sensitivity, than symptom questionnaires, making it a less suitable method for the measurement of prevalence.^21^, ^22^

Misclassification and varying case definitions are commonly encountered in population health measurement.^2^ A key component of the Global Burden of Disease (GBD) analyses is to identify and correct for such sources of measurement bias.

In this Article, we present the results of estimating mortality, prevalence, and disease burden in DALYs and years lived with disability (YLDs) for COPD and asthma from the GBD 2015 study. We also report on the attribution of risk factors for these diseases and the relation between disease burden and the Socio-demographic Index (SDI), a compound measure of income, years of education, and total fertility rate.

## Methods

### Mortality

The methods of the GBD 2015 study have been extensively reported elsewhere.^1^, ^2^, ^23^ Briefly, deaths, incidence, prevalence, and DALY rates were estimated for 310 diseases and injuries for 195 countries and territories by age group and sex from 1990 to 2015. All-cause mortality was derived from vital registration systems, censuses, and surveys, and analysed with demographic methods to correct for incompleteness. Causes of death, derived from an extensive database of vital registration and verbal autopsy data, were analysed using GBD's Cause Of Death Ensemble modeling (CODEm) tool to calculate mixed effects or spatiotemporal Gaussian process regression models of rates or cause fractions with varying combinations of predictive covariates. Predictive validity testing determined the optimal ensemble of models. Covariates included smoking prevalence, cigarettes per capita, the proportion of the population exposed to household air pollution, mean exposure to ambient particulate matter (defined as the population-weighted annual average mass concentration of particles with a diameter less than 2·5 μm [PM_2·5_] in a m^3^ of air) from outdoor air pollution, a scalar of the combined exposure to risks for COPD (and asthma), and SDI. Because the sensitivity of verbal autopsy algorithms to detect specific chronic respiratory diseases is poor, we only modelled data on deaths from all chronic respiratory diseases in CODEm and constrained the estimates for specific chronic respiratory diseases to the estimates for all chronic respiratory deaths. We constrained estimates for all individual causes to the all-cause mortality rates derived from demographic estimation.

### Non-fatal estimation for COPD

Non-fatal estimates for COPD were based on systematic reviews of published papers, unpublished reports, surveys available in GBD's Global Health Data Exchange repository, and health service encounter data from the USA (coded in International Classification of Diseases [ICD]-9 to 490–492, 494, and 496). We used 7301 prevalence datapoints and 22 incidence datapoints covering 15 of 21 GBD world regions. No data were available for Andean Latin America, the Caribbean, central Asia, central and east sub-Saharan Africa, and Oceania. We used the Global Initiative for Chronic Obstructive Pulmonary Disease (GOLD) spirometry-based definition for COPD (a ratio of FEV_1_:FVC <0·70 after bronchodilation)^14^ and modelled overall prevalence and the proportions in COPD spirometry stages mild (FEV_1_ ≥80% of normal), moderate (FEV_1_ 50–79% of normal), and severe or very severe combined (FEV_1_ <50% of normal) in DisMod-MR 2.1, a Bayesian meta-regression tool. DisMod-MR 2.1 takes all available data on prevalence, incidence, remission (defined in GBD as the cure rate), and cause of death rates jointly into account and forces a consistent set of estimates for each parameter. Before entering data into DisMod-MR 2.1, we adjusted survey data using different spirometry case definitions. We adjusted datapoints from 14 studies reporting on the GOLD case definition without a bronchodilator after fitting an exponential curve to age-specific ratios of both measurements from three studies.^24^, ^25^, ^26^ Using a similar approach, we adjusted datapoints from six studies reporting LLN pre-bronchodilator data based on one study,^24^ three studies with LLN post-bronchodilator data based on five studies,^12^, ^24^, ^27^, ^28^, ^29^ and two studies using an older version of LLN by the European Respiratory Society based on two studies.^30^, ^31^ We used the meta-regression component of DisMod-MR 2.1 to determine an adjustment factor for data based on physician diagnosis and the US health service encounter data. We included a scalar for the combined exposure to all risks estimated for COPD as a predictive covariate. We included corresponding datapoints for excess mortality rate estimated as the ratio of cause-specific mortality rate and prevalence corresponding to the same year and age range of the datapoint. We used lag-distributed income per capita as a predictive covariate for excess mortality, forcing a negative coefficient on the assumption that case fatality decreases with increasing wealth in a country. Prevalence by GOLD class was available from only 24 countries in 14 GBD world regions.

The proportions of people in GOLD classes I, II, and III or IV were modelled separately in DisMod-MR 2.1 and then scaled to a sum of 1 and multiplied by the overall prevalence of COPD. In GBD, severity of COPD is classified into health states (appendix p 4). To map the prevalence by GOLD class into health states representing symptoms, we used the Medical Expenditure Panel Survey (MEPS) data^32^ for 2001–11 from the USA. MEPS is an ongoing data collection project with new panels recruited every 2 years. Respondents report on all health service contacts and the reasons for those contacts. We identified individuals with an ICD-9 diagnosis of COPD. We translated scores from a generic quality-of-life instrument, the 12-Item Short Form Health Survey (SF-12),^33^ into GBD disability weight values based on convenience samples of research fellows at the Institute for Health Metrics and Evaluation and annual GBD workshop participants filling in SF-12 for a selection of 60 of the 235 health states used in GBD 2015. Health states were presented as lay descriptions that had been the basis of the pairwise comparisons presented to respondents to the GBD disability weight surveys. After controlling for comorbidity, we assigned a specific disability weight to each individual with a diagnosis of COPD. We categorised cases into asymptomatic (disability weight value of 0), mild COPD (disability weight value between 0 and the midpoint of GBD disability weights for mild and moderate COPD), moderate COPD (disability weight value greater than the midpoint between mild and moderate and midpoint between moderate and severe COPD disability weights), and severe COPD (the remainder). We took the prevalence estimates for the USA in 2005 (at the midpoint of MEPS data range) and mapped the distribution of cases by GOLD classes into the distribution of severity from MEPS (appendix p 5). This gave us a mapping from GOLD class into GBD health states, which could then be applied to the prevalence data by GOLD class from all other countries and time periods.

### Non-fatal estimation for asthma

The main data sources for asthma were population surveys and US health service encounter data on the diagnoses for any health service contact for 42 million people. We used 9219 prevalence, 29 incidence, and 32 remission datapoints and population death rates from asthma estimated in CODEm and scaled to total death rates with all other cause-specific estimates. Data on prevalence were available for 121 countries covering all 21 GBD world regions. Our case definition for asthma was a reported diagnosis by a physician, with wheezing in the past 12 months. In DisMod-MR 2.1, we adjusted data based on reported wheezing only and US health service encounter data, and used a scalar of the combined exposure to risk factors for asthma. Similar to the COPD model, we added excess mortality rates corresponding to all prevalence datapoints with lag-distributed income per capita as a predictive covariate. The health states and disability weights for three asthma health states are listed in the appendix (p 4). The distribution between the three asthma health states and an asymptomatic health state was analysed in MEPS. In the absence of comparable epidemiological severity distribution data, a simplifying assumption had to be made that the US distribution of severity for asthma can be generalised to all countries. Additional details on the estimation process for COPD and asthma can be found in the appendix (pp 15–28).

### Risk estimation

Estimates were made of six risk factors for COPD (smoking, second-hand smoke, household air pollution, ambient particulate matter, ozone, and occupational particulates) and two risk factors for asthma (smoking and occupational asthmagens). Sufficient evidence of causality, availability of exposure data, potential for modification, and policy interest are criteria for choosing risks and associated outcomes in GBD. Population-attributable fractions of disease outcomes were estimated from exposure data, relative risks of outcomes, and a theoretical minimum level of exposure. Population surveys were the main source of exposure data on smoking, second-hand smoke, and household air pollution. Exposure to PM_2·5_ was measured from satellite data on aerosols in the atmosphere and calibrated to observations from ground monitors. We based exposure to ozone on a chemical transport model of satellite data.^34^ Occupational exposures were based on the proportion of the working population exposed to asthmagens and particulates based on distribution of the population in nine occupational groups as reported by the International Labor Organization.^35^ We derived relative risks from meta-analyses of cohort studies. The theoretical minimum exposure level was set as zero for smoking, second-hand smoke, and the occupational exposures. For household air pollution, the minimum was defined as no household reporting use of solid fuel for cooking. For ambient particulate matter, the minimum was set as a uniform distribution between the lowest and fifth percentile exposure level from all data sources. For ozone, the minimum was set as a uniform distribution between the lowest and fifth percentile exposure measured in the American Cancer Society's Cancer Prevention Study II.^36^ Unlike disease estimates that are mutually exclusive and collectively exhaustive in GBD, risk estimates are based on a counterfactual analysis (what if past exposure to a risk had been at the theoretical minimum level?) and are, therefore, not additive. Estimates of combinations of risks take mediation into account based on the difference in relative risks from cohort and trial data that did and did not control for another risk as a confounder. After adjustment for mediation, risks were combined using a multiplicative function to avoid the sum of risks exceeding the total amount of disease.^23^ Additional details on the estimation process for COPD and asthma risks can be found in the appendix (pp 29–57).

### DALY estimation

We calculated years of life lost (YLLs) by multiplying the number of deaths for a cause by the remaining life expectancy in GBD's standard life table based on the lowest observed mortality rates at each age in any population over 5 million.^1^ We calculated YLDs by multiplying the prevalence of each sequela by the disability weight that quantifies the relative severity of the sequela on a scale between 0 and 1. We derived disability weights from nine population surveys and an open-access internet survey using pairwise comparison methods.^37^ DALYs are the sum of YLLs and YLDs. We estimated uncertainty by recalculating every outcome of interest 1000 times, drawing from distributions of the sampling error around input data, corrections for measurement error, and estimates of residual non-sampling error and, in the case of cause of death estimates, model selection. Uncertainty intervals (UIs) were defined as the 25th and 975th values of the posterior distributions. We computed differences between estimates at the 1000-draw level and reported them as significant if more than 95% of values for the difference were either positive or negative. We computed age-standardised rates using the GBD standard population.^1^

SDI is an index of sociodemographic development consisting of lagged distributed income per capita, mean years of education over the age of 15 years, and total fertility rate.^1^ Each component was given equal weight and rescaled from 0 (for the lowest value observed during 1980–2015) to 1 (for the highest value observed) for income per capita and average years of schooling, and the reverse for the total fertility rate. The final SDI score was computed as the geometric mean of each of the components. We classified countries into five quintiles based on the entire distribution of location-year combinations between 1980 and 2015. We present results on each country's position based on its 2015 SDI value. A LOESS regression on all data from 1980 to 2015 was done to define the expected relationship between SDI and each health outcome. We contrast observed disease rates against this expected level to identify world regions performing better or worse than expected based on their development status.

This study is compliant with the Guidelines for Accurate and Transparent Health Estimates Reporting (GATHER) with details provided in the appendix (pp 58–60).^38^

### Role of the funding source

This research was supported by funding from the Bill & Melinda Gates Foundation. The funders had no role in the study design, data collection and analysis, interpretation of data, decision to publish, or preparation of the manuscript.

## Results

In 2015, 3·2 million people (95% UI 3·1 million to 3·3 million) died from COPD worldwide, an increase of 11·6% (5·3–19·8) compared with 1990, despite a decrease in the age-standardised death rate of 41·9% (37·7–45·1). Population growth and ageing of the global population outweighed the downward trend in age-standardised death rates. The greatest reduction in age-standardised death rates occurred in countries in the high-middle-SDI quintile and middle-SDI quintile. From 1990 to 2015, the prevalence of COPD increased by 44·2% (95% UI 41·7–46·6) to 174·5 million individuals (160·2 million to 189·0 million). The decrease in age-standardised prevalence of 14·7% (13·5–15·9) was much smaller than the decrease in age-standardised death rates. The greatest decrease in age-standardised prevalence was seen in countries in the high-middle-SDI quintile and the middle-SDI quintile (table 1).

**Table 1:** Deaths due to asthma and COPD and number of prevalent cases of disease in 2015 and percentage change in all-age and age-standardised rates in locations grouped by SDI quintile

	**Number of deaths (thousands)**	**Percentage change in all-age deaths, 1990–2015**	**Percentage change in age-standardised death rates, 1990–2015**	**Number of prevalent cases (thousands)**	**Percentage change in all-age prevalence, 1990–2015**	**Percentage change in age-standardised prevalence, 1990–2015**
**COPD**						
Global	3188 (3084 to 3293)	11·6 (5·3 to 19·8)	−41·9 (−45·1 to −37·7)	174 483 (160 205 to 188 952)	44·2 (41·7 to 46·6)	−14·7 (−15·9 to −13·5)
High SDI quintile	482 (468 to 505)	31·6 (27·8 to 38·2)	−26·2 (−28·2 to −22·5)	43 105 (39 912 to 46 414)	35·3 (31·8 to 39·1)	−7·3 (−9·3 to −4·9)
High-middle SDI quintile	626 (602 to 651)	−11·1 (−17·4 to −4·3)	−57·8 (−60·8 to −54·7)	44 923 (41 215 to 48 803)	42·3 (39·3 to 45·1)	−20·2 (−21·5 to −19·0)
Middle SDI quintile	1110 (1055 to 1169)	−3·4 (−11·0 to 5·8)	−53·5 (−57·2 to −49·2)	52 209 (47 430 to 57 154)	104·8 (101·6 to 108·0)	−22·6 (−23·9 to −21·4)
Low-middle SDI quintile	907 (850 to 965)	51·5 (24·1 to 89·5)	−25·7 (−38·1 to −7·9)	30 058 (27 495 to 32 719)	74·3 (71·6 to 77·2)	−3·7 (−4·8 to −2·7)
Low SDI quintile	61 (52 to 71)	68·8 (40·3 to 111·3)	−16·3 (−30·1 to 3·5)	4223 (3795 to 4656)	36·1 (33·2 to 38·9)	−1·6 (−3·1 to −0·1)
**Asthma**						
Global	397 (363 to 439)	−26·7 (−43·7 to 7·2)	−58·8 (−69·0 to −39·0)	358 198 (323 134 to 393 466)	12·6 (9·0 to 16·4)	−17·7 (−19·9 to −15·1)
High SDI quintile	22 (20 to 24)	−53·2 (−56·8 to −48·9)	−71·8 (−73·7 to −69·5)	63 883 (59 724 to 68 309)	−13·8 (−17·0 to −10·2)	−26·0 (−28·4 to −23·0)
High-middle SDI quintile	54 (50 to 61)	−3·2 (−12·9 to 9·0)	−49·4 (−54·9 to −43·0)	76 935 (69 650 to 84 654)	8·4 (4·1 to 13·0)	−15·2 (−18·0 to −12·1)
Middle SDI quintile	120 (110 to 132)	−12·2 (−28·7 to 16·0)	−38·4 (−49·5 to −19·4)	91 375 (82 505 to 100 370)	8·3 (4·2 to 12·6)	−14·5 (−16·3 to −12·3)
Low-middle SDI quintile	159 (136 to 186)	−40·5 (−61·4 to 19·0)	−69·6 (−81·3 to −32·9)	90 605 (79 887 to 101 371)	28·9 (24·6 to 33·4)	−18·4 (−20·7 to −15·9)
Low SDI quintile	41 (34 to 51)	22·1 (2·1 to 55·4)	−54·3 (−63·9 to −38·3)	35 011 (30 065 to 40 255)	94·8 (86·2 to 106·1)	−7·3 (−11·2 to −3·0)

Data in parentheses are 95% uncertainty intervals. SDI is calculated for each location (all 188 countries, seven territories, and 519 subnational locations estimated in GBD 2015) as a function of lag-distributed income per capita, average educational attainment in the population aged over 15 years, and the total fertility rate. SDI of 0 represents the lowest level of income per capita, educational attainment, and highest total fertility rate observed from 1980 to 2015, and SDI of 1 represents the highest income per capita, educational attainment, and lowest total fertility rate with an effect on health over the same period. Cutoffs on the SDI scale for the quintiles have been selected based on their 2015 values by location. COPD=chronic obstructive pulmonary disease. SDI=Socio-demographic Index.

In 2015, 0·40 million people (95% UI 0·36 million to 0·44 million) died from asthma, a decrease of 26·7% (−7·2 to 43·7) compared with 1990. The decrease in age-standardised death rates was 58·8% (39·0–69·0) between 1990 and 2015. The greatest reduction in age-standardised death rates occurred in countries in the high-SDI and low-middle-SDI quintiles. From 1990 to 2015, the prevalence of asthma increased by 12·6% (9·0–16·4) to 358·2 million individuals (323·1 million to 393·5 million). The decrease in age-standardised prevalence by 17·7% (15·1–19·9) was smaller than the overall decrease in age-standardised death rates. The age-standardised death rate for asthma in 2015 was higher in males (6·7 [5·9–7·5] per 100 000 people) than in females (5·6 [4·8–6·4] per 100 000 people). A greater reduction in age-standardised prevalence was seen in countries in the high-SDI and low-middle-SDI quintiles (table 1).

Globally, COPD affected 104·7 million males (95% UI 96·0 million to 113·8 million) and 69·7 million females (64·2 million to 75·4 million) in 2015. Age-standardised prevalence was 3·2% (2·9–3·5) in males and 2·0% (1·8–2·1) in females. Age-standardised DALY rates in males (1273·0 [95% UI 1215·5–1328·3] per 100 000 people) were almost twice as high as those in females (717·4 [677·7–759·3] per 100 000 people) reflecting a higher male-to-female ratio for deaths than for prevalence. Conversely, age-standardised DALY rates due to asthma were similar between male individuals (365 [290–451] per 100 000 people) and female individuals (368 [286–461] per 100 000 people). In 2015, more females (190·2 million [172·2 million to 208·9 million]) than males (168·0 million [150·8 million to 185·1 million]) had asthma; a reversal of the higher male-to-female ratio during adolescence.

YLLs contributed more than 80% of DALYs due to COPD. Conversely, asthma is highly prevalent at all ages and leads to fewer deaths than COPD and thus YLDs formed the larger component of DALYs, at just over 60%. The 63·9 million DALYs (95% UI 61·2 million to 66·3 million) due to COPD represented 2·6% (95% UI 2·4–2·8) of the entire global burden of disease in 2015. 26·2 million DALYs (20·5 million to 32·6 million) due to asthma contributed 1·1% (0·9–1·3) of the total burden in 2015 (table 2). The greatest decrease in age-standardised DALY rates due to COPD occurred in countries in the high-middle-SDI and middle-SDI quintiles. The biggest reduction in age-standardised asthma DALY rates occurred in the low-middle-SDI quintile (table 2).

**Table 2:** YLLs, YLDs, and DALYs due to asthma and COPD in 2015 and percentage change in all-age counts and age-standardised DALY rates from 1990 to 2015 in locations grouped by SDI quintiles

	**Number of YLLs, all ages (thousands)**	**Number of YLDs, all ages (thousands)**	**Number of DALYs, all ages (thousands)**	**Percentage change in DALYs, 1990–2015, all ages**	**Percentage change in age-standardised DALY rates, 1990–2015**
**COPD**					
Global	51 803 (49 898 to 53 611)	12 047 (10 207 to 13 725)	63 850 (61 215 to 66 289)	−1·0 (−7·1 to 6·2)	−43·7 (−47·0 to −39·8)
High SDI quintile	5914 (5762 to 6180)	2214 (1890 to 2545)	8128 (7755 to 8530)	12·7 (9·9 to 16·6)	−28·2 (−30·1 to −25·9)
High-middle SDI quintile	9058 (8693 to 9446)	2500 (2103 to 2882)	11 661 (11 093 to 12 226)	−19·8 (−25·0 to −14·2)	−58·5 (−61·2 to −55·6)
Middle SDI quintile	17 918 (16 979 to 18 887)	4050 (3422 to 4623)	21 812 (20 738 to 22 908)	−16·4 (−22·3 to −9·2)	−55·8 (−58·9 to −52·1)
Low-middle SDI quintile	17 444 (16 260 to 18 652)	2954 (2493 to 3345)	20 399 (19 079 to 21 673)	32·0 (8·0 to 61·0)	−27·0 (−40·0 to −10·6)
Low SDI quintile	1433 (1203 to 1685)	374 (316 to 429)	1806 (1559 to 2064)	55·7 (31·3 to 86·9)	−18·0 (−30·5 to −0·5)
**Asthma**					
Global	10 270 (9369 to 11 448)	15 899 (10 371 to 22 344)	26 169 (20 501 to 32 583)	−14·6 (−26·0 to 2·1)	−42·8 (−52·0 to −29·5)
High SDI quintile	384 (366 to 408)	2818 (1838 to 3905)	3203 (2221 to 4299)	−25·4 (−29·9 to −21·9)	−35·9 (−40·2 to −32·6)
High-middle SDI quintile	1227 (1130 to 1400)	3419 (2228 to 4795)	4766 (3508 to 6154)	−3·8 (−9·1 to 0·9)	−30·3 (−35·6 to −25·9)
Middle SDI quintile	2912 (2655 to 3223)	4061 (2657 to 5704)	6855 (5464 to 8453)	−12·5 (−22·9 to −0·7)	−40·7 (−49·3 to −30·2)
Low-middle SDI quintile	4327 (3728 to 5073)	4020 (2621 to 5682)	8350 (6705 to 10 088)	−27·4 (−44·6 to 9·1)	−60·8 (−71·8 to −31·9)
Low SDI quintile	1402 (1162 to 1692)	1563 (1009 to 2238)	2961 (2343 to 3687)	45·9 (27·2 to 67·9)	−31·4 (−41·2 to −18·4)

Data in parentheses are 95% uncertainty intervals. YLLs=years of life lost. YLDs=years lived with disability. DALYs=disability-adjusted life years. COPD=chronic obstructive pulmonary disease. SDI=Socio-demographic Index.

**Figure 1:**
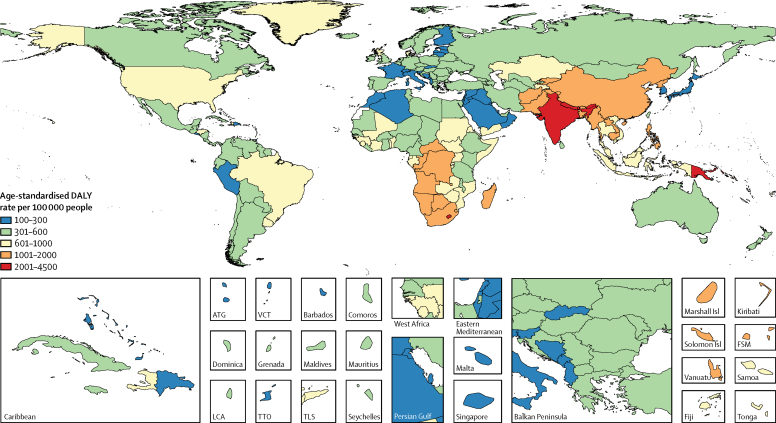
Age-standardised DALY rate per 100 000 people due to COPD by country, both sexes, 2015 DALYs=disability-adjusted life years. COPD=chronic obstructive pulmonary disease. ATG=Antigua and Barbuda. FSM=Federated States of Micronesia. Isl=islands. LCA=Saint Lucia. TLS=Timor-Leste. TTO=Trinidad and Tobago. VCT=Saint Vincent and the Grenadines.

**Figure 2:**
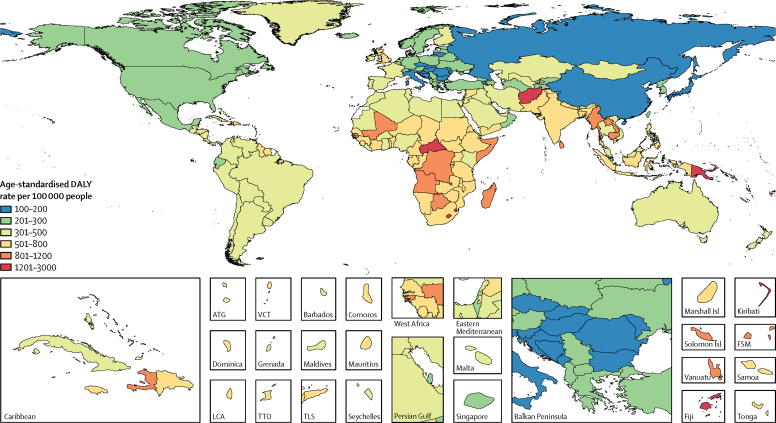
Age-standardised DALY rate per 100 000 people due to asthma, by country, both sexes, 2015 DALYs=disability-adjusted life years. ATG=Antigua and Barbuda. FSM=Federated States of Micronesia. Isl=islands. LCA=Saint Lucia. TLS=Timor-Leste. TTO=Trinidad and Tobago. VCT=Saint Vincent and the Grenadines.

Age-standardised DALY rates due to COPD in 2015 were estimated to exceed 2000 per 100 000 people in Papua New Guinea, India, Lesotho, and Nepal. Rates below 300 per 100 000 people were seen in some countries in high-income Asia Pacific, central Europe, north Africa and Middle East, the Caribbean, western Europe, and Andean Latin America (figure 1). Age-standardised asthma DALY rates in excess of 1200 per 100 000 people were estimated for Afghanistan, Central African Republic, Fiji, Kiribati, Lesotho, Papua New Guinea, and Swaziland. Countries in eastern and central Europe, China, Italy, and Japan had asthma DALY rates between 100 and 200 per 100 000 people (figure 2). DALY estimates for COPD and asthma by country and the percentage change in DALYs and age-standardised DALY rates between 1990 and 2015 are presented in the appendix (p 61–67).

**Figure 3:**
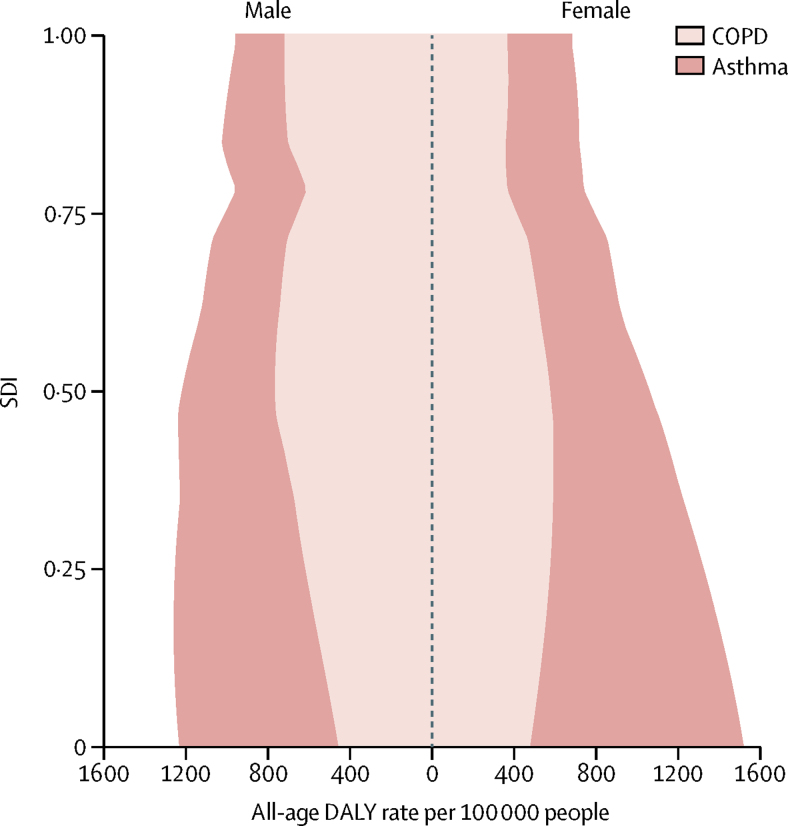
Expected relationship between all-age DALY rates due to COPD and asthma and SDI by sex, 2015 DALYs=disability-adjusted life years. COPD=chronic obstructive pulmonary disease. SDI=Socio-demographic Index.

**Figure 4:**
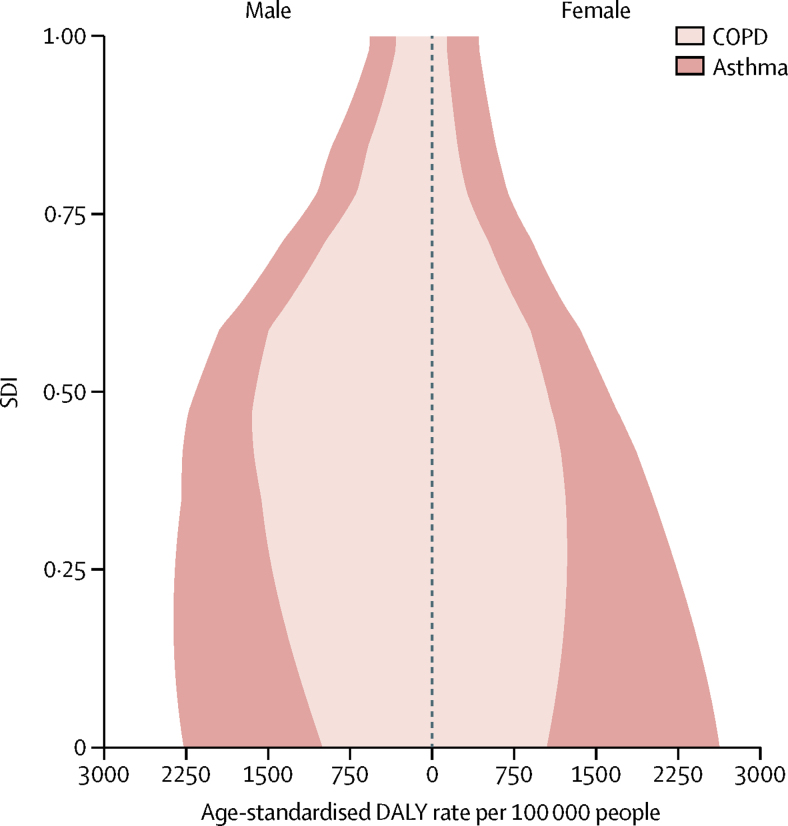
Expected relationship between age-standardised DALY rates due to COPD and asthma and SDI by sex, 2015 DALYs=disability-adjusted life years. COPD=chronic obstructive pulmonary disease. SDI=Socio-demographic Index.

**Figure 5:**
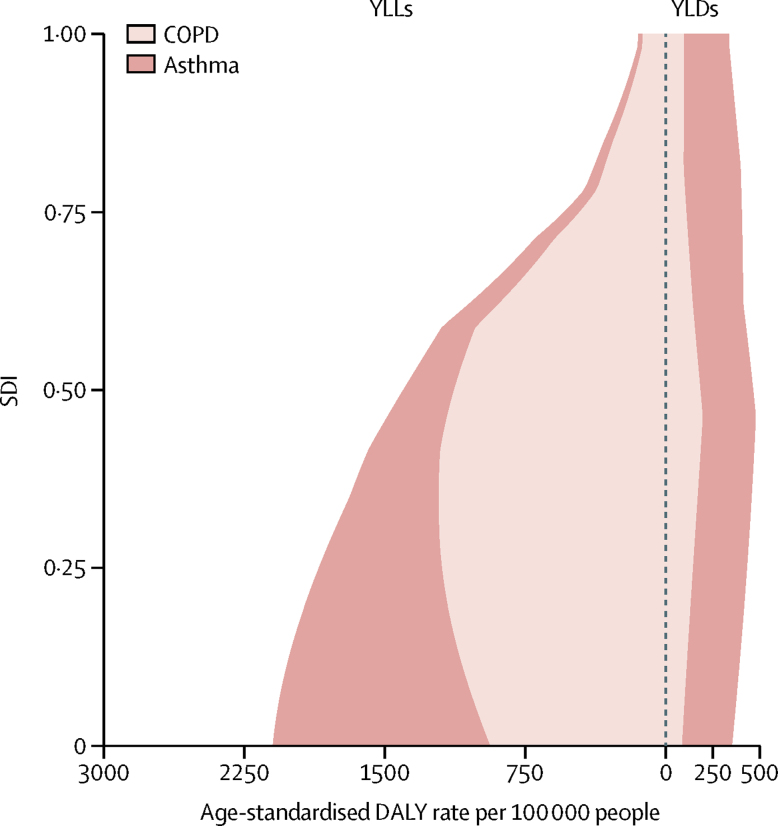
Expected relationship between age-standardised DALY rates due to COPD and asthma and SDI by YLLs and YLDs, 2015 DALYs=disability-adjusted life years. COPD=chronic obstructive pulmonary disease. SDI=Socio-demographic Index. YLLs=years of life lost. YLDs=years lived with disability.

Examining the expected relationship between SDI and all-age DALY rates showed a reduction in asthma rates with increasing SDI in both sexes, whereas DALY rates due to COPD increased up until around 0·5 SDI, decreased to the lowest values at an SDI value of 0·75, after which they slightly increased (figure 3). These patterns reflect a combined effect of population growth, ageing, and variation in prevalence. The change in age-standardised DALY rates with SDI shows an increase in DALY rates due to COPD until the middle range of SDI values and then a sharp decline. DALY rates due to asthma in both sexes decreased monotonically with rising SDI (figure 4). The relationship between DALY rates due to asthma and SDI largely reflected variation in YLLs, whereas DALY rates due to COPD varied similarly for YLLs and YLDs across the SDI continuum (figure 5).

**Figure 6:**
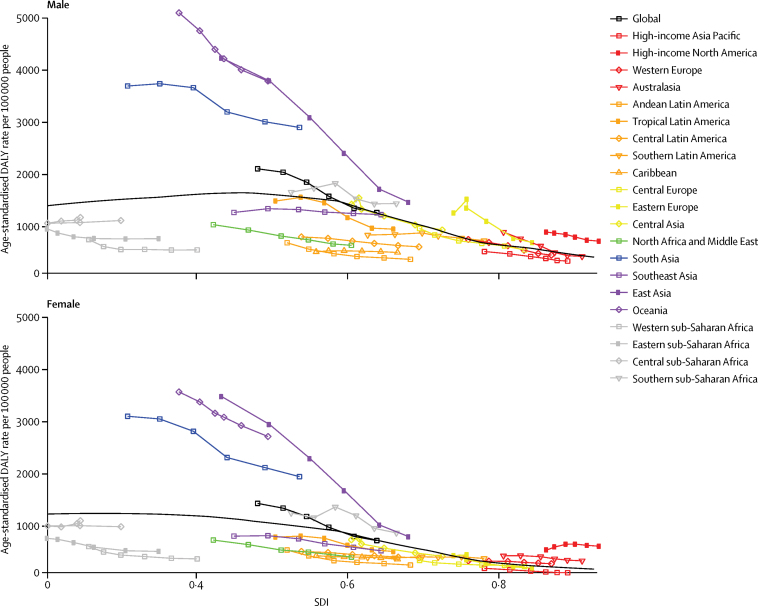
Age-standardised DALY rates due to COPD by 21 GBD world regions and the expected value based on the SDI by sex, 1990–2015 The black line represents the expected value of a disease rate based on a LOESS regression of all years of estimates by GBD locations and their SDI value. DALYs=disability-adjusted life years. COPD=chronic obstructive pulmonary disease. GBD=Global Burden of Disease. SDI=Socio-demographic Index. LOESS=locally weighted regression and smoothing scatterplots.

**Figure 7:**
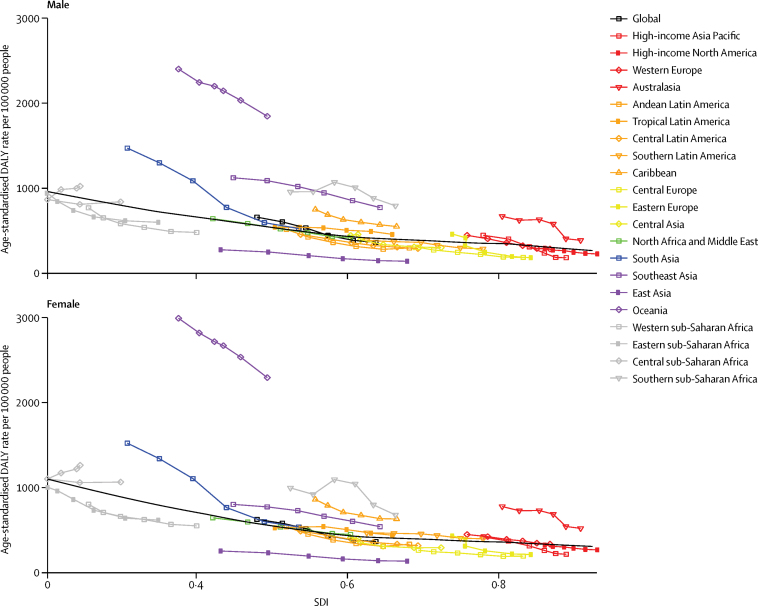
Age-standardised DALY rates due to asthma by 21 GBD world regions and the expected value based on the SDI by sex, 1990–2015 The black line represents the expected value of a disease rate based on a LOESS regression of all years of estimates by GBD locations and their SDI value. DALYs=disability-adjusted life years. GBD=Global Burden of Disease. SDI=Socio-demographic Index. LOESS=locally weighted regression and smoothing scatterplots.

The GBD regions of Oceania, east Asia, south Asia, and high-income North America had higher age-standardised DALY rates due to COPD in both sexes than expected based on their SDI. Male individuals in eastern Europe also had higher than expected DALY rates. Regions with better-than-expected COPD DALY rates included eastern, central, and western sub-Saharan Africa; central and Andean Latin America and the Caribbean; and north Africa and the Middle East (figure 6). Age-standardised DALY rates due to asthma in Oceania were much higher than expected based on SDI. Australasia, southeast Asia, the Caribbean, and southern sub-Saharan Africa also had higher DALY rates than expected. The asthma DALY rates in south Asia were higher than expected in 1990 (when SDI was lowest), but converged with expected values in 2015. Central Europe, east Asia, and western and eastern sub-Saharan Africa had lower than expected asthma DALY rates (figure 7).

**Figure 8:**
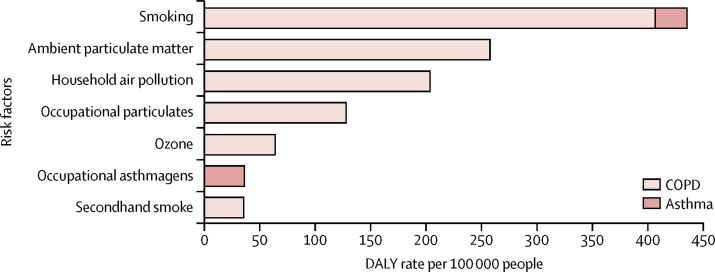
Age-standardised DALY rates due to COPD and asthma attributable to seven risk factors, both sexes, 2015 COPD=chronic obstructive pulmonary disease. DALYs=disability-adjusted life years.

**Figure 9:**
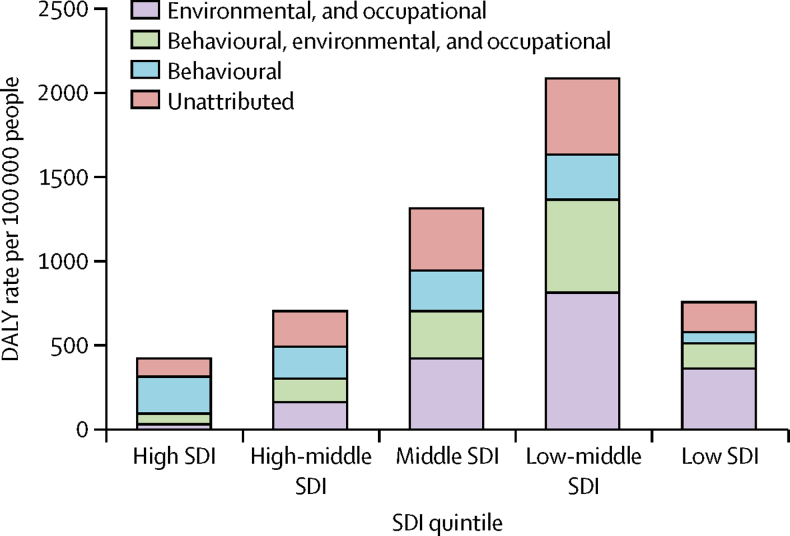
Contribution of behavioural and environmental and occupational risks to DALYs due to COPD per 100 000 people in locations grouped by SDI quintiles, 2015 Environmental and occupational: ambient particulate matter, household air pollution, occupational particulates, and ozone. Behavioural: smoking and second-hand smoke. Behavioural environmental: ambient particulate matter, household air pollution, occupational particulates, ozone, smoking, and second-hand smoke. DALYs=disability-adjusted life years. COPD=chronic obstructive pulmonary disease. SDI=Socio-demographic Index.

Smoking and ambient particulate matter were the main risks for COPD followed by household air pollution, occupational particulates, ozone, and second-hand smoke (figure 8). Together, these risks explained 73·3% (95% UI 65·8–80·1) of DALYs due to COPD. Smoking and occupational asthmagens were the only risks quantified for asthma in GBD, explaining just 16·5% (14·6–18·7) of the asthma DALYs. The contribution of risks to the burden of COPD varied by SDI quintiles. In high-SDI countries, the behavioural risks (smoking and second-hand smoke) were the most important, whereas environmental risks and, to a lesser extent, occupational risks explained most of the burden in lower-SDI quintiles. The proportions of COPD burden not explained by any of the GBD risks showed little variation between SDI quintiles (figure 9, table 3).

**Table 3:** Proportional contribution of behavioural and environmental and occupational risks for COPD in DALYs per 100 000 people in locations grouped by SDI quintiles, 2015

	**Percentage contribution from environmental and occupational risks**	**Percentage contribution from behavioural and environmental and occupational risks**	**Percentage contribution from behavioural risks**	**Percentage unattributed**
High SDI quintile	7·0	15·1	54·8	23·2
High-middle SDI quintile	23·5	20·3	27·1	29·2
Middle SDI quintile	32·5	21·3	17·5	28·7
Low-middle SDI quintile	37·0	26·3	12·7	24·0
Low SDI quintile	40·4	17·8	7·9	34·0

COPD=chronic obstructive pulmonary disease. DALYs=disability-adjusted life years. SDI=Socio-demographic Index.

More detailed GBD 2015 results are available for download online and in online visualisation tools, and the GBD 2015 code can be accessed online.

## Discussion

Asthma was the most prevalent chronic respiratory disease, affecting an estimated 358 million people in 2015. COPD was half as common, with 174 million people affected in 2015. Deaths from COPD were eight times more common than deaths from asthma. YLLs contributed 81·2% of the 63·8 million global DALYs due to COPD, whereas YLDs represented the largest proportion of the 26·2 million global DALYs due to asthma. COPD ranked eighth (2·6% of global DALYs) and asthma 23rd (1·1% of global DALYs) among the 315 GBD causes in 2015. Age-standardised DALY rates from COPD and asthma declined significantly by 43·7% (39·8–47·0) for COPD and by 42·8% (29·5–52·0) for asthma annually between 1990 and 2015. Most of the reductions have come from a reduction in mortality, by 41·9% for COPD and 58·8% for asthma. The reductions in YLDs have been much smaller. This finding reflects greater improvements in reducing case fatality rather than a change in incidence and prevalence. The absence of a relationship between asthma prevalence and asthma death rates, and our knowledge about asthma pathophysiology and clinical trial findings, add evidence that most asthma deaths at all ages are preventable by treatment with low-dose inhaled corticosteroids and other management strategies or, to a lesser extent, avoidance of risk factors. Indeed, the observed low asthma mortality in high-income countries reflects better access to health services and better treatment options following international asthma guidance.^39^ This fact is reflected by a strong relationship between SDI and mortality, but not prevalence of asthma. The relationship between SDI and COPD is less monotonic. Higher COPD death rates and prevalence at the middle range of SDI values reflect the increase in smoking and outdoor air pollution observed in countries going through the demographic and epidemiological transition.

Between the GBD 2013 and GBD 2015 iterations, a methodological change led to a significant difference in prevalence and YLDs due to COPD. In the GBD 2013 study, we estimated 328·5 million prevalent cases and 26·1 million YLDs for the year 2013, whereas the GBD 2015 estimates for 2015 were 174·5 cases and 12·0 million YLDs. This difference is due to a shift from taking LLN estimates as the reference case definition to using the fixed-ratio definition of GOLD. This change in the methods led to much lower prevalence estimates in 2015 than in 2013 because the GOLD criteria identified lower prevalence in younger adults than older adults. Younger adults represent a much larger proportion of the world's population, and therefore a higher estimate of prevalence at these ages affects the total prevalence more than an equivalent change in the prevalence at older ages. The main motivation to revert to the fixed-ratio GOLD definition for GBD 2015 was that we aimed to estimate symptomatic disease. Our severity distributions are derived from epidemiological data on GOLD classes, which fit better with the estimation of prevalence based on GOLD's fixed ratio criteria than LLN. Most of the arguments for using an LLN case definition are based on future risk of disease to identify people with early signs of disease, who could be prevented from developing symptomatic disease by measures such as smoking cessation. Both in primary and secondary care, clinicians rely on respiratory symptoms, exposure to major known risks, and airflow limitation to diagnose COPD clinically. Accurate LLN estimation requires prediction reference equations, and to date there are no universal prediction equations for LLN because spirometry is not only variable by age and sex, but is also race-related and affected by different local environments.^15^, ^40^ To date, the fixed ratio has gained more popular use in clinical practice because it is easy to calculate, thus helping to remove barriers to the widespread use of spirometry and diagnosis of fixed airflow obstruction. Furthermore, nearly all evidence on efficacy and safety of respiratory drugs and other treatments comes from randomised trials with patients identified using a fixed ratio definition.^41^

No comparable estimates of the global prevalence of COPD exist other than those made for previous iterations of GBD. A decade ago, findings from a meta-analysis^42^ of COPD prevalence studies showed large heterogeneity depending on case definitions based on spirometry, physician diagnosis, symptoms, and radiology. No attempt was made to pool estimates between different study methods and diagnostic thresholds. The various initiatives (BOLD, PLATINO, EPISCAN, and PREPOCOL) for population-representative COPD spirometry surveys have been combined for pooled analyses to compare prevalence estimates between sites, but estimation of global prevalence has not been attempted.^16^

An estimate of 300 million prevalence cases of asthma was made in 2004 as part of the Global Initiative for Asthma (GINA) based on the International Study of Asthma and Allergies in Childhood (ISAAC) and European Community Respiratory Health Survey (ECRHS) estimates of wheezing prevalence and an arbitrary 50% reduction of these estimates for clinical asthma.^43^ This estimate is a lower than our estimate of 329 million prevalent cases in 2000, and 327 million cases in 2005, and our current 2015 estimate of 358 million cases. A comparison between the ISAAC study estimates in children and the ECRHS study in adults showed a high correlation between childhood and adult prevalence estimates within countries, but large variation between countries.^18^ In an analysis of the World Health Surveys done in the early 2000s in 70 countries,^44^ estimates of the prevalence of wheeze, reported physician diagnosis, and clinical asthma (based on questions of a physician diagnosis and ever having been treated for asthma or currently using asthma medication) were pooled. This showed a difference of around two times between higher estimates for wheeze compared with a reported physician diagnosis, whereas prevalence of clinical asthma was only marginally higher than for physician diagnosis. However, the pooling method in this study was not explained.^44^ ISAAC Phase Three was completed in 233 centres in 97 countries (80% low-income and middle-income countries), and ISAAC repeated surveys of prevalence of asthma have been done in 106 centres in 56 countries to date. We have made use of all publicly available survey results from ISAAC and ECRHS.

We estimated the highest age-standardised DALY rates due to COPD in 2015 in Papua New Guinea, India, Lesotho, and Nepal. Findings from three verbal autopsy studies in the 1980s in Papua New Guinea showed high chronic respiratory disease mortality. We decided to retain these three studies as they provide the only data on causes of death for this country; there were no reasons to exclude them based on an assessment of their quality. The high rates of mortality and morbidity in Lesotho and Nepal were based on predictive covariates as we do not have primary data for COPD from these two countries. The high rates in India were driven by mortality data sources and two small spirometry studies in Pune and Mumbai.^45^

Our knowledge of the natural history of COPD and asthma is extensive yet incomplete. For asthma, over 100 cohorts focusing on asthma and allergy have been initiated worldwide over the past 30 years.^46^ These long-term birth cohort studies are essential to understanding the life course and childhood predictors of asthma and allergy and the complex interplay between genes and the environment (including lifestyle and socioeconomic determinants). However, information to quantify population-level exposure to allergens in a comparable manner is incomplete, and it has therefore not been possible to add it as a risk in GBD. Such natural history evidence is mostly missing for COPD.^47^

The contribution of modifiable risk factors to COPD is large, yet much less for asthma. There are preventive interventions to reduce exposure to smoking, second-hand smoke, air pollution, biomass for cooking or heating, occupational exposures, or any combination of these factors. Additionally, other risk factors have been identified such as parental or sibling history of asthma and atopy, low birthweight, lower respiratory infections in childhood, education, day care, pet ownership, and other exposures, among others suggested. However, we are still far from eliminating these as major contributors to the burden of COPD. Smoking is the largest contributor to the COPD burden in countries at the higher end of the SDI (69·4% of COPD burden in high-SDI quintile countries), whereas the proportion of COPD explained by environmental exposures is highest in countries with low SDI (58·1% of COPD burden in low-SDI quintile countries). Given the importance of smoking as a risk factor of disease, monitoring national and international trends and projections in smoking remains paramount for worldwide health surveillance.^48^, ^49^ Smoking prevalence has decreased in men and women since 1990 worldwide, but progress in tobacco control has not been universal.^50^ Globally, exposure to household air pollution from solid fuels has decreased since 1990, but exposure to ambient air pollution has increased since 1990.^23^

However, a considerable proportion of COPD remains unexplained and cannot be attributed to the risks quantified in GBD. In the next iteration of GBD, we plan to quantify a past history of pulmonary tuberculosis as an additional risk factor for COPD as there is growing evidence for a causal relationship.^51^, ^52^ We did not estimate air pollution as a risk for asthma, because we had insufficient evidence for an increased risk of disease. Repeated lower respiratory infections in childhood and the long-term effects of asthma have been reported as other explanatory factors, but it is not quite apparent how estimation of these effects can be operationalised in GBD.^53^, ^54^ In future GBD iterations, ambient and household air pollution will be re-evaluated as risk factors for asthma.^55^, ^56^

Other newly established individual risk factors of COPD, such as low level of physical activity, could also have contributed to the unexplained COPD burden, as it has been related both to an increased risk of COPD among smokers^57^ and to a higher risk of COPD mortality.^58^

We estimate only a small proportion of asthma burden due to risks quantified in GBD: 10·1% from occupational asthmagens and 7·8% due to smoking.^59^, ^60^ Evidence from long-term observational studies and birth cohorts have rendered three hypotheses on other causes and triggers of asthma, namely the hygiene, westernisation, and obesity or sedentarism hypotheses. Comparative studies^61^, ^62^ of rural and urban populations gave rise to the hygiene theory that exposure to infections in early childhood explains the lower prevalence of asthma in rural areas. The second theory is that socioeconomic development or westernisation predisposes to the development of asthma, but it is not clear which pathways other than those described in the hygiene theory have a role.^63^ Obesity has been linked to a higher prevalence of asthma in children^64^ and an increased risk of developing new asthma in adults.^65^

As concluded by Fuchs and colleagues in their 2017 Review,^66^ we need to better understand underlying mechanisms of associations of asthma onset or remission with risk and protective factors, and future asthma research should integrate both paediatric and adult populations and longitudinal studies.

The general limitations of GBD studies have been reported elsewhere and apply to estimates of obstructive airways disease as well,^1^, ^2^, ^23^ and there are a number of limitations specific to COPD and asthma. The first concerns the poor consensus on a case definition of COPD. There was a difference of more than two times in YLDs between the GBD 2013 study, which used LLN as its case definition, and the current study's YLD results based on the fixed ratio of the GOLD definition. As the survey data on GOLD class distributions is largely based on a fixed ratio estimate of overall prevalence, we advocate that for GBD estimation purposes, use of the fixed ratio is preferable. Additionally, defining the cutoff value for LLN as the fifth percentile in a healthy reference population makes the arbitrary assumption that prevalence cannot be lower than 5%.

Second, to make use of all spirometry surveys that reported COPD prevalence using different thresholds, and with or without bronchodilation, we had to adjust data sources to the expected values of our reference case definition. We used a limited set of surveys that presented data with the reference and alternative case definitions. Each of those adjustments showed a strong age pattern, which we tried to capture with regression methods. Such adjustments add uncertainty, which would be avoided if estimates were all reported in a standard manner.

Third, because no physiological measurement is considered a gold standard, diagnosis of asthma relies on clinical assessment and self-report, a physician diagnosis, or both. Thus, measurement of asthma prevalence can be affected by the limitations of recall bias, access to health services, and different interpretations of survey questions inherent in self-reported health measurements.^67^ Access to clinical care is a challenge in low-income and middle-income countries, as well as in rural settings, therefore, defining asthma by reported symptoms and a doctor diagnosis could lead to an underestimate of asthma prevalence. We refined our assessment of asthma studies in GBD 2015 to better deal with nuances in self-report measures and adjusted for three instead of just one non-reference case definition. However, we cannot exclude that some residual measurement bias has affected the comparability of estimates between countries.

Fourth, mapping severity in MEPS to COPD GOLD class prevalence assumes this relationship can be generalised from the USA to the rest of the world. However, we only use the relationship between epidemiological data on GOLD class distributions in the USA to the severity pattern of cases with COPD in MEPS as reflected in respondents' answers to the SF-12. Our epidemiological models of the GOLD class distribution allow us to differentiate severity by age, sex, year, and location in as far as the sparse information on GOLD class prevalence allows. Our measurements of COPD severity would benefit from increased use standardised measures in surveys that reflect the lay descriptions on which the GBD disability weights are based or that use a generic quality-of-life measure like SF-12.

Fifth, our measurement of asthma severity completely relies on MEPS data and therefore, unlike COPD, assumes the same distribution for every location, year, age, and sex. This assumption is highly unlikely as treatments have a large effect on severity of asthma. For this reason, we found no relationship between SDI and YLDs from asthma, counter to the expectation that increased access to treatment, particularly steroid inhalers, would impact asthma severity and hence disability. Researchers are encouraged, in future surveys, to collect information on the proportion of cases that would fall into the lay description categories for controlled, partially controlled, and uncontrolled asthma.

Sixth, for many countries in the world that do not have functional vital registration systems, we had to rely on death estimates of all chronic respiratory diseases from verbal autopsy studies because these studies cannot distinguish between asthma, COPD, or other chronic respiratory diseases. Initiatives to strengthen vital registration systems are key to improving population health measurement because verbal autopsy can only identify a restricted set of diseases.^68^

Seventh, the estimate of the global prevalence of asthma changed from 242 million in 2013 based on GBD 2013 to 358 million in 2015 for GBD 2015. In GBD 2015, cause-specific mortality rates were added to the DisMod-MR 2.1 model with income per capita as a covariate to differentiate excess mortality rates based on a country's income. This addition had little effect on the estimates in high-income countries, but increased estimates in low-income and middle-income countries considerably. We believe that this approach is an improvement in the estimation strategy and that future estimates of global prevalence of asthma will be more consistent with the GBD 2015 finding.

COPD and asthma are important contributors to the burden of non-communicable disease. Although much of the burden is either preventable or treatable with affordable interventions, these diseases have received less attention than other prominent non-communicable diseases like cardiovascular disease, cancer, or diabetes. Up-to-date population information on these common diseases is key to policy decision making to improve access to and quality of existing intervention strategies. We call for greater standardisation in data collection with regard to case definition and severity distributions of all non-communicable diseases in general, and of asthma and COPD in particular. More, and updated, population measurements of COPD and asthma are needed to better quantify the size of the problem, to benchmark with other chronic conditions associated with smoking and ageing, and with any other environmental and air pollution exposures.

Correspondence to: Prof Theo Vos, Institute for Health Metrics and Evaluation, University of Washington, Seattle, WA 98121, USA **tvos@uw.edu**

For the **GBD 2015 results** see http://ghdx.healthdata.org/gbd-results-tool

For the **online visualisation tools** see https://vizhub.healthdata.org/gbd-compare

For the **GBD 2015 code** see http://ghdx.healthdata.org/gbd-2015-code
