# Corrections

**DOI:** 10.1016/S2213-2600(17)30336-3

**Published:** 2017-10

*GBD 2015 Chronic Respiratory Disease Collaborators. Global, regional, and national deaths, prevalence, disability-adjusted life years, and years lived with disability for chronic obstructive pulmonary disease and asthma, 1990–2015: a systematic analysis for the Global Burden of Disease Study* 2015. Lancet Respir Med *2017; **5**: 691-706*—In this Article, Tsegaye T Gebrehiwot and Alan D Lopez should have been listed as authors. The affiliation details for Josep M Antó have been updated. The sixth sentence of the Discussion should read “Age-standardised DALY rates from COPD and asthma declined significantly by 43·7% (39·8–47·0) for COPD and by 42·8% (29·5–52·0) for asthma between 1990 and 2015”. These corrections have been made to the online version as of Sept 14, 2017.

