# Supplementary material for: Global, regional, and national deaths, prevalence, disability-adjusted life years, and years lived with disability for chronic obstructive pulmonary disease and asthma, 1990–2015: a systematic analysis for the Global Burden of Disease Study 2015
Source: Lancet Respir Med. 2017 Sep;5(9):691–706. doi: 10.1016/S2213-2600(17)30293-X (PMC5573769; doi:10.1016/S2213-2600(17)30293-X)
Supplement: Supplementary appendix [file mmc1.pdf]

THE LANCET
Respiratory Medicine

Supplementary appendix

This appendix formed part of the original submission and has been peer reviewed. We post it as supplied by the authors.

Supplement to: GBD 2015 Chronic Respiratory Disease Collaborators. Global, regional, and national deaths, prevalence, disability-adjusted life years, and years lived with disability for chronic obstructive pulmonary disease and asthma, 1990–2015: a systematic analysis for the Global Burden of Disease Study 2015. *Lancet Respir Med* 2017; published online August 16. http://dx.doi.org/10.1016/S2213-2600(17)30293-X.

Appendix for Global, regional, and national deaths, prevalence, disability-adjusted life years, and years lived with disability for chronic obstructive pulmonary disease and asthma, 1990–2015: a systematic analysis for the Global Burden of Disease Study 2015

Preamble

This appendix provides further methodological detail and more detailed results for Global, regional, and national deaths, prevalence, disability-adjusted life years, and years lived with disability for chronic obstructive pulmonary disease and asthma, 1990-2015: a systematic analysis for the Global Burden of Disease study 2015. This study complies with the Guidelines for Accurate and Transparent Health Estimates Reporting (GATHER) recommendations. It includes detailed tables and information on data in an effort to maximize transparency in our estimation processes and provide a comprehensive description of analytical steps.

Table of Contents

Preamble 2

Appendix Table 1. Health states, lay descriptions, and disability weight values for COPD and asthma 4

Appendix Figure 1. Mapping of USA 2005 distribution of GOLD classes I, II, and III and IV combined into distribution of asymptomatic, mild, moderate, and severe COPD in Medical Expenditure Panel Surveys, 2001 to 2009…. 5

DisMod-MR 2.1 Estimation 6

Spatiotemporal Gaussian process regression 10

Fatal chronic respiratory diseases estimation process 15

Fatal chronic obstructive pulmonary disease estimation process 17

Fatal asthma estimation process 19

Non-fatal chronic obstructive pulmonary disease estimation process 20

Non-fatal asthma estimation process 25

Smoking risk factor estimation process 29

Ambient particulate matter risk factor estimation process 34

Household air pollution risk factor estimation process 44

Occupational risk factor estimation process 46

Ozone risk factor estimation process 53

Secondhand smoke risk factor estimation process 55

Appendix Table 2. GATHER checklist of information that should be included in reports of global health estimates, with description of compliance and location of information for GBD 2015 paper on COPD and asthma 58

Appendix Table 3. DALYs caused by COPD and asthma in 2015 and percent change in all-age numbers and age-standardised rates between 1990 and 2015, by location 61

Appendix Table 1. Health states, lay descriptions, and disability weight values for COPD and asthma in GBD

Health state	Lay description	Disability weight (95% UI)
**COPD, mild**	Has cough and shortness of breath after heavy physical activity, but is able to walk long distances and climb stairs.	0.019 (0.011–0.033)
**COPD, moderate**	Has cough, wheezing, and shortness of breath, even after light physical activity. The person feels tired and can walk only short distances or climb only a few stairs.	0.225 (0.153–0.31)
**COPD, severe**	Has cough, wheezing, and shortness of breath all the time. The person has great difficulty walking even short distances or climbing any stairs, feels tired when at rest, and is anxious.	0.408 (0.273–0.556)
**Asthma, controlled**	Has wheezing and cough once a month, which does not cause difficulty with daily activities.	0.015 (0.007–0.026)
**Asthma, partially controlled**	Has wheezing and cough once a week, which causes some difficulty with daily activities.	0.036 (0.022–0.055)
**Asthma, uncontrolled**	Has wheezing, cough, and shortness of breath more than twice a week, which causes difficulty with daily activities and sometimes wakes the person at night.	0.133 (0.086–0.192)

Appendix Figure 1: Mapping of USA 2005 distribution of GOLD classes I, II, and III and IV combined into distribution of asymptomatic, mild, moderate, and severe COPD in Medical Expenditure Panel Surveys, 2001 to 2009.

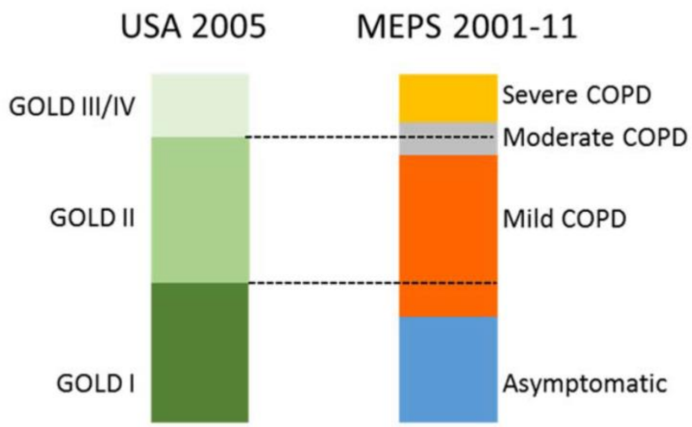

DisMod-MR 2.1 estimation

a. Estimation of sequelae and causes

The most extensively used estimation method is the Bayesian meta-regression method DisMod-MR 2.1. For some causes such as HIV/AIDS or hepatitis B and C, disease-specific natural history models have been used where the underlying three-state model in DisMod-MR 2.1 (susceptible, cases, dead) is insufficient to capture the complexity of a disease process. For some diseases with a range of sequelae differentiated by severity, such as chronic obstructive pulmonary disease (COPD) or diabetes mellitus, DisMod-MR 2.1 is used to meta-analyze the data on overall prevalence. Separate DisMod-MR 2.1 models are then used to analyze data on the proportion of cases with different severity levels or sequelae. Likewise, DisMod-MR 2.1 is used to meta-analyze data on the proportions of liver cancer and cirrhosis due to underlying etiologies such as hepatitis B, hepatitis C, and alcohol use.

b. DisMod-MR 2.1 description

Until GBD 2010, non-fatal estimates in burden of disease assessments were based on a single data source on prevalence, incidence, remission, or a mortality risk selected by the researcher as most relevant to a particular geography and time. For GBD 2010, we set a more ambitious goal: to evaluate all available information on a disease that passes a minimum quality standard. That required a different analytical tool that would be able to pool disparate information presented in varying age groupings and from data sources using different methods. The DisMod-MR 1.0 tool used in GBD 2010 evaluated and pooled all available data, adjusted data for systematic bias associated with methods that varied from the reference and produced estimates by world regions with uncertainty intervals using Bayesian statistical methods. For GBD 2013, the improved DisMod-MR 2.0 had increased computational speed allowing computations that were consistent between all disease parameters at the country rather than region level. The hundred-fold increase in speed of DisMod-MR 2.0 was partly due to a more efficient rewrite of the code in C++ but also by changing to a model specification using log rates rather than a negative binomial model used in DisMod-MR 1.0. In cross-validation tests, the log rates specification worked as well or better than the negative binomial specification.[3] For GBD 2015, the computational engine (DisMod-MR 2.1) remained substantively unchanged but we rewrote the “wrapper” code that organises the flow of data and settings at each level of the analytical cascade. The sequence of estimation occurs at five levels: global, super-region, region, country and, where applicable, subnational geographical units. The super-region priors are generated at the global level with mixed-effects, nonlinear regression using all available data; the super-region fit, in turn, informs the region fit, and so on down the cascade. The wrapper gives analysts the choice to branch the cascade in terms of time and sex at different levels depending on data density. The default used in most models is to branch by sex after the global fit but to retain all years of data until the lowest level in the cascade. For GBD 2015, we generated fits for the years 1990, 1995, 2000, 2005, 2010, and 2015; see Appendix Figure 2 below.

Appendix Figure 2. GBD 2015 DisMod-MR 2.1 Analytical Cascade

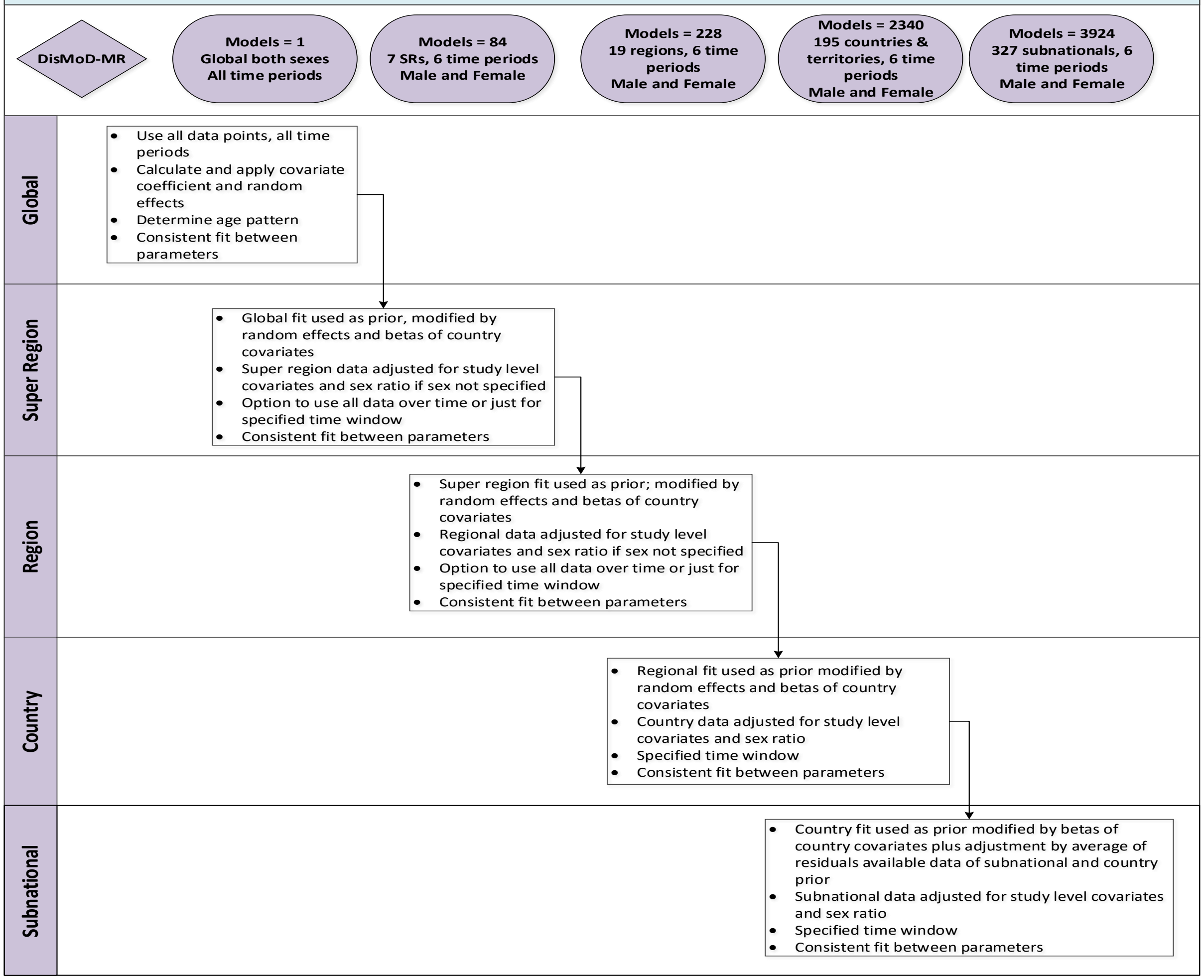

In updating the "wrapper," we consolidated the code base into a single language, Python, to make the code more transparent and efficient and to better deal with subnational estimation. The computational engine is limited to three levels of random effects; we differentiate estimates at the super-region, region and country level. In GBD 2013, the subnational units of China, the UK, and Mexico were treated as "countries" such that a random effect was estimated for every geography with contributing data. However, the lack of a hierarchy between country and subnational units meant that the fit to country data contributed as much to the estimation of a subnational unit as the fits for all other countries in the region. We found inconsistency between the country fit and the aggregation of subnational estimates when the country's epidemiology varied from the average of the region. Adding an additional level of random effects required a prohibitively comprehensive rewrite of the underlying DisMod-MR engine. Instead, we added a fifth layer to the cascade, with subnational estimation informed by the country fit and country covariates, plus an adjustment based on the average of the residuals between the subnational unit's available data and its prior. This mimicked the impact of a random effect on estimates between subnationals.

For GBD 2015 we improved how country covariates differentiate nonfatal estimates for diseases with sparse data. The coefficients for country covariates are re-estimated at each level of the cascade. For a given geography, country coefficients are calculated using both data and prior information available for that geography. In the absence of data, the coefficient of its parent geography is used, in order to utilise the predictive power of our covariates in data-sparse situations.

c. DisMod-MR 2.1 likelihood estimation

Analysts have the choice of using a Gaussian, log-Gaussian, Laplace, or Log-Laplace likelihood function in DisMod-MR 2.1 The default log-Gaussian equation for the data likelihood is:

$$-log[p(y_j|\Phi)] = \log(\sqrt{2\pi}) + \log(\delta_j + s_j) + \frac{1}{2}\left(\frac{\log(a_j + \eta_j) - \log(m_j + \eta_j)}{\delta_j + s_j}\right)^2$$

where, y_j is a "measurement value" (ie, data point); Φ denotes all model random variables; η_j is the offset value, eta, for a particular "integrand" (prevalence, incidence, remission, excess mortality rate, with-condition mortality rate, cause-specific mortality rate, relative risk, or standardised mortality ratio) and a_j is the adjusted measurement for data point j, defined by:

$$a_j = e^{(-u_j - c_j)} y_j$$

where u_j is the total "area effect" (ie, the sum of the random effects at three levels of the cascade: super-region, region, and country) and c_j is the total covariate effect (ie, the mean combined fixed effects for sex, study-level, and country-level covariates), defined by:

$$c_j = \sum_{k=0}^{K[I(j)]-1} \beta_{I(j),k} \hat{X}_{k,j}$$

with standard deviation

$$s_j = \sum_{l=0}^{L[I(j)]-1} \zeta_{I(j),l} \hat{Z}_{k,j}$$

where k denotes the mean value of each data point in relation to a covariate (also called x-covariate); I(j) denotes a data point for a particular integrand, j; $\beta_{I(j),k}$ is the multiplier of the kth x-covariate for the ith integrand; $\hat{X}_{k,j}$ is the covariate value corresponding to the data point j for covariate k; l denotes the standard deviation of each data point in relation to a covariate (also called z-covariate); $\zeta_{I(j),k}$ is the multiplier of the lth z-covariate for the ith integrand; and δ_j is the standard deviation for adjusted measurement j, defined by:

$$\delta_j = log[y_j + e^{(-u_j - c_j)}\eta_j + c_j] - log[y_j + e^{(-u_j - c_j)}\eta_j]$$

Where m_j denotes the model for the jth measurement, not counting effects or measurement noise and defined by:

$$m_j = \frac{1}{B(j) - A(j)} \int_{A(j)}^{B(j)} I_j(\mathrm{a})\,\mathrm{da}$$

where A(j) is the lower bound of the age range for a data point; B(j) is the upper bound of the age range for a data point; and I_j denotes the function of age corresponding to the integrand for data point j.

Spatiotemporal Gaussian process regression

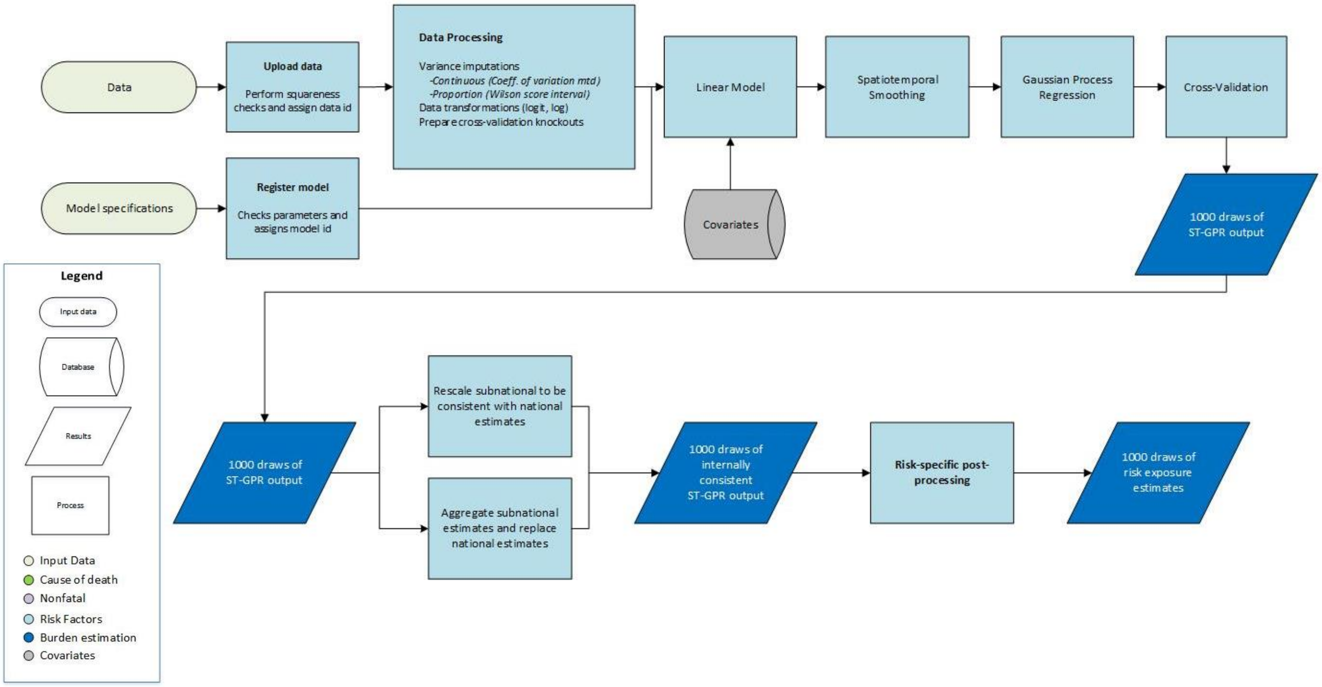

Spatiotemporal Gaussian process regression (ST-GPR) has been used for risk factors where the data density is sufficient to estimate a very flexible time trend. The approach is a stochastic modelling technique that is designed to detect signals amidst noisy data. It also serves as a powerful tool for interpolating non-linear trends.[1,2] Unlike classical linear models that assume that the trend underlying data follows a definitive functional form, GPR assumes that the specific trend of interest follows a Gaussian process, which is defined by a mean function $m(\cdot)$ and a covariance function $Cov(\cdot)$. For example, let $p_{c,a,s,t}$ be the exposure, in normal, log, or logit space, observed in country c, for age group a, and sex s at time t:

$$(p_{c,a,s,t}) = g_{c,a,s}(t) + \epsilon_{c,a,s,t}$$

where

$$\epsilon_{c,a,s,t} \sim Normal(0, \sigma_p^2),$$

$$g_{c,a,s}(t) \sim GP\left(m_{c,a,s}(t), Cov\left(g_{c,a,s}(t)\right)\right).$$

The derivation of the mean and covariance functions, $m_{c,a,s}(t)$ and $Cov\left(g_{c,a,s}(t)\right)$, along with a more detailed description of the error variance (σ_p^2), is described below.

Estimating mean functions

We estimated mean functions using a two-step approach. To be more specific, $m_{c,a,s}(t)$ can be expressed, depending on the exposure transformation, as:

$$log\big(p_{c,a,s}(t)\big) = X_{c,a,s}\beta + h(r_{c,a,s,t})$$

$$logit\big(p_{c,a,s}(t)\big) = X_{c,a,s}\beta + h(r_{c,a,s,t})$$

$$p_{c,a,s}(t) = X_{c,a,s}\beta + h(r_{c,a,s,t})$$

where $X\beta$ is the summation of the components of a hierarchical mixed-effects linear regression, including the intercept and the product of covariates with their corresponding fixed effect coefficients. For a majority of models, predictions were not made using the random effects component of the linear model. The second part of the equation, $h(r_{c,a,s,t})$, is a smoothing function for the residuals, $r_{c,a,s,t}$, derived from the linear model.[3] Descriptions of exposure transformations and which covariates were used in linear models can be found in Section 3, Risk-specific estimation.

While the linear component captures the general trend in exposures over time, much of the data varaibility may still not be adequately accounted for. To address this, we fit a locally weighted polynomial regression (LOESS) function $h(r_{c,a,s,t})$ to systematically estimate this residual variability by borrowing strength across time, age, and space patterns (the spatiotemporal component of ST-GPR). The time adjustment parameter, defined by λ , aims to borrow strength from neighbouring time points (ie, the exposure in this year is highly correlated with exposure in the previous year but less so further back in time). The age adjustment parameter, defined by ω, borrows strength from data in neighbouring age groups. The space adjustment parameter, defined by ξ, aims to borrow strength across the hierarchy of geographical locations.

Let $w_{c,a,s,t}$ be the final weight assigned to observation $r_{c,a,s,t}$ with reference to a focal observation r_{c_0,a_0,s_0,t_0}. We first generated a preliminary weight $w'_{c,a,s,t}$ for smoothing over time, which was based on the scaled distance along the time dimension of the two observations:

$$w'_{c,a,s,t} = \left(1 - \left(\frac{|t - t_0|}{1 + \max|t - t_0|}\right)^{\lambda}\right)^3$$

Next, we calculated the weight $w''_{c,a,s,t}$ to smooth over age, which is based on a distance along the age dimension of two observations. For a point between the age a of the observation $r_{c,a,s,t}$ and a focal observation r_{c_0,a_0,s_0,t_0}, the weight is defined as follows:

$$w''_{c,a,s,t} = \frac{1}{e^{\omega|a-a_0|}}$$

Finally, these combined weights were multiplied and further adjusted to account for geographic patterns.

Specifically, we defined a geospatial relationship by categorizing data based on the GBD location hierarchy. We adapted the weighting strategy used in previous studies estimating time series of global indicators to be more flexible with respect to estimating subnational locations and to borrow strength from all levels.[3,4] A vector of spatial weights corresponding to each level of the location hierarchy was derived as $[\xi, \xi * (1-\xi)^{n_1 - 1}, \ldots, \xi * (1-\xi)^{n_i - 1}, (1-\xi)^{n_i}]$, where the vector is expanded to include the number, n_i, levels in the location hierarchy between the location being estimated and global, which

recieves a pre-rescaling weight of $(1-\xi)^{n_i}$. For example, estimating a country would use the following weighting scheme:

- Country data: ξ
- Regional data not from the country being estimated: $\xi * (1- \xi)$
- Data from other regions in the same super-region: $\xi * (1-\xi)^2$
- Global data from other super-regions: $(1-\xi)^3$

A full derivation of weights for each category follow, assuming the location being estimated was a country, follows:

1) If the observation $r_{c,t}$ belongs to the same country c_0 of the focal observation r_{c_0,t_0}:

$$w_{c,a,s,t} = \frac{\xi(w'_{c,a,s,t}w''_{c,a,s,t})}{\sum_{c=c_0}(w'_{c,a,s,t}w''_{c,a,s,t})} \qquad \forall c = c_0$$

2) If the observation $r_{c,t}$ belongs to a different country than the focal observation r_{c_0,t_0}, but both belong to the same region R:

$$w_{c,a,s,t} = \frac{\xi * (1- \xi)\ (w'_{c,a,s,t}w''_{c,a,s,t})}{\sum_{c\neq c_0}(w'_{c,a,s,t}w''_{c,a,s,t})} \qquad \forall c \neq c_0 \cap\ R[c] = R[c_0]$$

3) If the observation $r_{c,t}$ belongs to the same super-region SR but to a both different country c_0 and region $R[c_0]$ than the focal observation r_{c_0,t_0}:

$$w_{c,a,s,t} = \frac{\xi * (1- \xi)^2\ (w'_{c,a,s,t}w''_{c,a,s,t})}{\sum_{c\neq c_0}(w'_{c,a,s,t}w''_{c,a,s,t})} \qquad \forall c \neq c_0 \cap\ R[c] \neq R[c_0] \cap\ SR[c] = SR[c_0]$$

4) If the observation $r_{c,t}$ is from a different super-region than the focal observation r_{c_0,t_0}(ie, all other data currently not receiving a weight):

$$w_{c,a,s,t} = \frac{(1- \xi)^3\ (w'_{c,a,s,t}w''_{c,a,s,t})}{\sum_{c\neq c_0}(w'_{c,a,s,t}w''_{c,a,s,t})} \qquad \forall c \neq c_0 \cap\ R[c] \neq R[c_0] \cap\ SR[c] \neq SR[c_0]$$

To allow additional flexibility and specificity in weighting schemes, we allowed for two different ξ to be defined. The higher ξ was applied when at least one age-sex group in the country of estimation had at least five unique data points. The lower ξ was applied when estimating data-scarce countries.

Observations could be downweighted by a factor of 0.1, usually because they were not geographically representative at the unit of estimation. Details of reasons for downweighting can be found in risk-specific modelling summaries. The final weights were then normalised such that the sum of weights across age, time, and geographic hierarchy for a reference group was 1.

Estimating error variance

σ_p^2 represents the error variance in normal or transformed space including sampling variance of the estimates and predication error from any crosswalks performed. First, variance was systematically imputed if the data extraction did not include any measure of uncertainty. When some sample sizes for

data were available, missing sample sizes were imputed as the 5th percentile of available sample sizes. Missing variances were then calculated as $\sigma_p^2 = \frac{p*(1-p)}{n}$ for proportions and using the global coefficient of variation for continuous exposures. When sample sizes were entirely missing and could not be imputed, the 95th percentile of available variances at the most granular geographic level (ie, first country, then region, etc.) were used to impute missing variances. For proportions where *p*n* or *(1-p)*n* is < 20, variance was replaced using the Wilson Interval Score method.

Next, if the exposure was modelled as a log transformation, the error variance was transformed into log-space using the delta method approximation as follows,

$$\sigma_p^2 \cong \frac{\sigma_{p\prime}^2}{p_{c,a,s,t}^2}$$

where $\sigma_{p\prime}^2$, represents the error variance in normal space. If the exposure was modelled as a logit transformation, the error variance was transformed into logit-space using the delta method approximation as follows,

$$\sigma_p^2 \cong \frac{\sigma_{p\prime}^2}{(p_{c,a,s,t} * (1 - p_{c,a,s,t}))^2}$$

Finally, prior to GPR, an approximation of non-sampling variance was added to the error variance. Calculations of non-sampling variance were performed on normal-space variances, and before GPR variances were again transformed using the delta method approximation, if necessary. Non-sampling variance was calculated as the variance of inverse-variance weighted residuals from ST at a given location level hierarchy. If there were fewer than five data points at a given level of the location hierarchy the non-sampling variance was replaced with that of the next-highest geography level with more than five data points.

Estimating the covariance function

The final input into GPR is the covariance function, which defines the shape and distribution of the trends. Here, we have chosen the Matern-Euclidian covariance function, which offers the flexibility to model a wide spectrum of trends with varying degrees of smoothness. The function is defined as follows:

$$M(t,t') = \sigma^2 \frac{2^{1-v}}{\Gamma(\mathrm{v})} \left(\frac{d(t,t')\sqrt{2v}}{l}\right)^v K_v \left(\frac{d(t,t')\sqrt{2v}}{l}\right)$$

where $d(\cdot)$is a distance function; σ^2, v, l, and K_v are hyperparameters of the covariance function – specifically σ^2 is the marginal variance, v is the smoothness parameter that defines the differentiability of the function, l is the length scale, which roughly defines the distance between which two points become uncorrelated, and K_v is the Bessel function. Based on previous applications of ST-GPR, we approximated σ^2 by $MADN(r'_{c,t})$, which is the normalised absolute deviation of the residuals from the smoothing step for each country, region, or super-region depending on the data coverage at a given location hierarchy level. Here, we have used the parameter specifications $v = 2$ and $l = 20$.

Prediction using GPR

Based on the specifications stated above, we integrated over $g_{c,t}(t_*)$ to predict the full time series of mean SBP for country c, age a, sex s, and the prediction time t_*:

$$log(p_{c,a,s}(t_*)) \sim N\left(m_{c,a,s,t}(t_*), \sigma_p^2 I + Cov\left(g_{c,a,s,t}(t_*)\right)\right)$$

Random draws of 1,000 samples were obtained from the distributions above for every country for a given indicator. The final estimated mean for each country was the mean of the draws. In addition, 95% uncertainty intervals were calculated by taking the 2.5th and 97.5th percentile of the sample distribution. The entire modelling process was performed in log space and back-transformed to obtain final estimates in the original scale. The linear modelling process was implemented using the lmer4 package in R, and the ST-GPR analysis was implemented through the PyMC2 package in Python.

Subnational scaling and aggregation

To ensure consistency of the estimates between countries and their respective subnational locations, national estimates were either created by population-weighted aggregation or subnational estimates were adjusted by population-weighted scaling to the national estimates, depending on the data coverage of a given country compared to that of its subnational locations. For example, if there was better data coverage at the national level, relative to its corresponding subnational locations, for a given country and risk across age, sex, and time, estimates were raked to the national level. Conversely, if there was better data coverage at the subnational level, estimates for its parent country were created through population-weighted aggregation.

References

1) Rasmussen CE, Williams CKI. *Gaussian Processes for Machine Learning*. MIT Press, 2006.
2) Vasudevan S, Ramos F, Nettleton E, Durrant-Whyte H. Gaussian process modeling of large-scale terrain. *J Field Robot* 2009; **26**: 812–40.
3) Ng M, Freeman MK, Fleming TD, *et al.* Smoking prevalence and cigarette consumption in 187 countries, 1980-2012. *JAMA* 2014; **311**: 183–92.
4) Ng M, Fleming T, Robinson M, *et al.* Global, regional, and national prevalence of overweight and obesity in children and adults during 1980-2013: a systematic analysis for the Global Burden of Disease Study 2013. *Lancet Lond Engl* 2014; **384**: 766–81.

Fatal chronic respiratory diseases estimation process

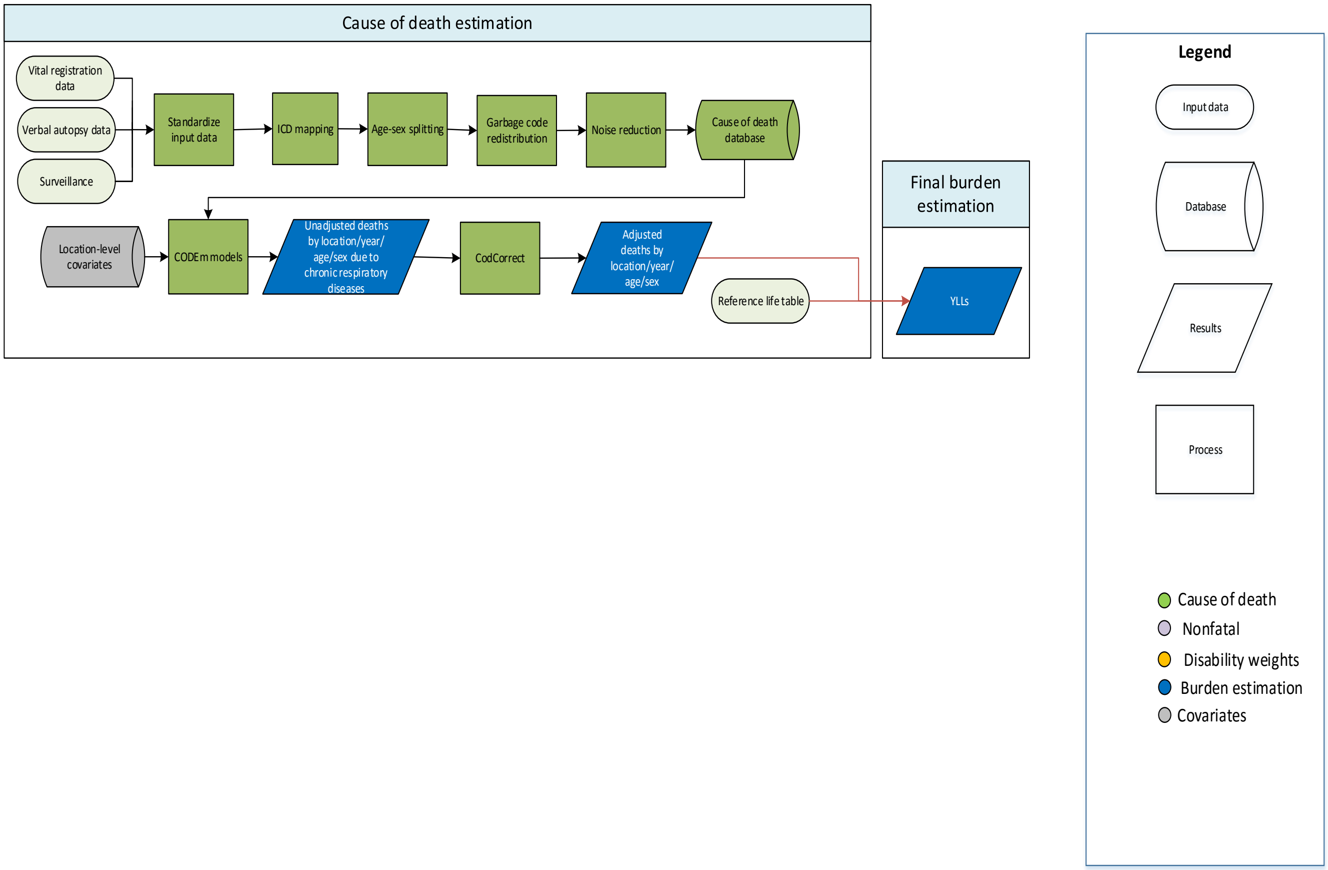

Input data

Sources used to estimate chronic respiratory disease mortality included vital registration, verbal autopsy, and surveillance data from the cause of death (COD) database. Our outlier criteria excluded data points that (1) were implausibly high or low, (2) substantially conflicted with established age or temporal patterns, or (3) significantly conflicted with other data sources conducted from the same locations or locations with similar characteristics (ie, Socio-demographic Index).

For GBD 2015, there were two significant changes in the data preparation process that affect chronic respiratory diseases and its children causes. First, the algorithm package that redistributes heart-failure-related garbage codes has been updated to take into account the "side" of the heart failure – with right heart failure denoting an underlying respiratory disease. Second, verbal autopsy data are no longer used to inform children causes as they are thought to be unreliable below this cause level. Practically, this has a larger influence on the uncorrected children models than the parent Chronic Respiratory Diseases model discussed here.

Modelling strategy

The standard CODEm modelling approach was applied to estimate deaths due to chronic respiratory diseases. Chronic respiratory diseases served as the parent cause to chronic obstructive pulmonary disease, pneumoconiosis (including silicosis, asbestosis, coal worker's pneumoconiosis, other pneumoconiosis), asthma, interstitial lung disease and pulmonary sarcoidosis, and other chronic respiratory diseases. Functionally, this means the death estimates for chronic respiratory diseases serve as an envelope into which the children causes are squeezed by the CodCorrect algorithm. This approach allows us to use a broader range of data – specifically verbal autopsy data – which cannot be accurately mapped to a cause further down in the hierarchy.

Separate models were conducted for male and female mortality, and the age range for both models was 0 to 80+ years. The same covariates from GBD 2013 were used, with the addition of the Socio-demographic Index (SDI) covariate. Although all covariates in this model received updates for GBD 2015, cumulative cigarettes, smoking prevalence, and health systems access received the larger overhauls. The updates to the smoking-based covariates were particularly helpful in developing these models. Beyond changes in the underlying covariates, there were no substantial deviations from the GBD 2013 approach.

Fatal chronic obstructive pulmonary disease estimation process

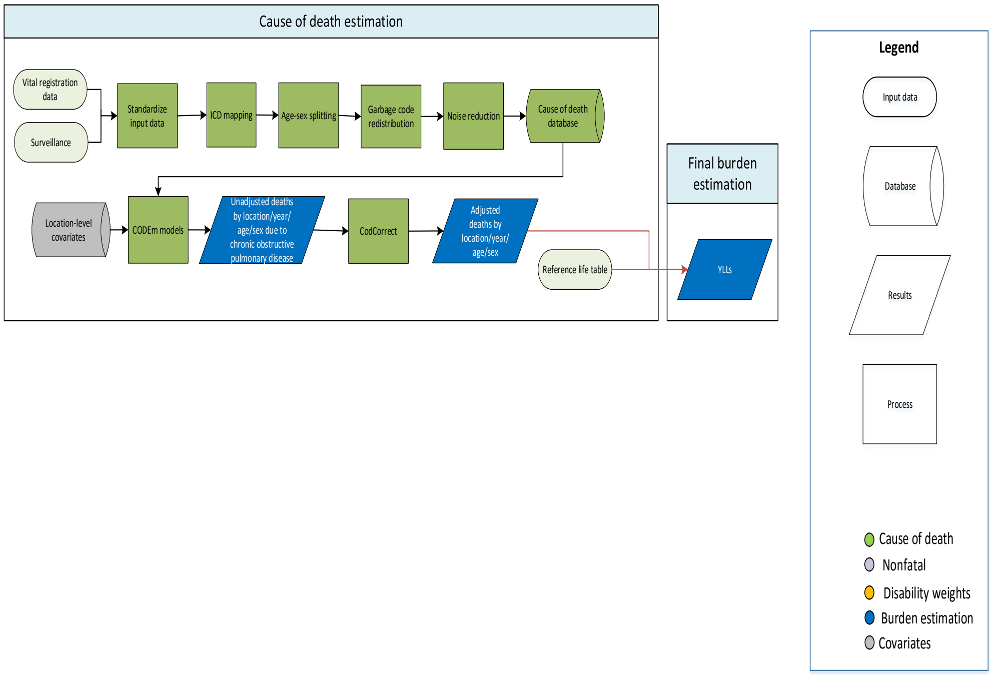

Input data

Data used to estimate chronic obstructive pulmonary disease (COPD) mortality included vital registration and surveillance data from the cause of death (COD) database. Our outlier criteria excluded data points that (1) were implausibly high or low, (2) substantially conflicted with established age or temporal patterns, or (3) significantly conflicted with other data sources conducted from the same locations or locations with similar characteristics (ie, Socio-demographic Index). The main consequences of this protocol are the mapping of state-level data from India MCCD ICD10 to the chronic respiratory parent due to implausibly high values and the outliering of some Thailand vital registration data from the late 1990s that implied an unreasonable peak of COPD during the covered time frame.

Notable differences in the data processing strategy relative to GBD 2013 include the following: 1) Verbal autopsy data have been excluded from this model and mapped to the chronic respiratory disease parent cause as we no longer believe that verbal autopsy accurately captures deaths due to specific respiratory diseases, and 2) the heart failure redistribution package has been updated to account for the “side” of the heart. As a result, the amount of heart failure being attributed to chronic respiratory diseases is now largely based on proportions of left and right heart failure (with right heart failure signifying an underlying respiratory condition). In general, this has reduced the level of COPD deaths – all else being equal.

Modelling strategy

The standard CODEm modelling approach was applied to estimate deaths due to COPD. Separate models were conducted for male and female mortality, and the age range for both models was 28 days to 80+ years. The mortality estimates from the COPD models were ultimately fit into the chronic respiratory diseases envelope.

While the core covariates have remained unchanged, covariates relating to population density and proportion of population living between 500 meters and 1,500 meters of elevation have been removed because they increased model run time without substantially contributing to the model results. Conversely, 10-year cumulative cigarette consumption has been added to the model to better capture any smoking-related lag effects on COPD, along with the Socio-demographic Index (SDI) covariate.

Fatal asthma estimation process

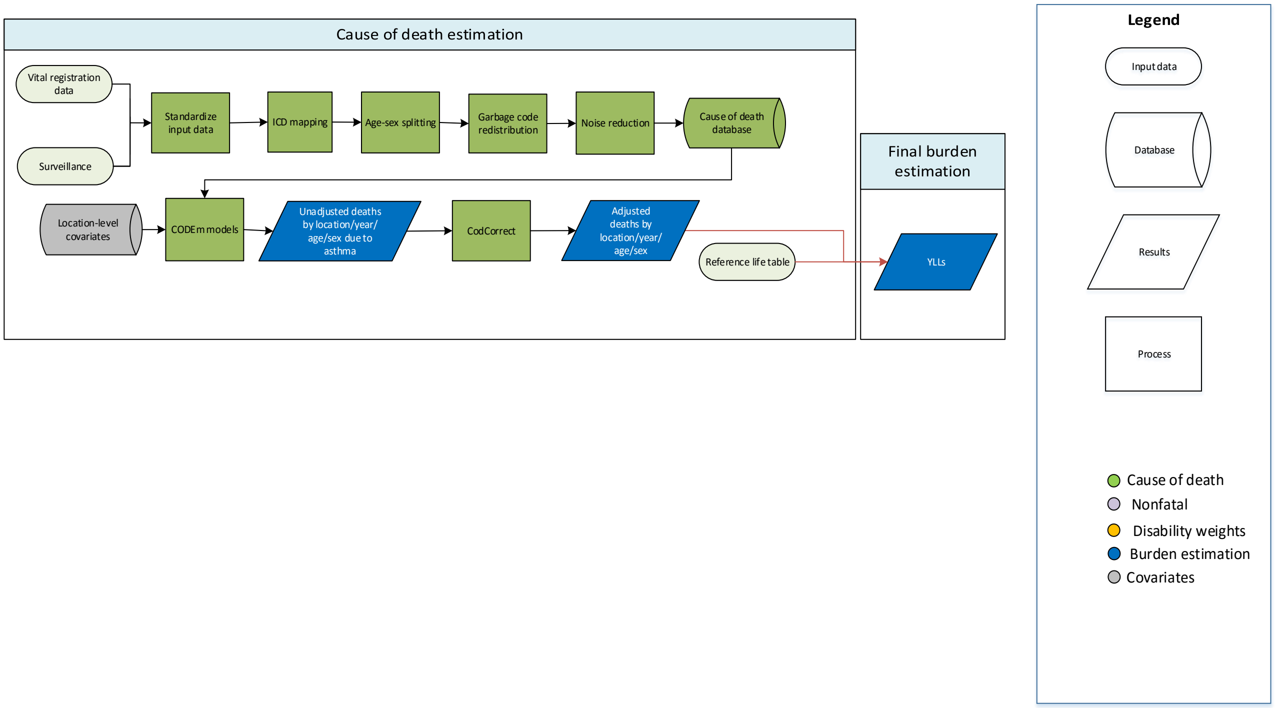

Input data

Data used to estimate asthma mortality included vital registration and surveillance data from the cause of death (COD) database. Verbal autopsy data were not included and were instead mapped to the parent model (chronic respiratory diseases). Our outlier criteria excluded data points that (1) were implausibly high or low relative to global or regional patterns, (2) substantially conflicted with established age or temporal patterns, or (3) significantly conflicted with other data sources conducted from the same locations or locations with similar characteristics (ie, Socio-demographic Index).

Modelling strategy

The standard CODEm modelling approach was applied to estimate deaths due to asthma. Separate models were conducted for male and female mortality, and the age range for both models was 1–80+ years. The mortality estimates from the asthma models were ultimately fit into the chronic respiratory diseases envelope.

Notable differences between the GBD 2013 strategy and this iteration are across-the-board updates in smoking-based covariates, the removal of elevation and population density covariates due to lack of informative contributions, and the inclusion of the Socio-demographic Index (SDI) covariate and the SEV-scalar (disease-specific values that reflect the combined effect of all GBD risks) for asthma.

Non-fatal chronic obstructive pulmonary disease (COPD) estimation process

Chronic Obstructive Pulmonary Disease (COPD)

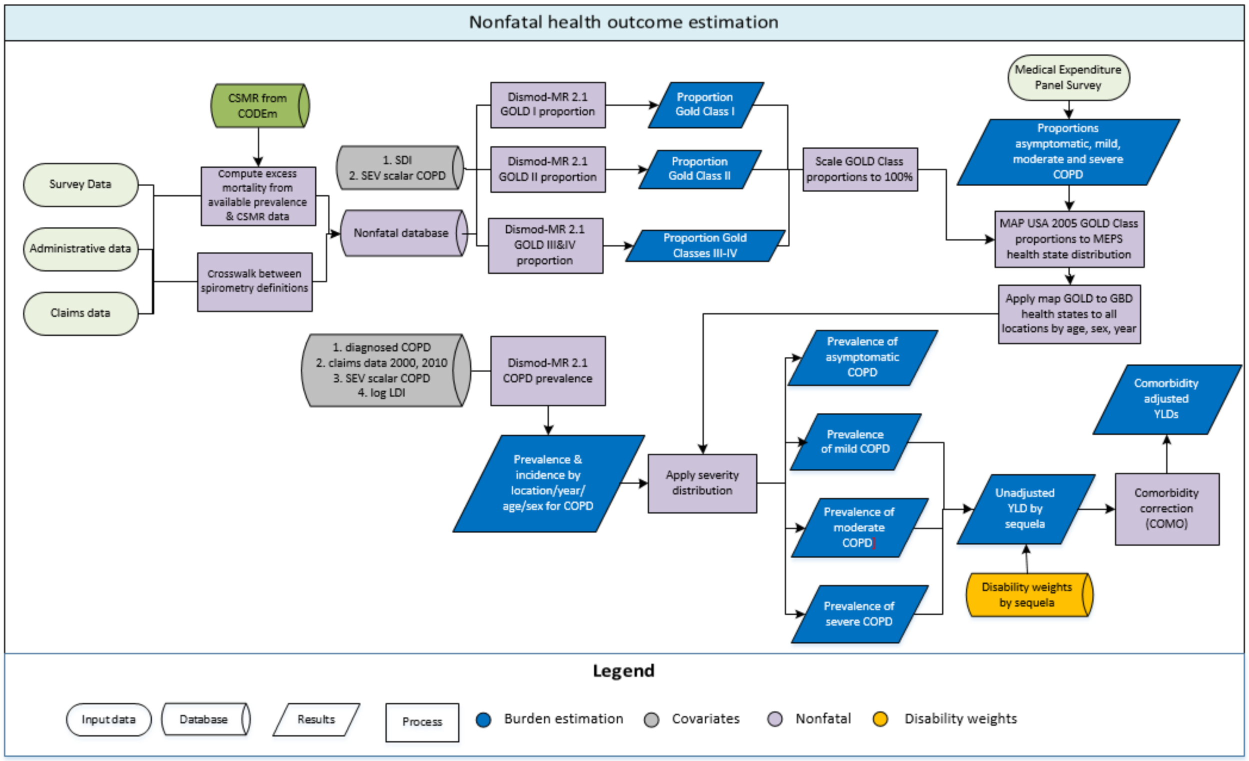

Input data and methodological summary

Case definition

COPD is defined as in the GOLD classification: a measurement of <0.7 FEV_1/FVC (1 second of forceful exhalation/total forced expiration) on spirometry after bronchodilation. It should be noted that this a change from GBD 2013 where the "Lower Limit of Normal (LLN)," ie, relative to an age- and sex-specific norm for the FEV_1/FVC ratio, was the reference. We made this decision because the severity grading of COPD follows the GOLD class definition rather than the LLN concept. The definitions of the severity classes in the GOLD classification are provided below.

GOLD class	FEV_1 Score
I: Mild	>=80% of normal
II: Moderate	50-79% of normal
IV: Severe	<50% of normal

ICD-10 codes associated with COPD include J40, J41, J42, J43, J44, and J47. The corresponding ICD-9 codes are 490–492, 494, and 496.

Input data

For GBD 2015, we updated the systematic review from previous iterations. The full search term was:

(chronic obstructive pulmonary disease[Title/Abstract] AND (prevalence[Title/Abstract] or incidence [Title/Abstract] or mortality [Title/Abstract] or death [Title/Abstract]) Filters: Publication date from 2013/01/01 to 2015/12/31; Humans

The search period was between 1/1/2013 and 5/13/2015. Twenty-one new sources were extracted. Studies excluding smokers were excluded from the review.

In addition, we searched for survey data with spirometry measurements in GHDx, GBD's health data repository. We systematically extracted all spirometry data from the National Health and Nutrition Examination Study series in the United States for which we had only used some published studies in previous GBD studies. The Study of Aging and Global Health (SAGE) series was also examined but ultimately excluded as the spirometry data had implausible FEV_1/FVC values (eg, over 1).

Furthermore, claims data for the United States were included. Additional information on the claims data collection and pre-corrections are provided elsewhere. Briefly, we determined US national and state-level estimates of COPD prevalence from a database of individual-level ICD-coded health service encounters. Persons with any claim associated with COPD were marked as a prevalent case for that year.

Studies that provided non-standard cutoffs of COPD prevalence (eg, not 0.7) were crosswalked before the main analysis step to match the 0.7 FEV_1/FVC case definition.

A table describing the density and distribution of the available data informing the COPD estimation process is provided below.

	Proportion by GOLD class	Prevalence	Incidence
Studies	15	73	5
Countries or subnational locations	28	116	5
Regions	15	15	4

COPD prevalence input data, males, 2015

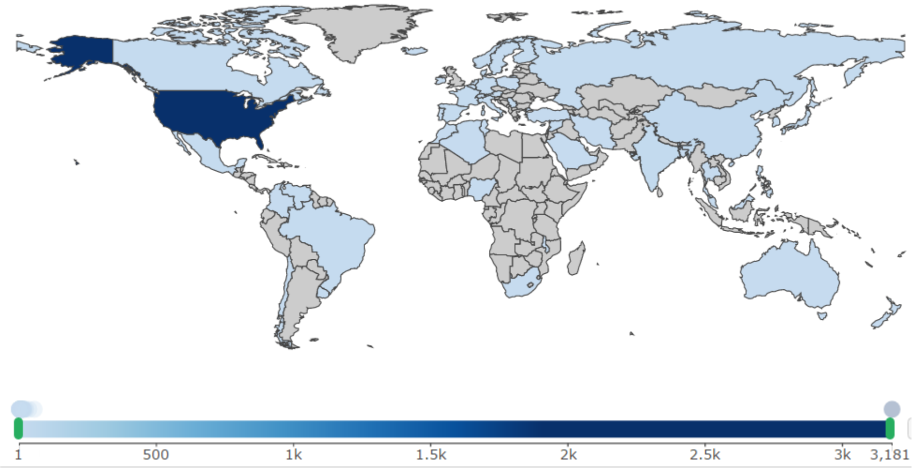

COPD prevalence input data, subnational view, males, 2015

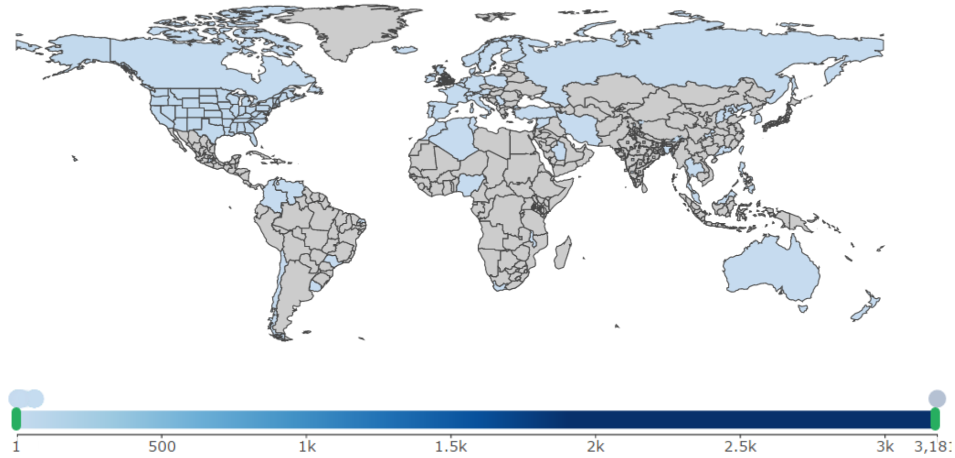

COPD GOLD class input data, 2015

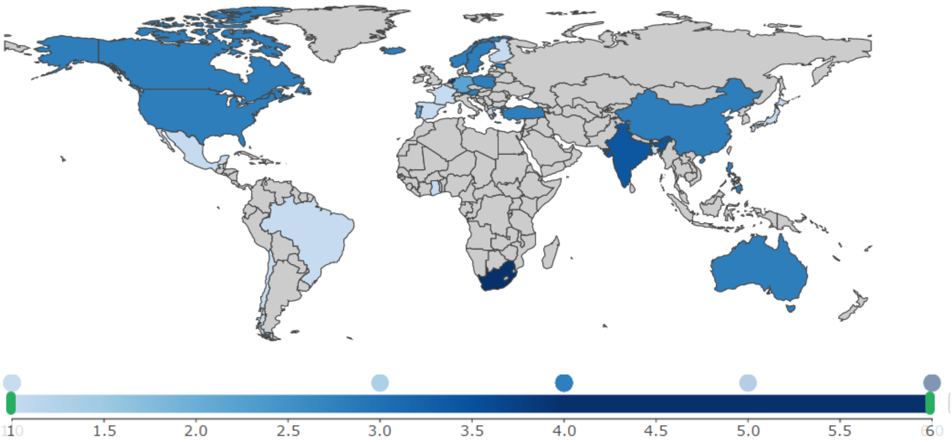

Modelling strategy

As described above, the estimation of COPD burden occurs in three main steps. The first is the estimation of prevalence and incidence using a DisMoD-MR 2.1 model. The second is the separate estimation of the proportions by three GOLD class groupings in DisMoD-MR 2.1. The third is the combination of these two processes to derive prevalence by severity.

Step 1: Main COPD model

Prior settings include remission of 0 and an incidence ceiling of 0.001 before age 20. The latter was necessary to avoid a kick-up of estimates in childhood at an age range with few or no primary data.

Claims data for 2000 and 2010 were adjusted via study covariates to account for systematically low estimates relative to the 2012 claims data. Implicit in this adjustment is the assumption that variation between years of claims data is a function of data collection inconsistencies.

Similar to other causes, we include estimates of cause-specific mortality rate (CSMR) and derived estimates of excess mortality rate (EMR) by dividing every prevalence data point by the CSMR value for the corresponding location, age, and sex-year. We did not estimate EMR for data points with an age range greater than 20 years.

To assist estimation, each model includes a series of country-level covariates that describe spatiotemporal patterns. Where available, we use the COPD standardised exposure variable (SEV), which aggregates multiple risk factors into a single variable. We also use the log of LDI on EMR to capture country-level variation of EMR, assuming a negative coefficient (ie, lower mortality with rising GDP).

Step 2: GOLD class models

The GOLD class models use data from surveys that specified prevalence by GOLD class after expressing the values as a proportion of all COPD cases. We use fixed effects from the SEV scalar and the log of LDI per capita to assist estimation.

Table of model coefficients for COPD and GOLD class models

Cause	Variable name	Measure	Beta	Exponentiated
COPD	LDI (I$ per capita)	excess mortality rate	-0.5	0.61 (0.61–0 .61)
COPD	Log age-standardised SEV scalar: COPD	prevalence	0.76	2.13 (2.12–2.16)
COPD	Claims data 2010	prevalence	-0.071	0.93 (0.90–0.96)
COPD	Claims data 2000	prevalence	-0.12	0.89 (0.86–0.92)
COPD	Diagnosed COPD	prevalence	0.13	1.14 (1.02–1.31)
GOLD I proportion	Socio-demographic Index	proportion	0.89	2.44 (0.35–7.00)
GOLD I proportion	Log age-standardised SEV scalar: COPD	proportion	-0.18	0.8349 (0.53–1.29)

GOLD II proportion	Socio-demographic Index	proportion	-0.50	0.6062 (0.16–2.38)
GOLD II proportion	Log age-standardised SEV scalar: COPD	proportion	-0.09	0.91 (0.59–1.6)
GOLD III+IV proportion	Socio-demographic Index	proportion	-0.65	0.5247 (0.15–3.9)
GOLD III+IV proportion	Log age-standardised SEV scalar: COPD	proportion	0.001	1.001 (-0.45–2.16)

Severity

The three GOLD class groupings reflect a grading based on a physiological measurement rather than a direct measurement of disease severity. In order to map the epidemiological findings by GOLD class into the three COPD health states for which we have disability weights (DW), we used the 2001–2011 Medical Expenditure Panel Survey (MEPS) data from the United States. Specifically, we convert the GOLD class designations estimated for the USA in 2005 (the midpoint of MEPS years of analyses) into GBD classifications of asymptomatic, mild, moderate, and severe COPD.

The table below shows the three health states of COPD and the corresponding lay descriptions and disability weights. The graph shows the average proportion by GOLD Class (after scaling to 100%) across all ages for USA in 2005. We also show the proportion of MEPS respondents reporting any health service contact in the past year for COPD with a DW value attributable to COPD of 0, mild range (0 to midpoint between DWs for mild and moderate), moderate range (midpoint of DW values mild and moderate to midpoint of DW values for moderate and severe), and severe range (midpoint between DW values moderate and severe or higher). The DW value for COPD was derived from a regression with indicator variables for all health states reported by MEPS respondents and their reported overall level of disability derived from a conversion of SF-12 answers to GBD DW values. This analysis gave the severity distribution for each GBD cause reported in MEPS after correcting for any comorbid causes individual respondents reported during a year.

Health state	Lay description	DW (95% CI)
Mild COPD	This person has cough and shortness of breath after heavy physical activity, but is able to walk long distances and climb stairs.	0.019 (0.011–0.033)
Moderate COPD	This person has cough, wheezing, and shortness of breath, even after light physical activity. The person feels tired and can walk only short distances or climb only a few stairs.	0.225 (0.153–0.31)
Severe COPD	This person has cough, wheezing, and shortness of breath all the time. The person has great difficulty walking even short distances or climbing any stairs, feels tired when at rest, and is anxious.	0.408 (0.273–0.556)

Non-fatal asthma estimation process

Asthma

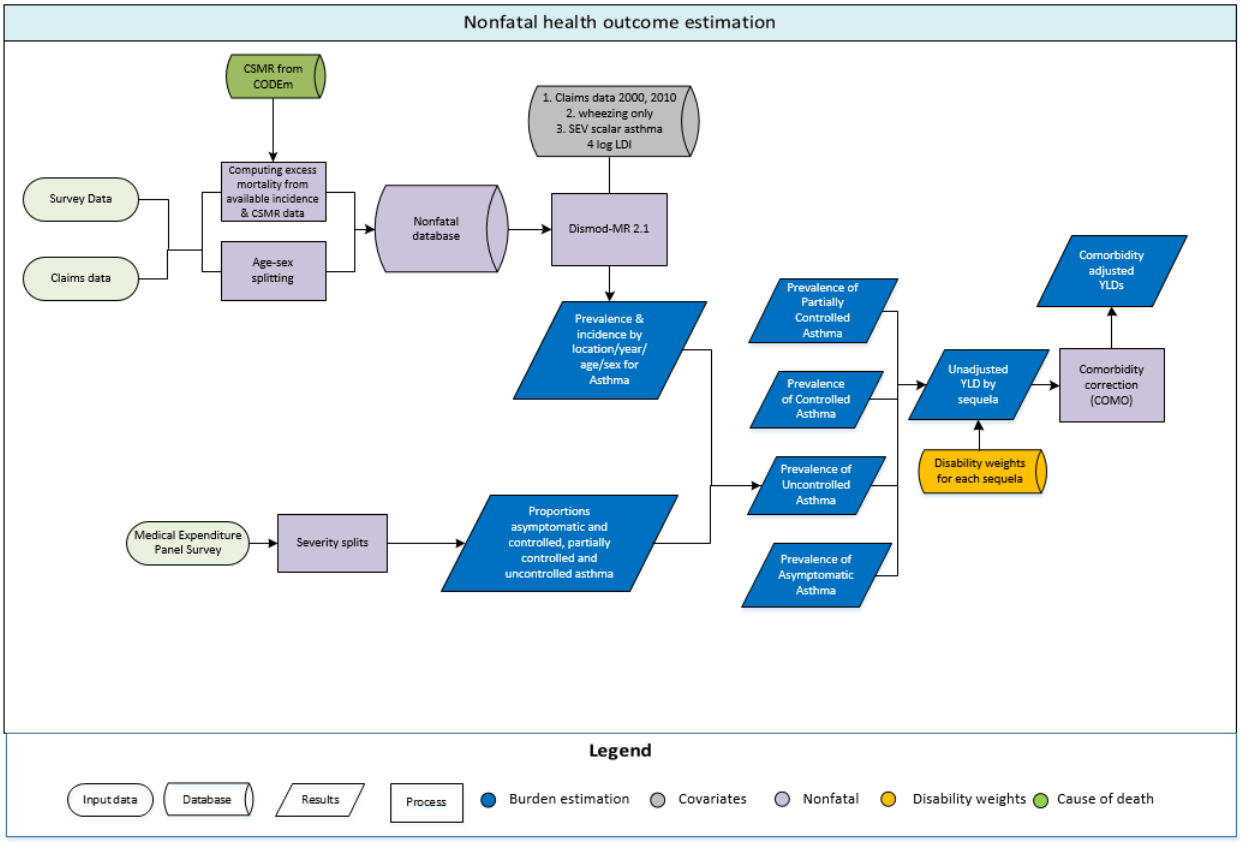

Case definition

Asthma is a chronic lung disease marked by spasms in the bronchi usually resulting from an allergic reaction or hypersensitivity and causing difficulty in breathing. We define asthma as a doctor's diagnosis and wheezing in the past year. The relevant ICD-10 codes are J45 and J46. ICD-9 code is 493.

Input data

Model inputs

For GBD 2015, we did not undertake a full systematic review of the literature on asthma. Instead, certain studies were re-extracted to ensure accuracy and several survey series for which we have individual records in our GHDx repository were added to the dataset. Data additions and re-analysis include the WHO Study on Global Aging and Adult Health series, the WHO World Health Survey series, and the Belgian Health Interview Survey. Surveys carried out as part of the International Study of Asthma and Allergies in Childhood (ISAAC) collaboration are the most important source of prevalence data in children.

The following table provides a description of the data density and distribution by geography and epidemiological measure (including the claims data discussed below).

	Prevalence	Incidence	Mortality risk
Studies	267	7	7
Countries/subnational locations	248	5	3
Regions	21	1	1

In addition to literature and survey data, we use claims data from the United States from 2000, 2010, and 2012. Information on the source and preparation of these data are provided in detail elsewhere. Briefly, we determined US national and state-level estimates of asthma prevalence from a database of individual-level ICD-coded health service encounters for three years. Persons with any claim associated with asthma were marked as a prevalent case for that year. Aggregated estimates were then adjusted using a noise-reduction algorithm. These corrected data were then used in the modelling process.

Asthma prevalence input data, males, 2015

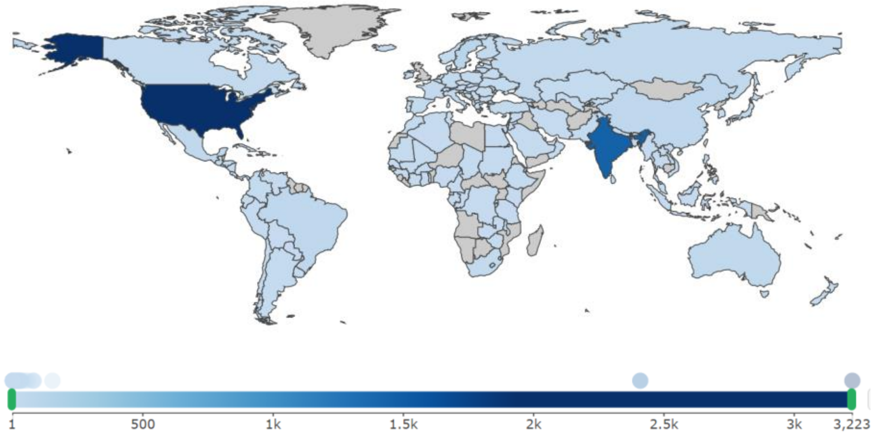

Asthma prevalence input data, subnational view, males, 2015

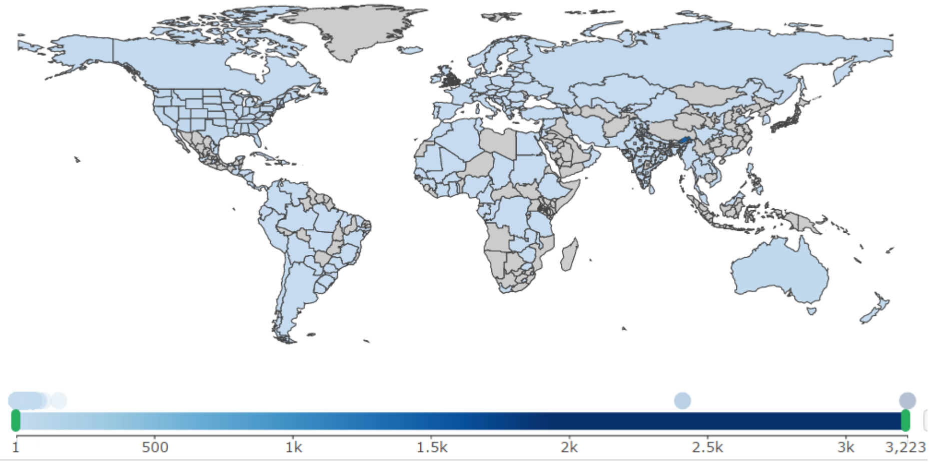

Modelling strategy

We use DisMod MR 2.1 as the main modelling tool for asthma. Prior settings include a maximum remission of 0.3 (reflecting the upper bound of the highest observed data) and no incidence between the ages of 0 and 0.5 year as a diagnosis cannot be made in young infants.

Data points from the ISAAC studies were reported for both sexes combined. We sex-split before modelling using the ratios derived from the 2012 US claims data (1).

Data that describe wheezing but do not report presence/absence of an accompanying diagnosis in the last year were crosswalked to the reference category. As the table below shows, studies that only report wheezing are systematically higher than reference data points and are adjusted down – dividing by the exponentiated coefficient).

To account for country-level differences in excess mortality as a function of available medical care we use log LDI as a covariate and assume a negative coefficient. The effect size is shown below.

Claims data for 2000 and 2010 are adjusted via study covariates to account for systematically lower estimates relative to the 2012 claims data. Implicit in this adjustment is the assumption that variation between years of claims data is a function of data collection inconsistencies.

Similar to other causes, we include estimates of cause-specific mortality rate (CSMR) and excess mortality rate (EMR) derived as a matched value for each prevalence data point dividing CSMR by prevalence. We restrict these EMR calculations to data points of 20-year age span or less.

To assist estimation, the model includes a series of country-level covariates that describe spatiotemporal patterns. Specifically, we use log LDI and the asthma standardised exposure variable (SEV), a scalar that combines exposure of all GBD risks that influence asthma. A full covariate list, including the study-level covariates described above, is presented in the following table with their associated effects:

Variable name	Measure	Beta	Exponentiated
Wheezing	prevalence	0.13	1.14 (1.08–1.22)
Claims data 2000	prevalence	-0.51	0.60 (0.59–0.61)
Claims data 2010	prevalence	-0.08	0.92 (0.91–0.94)
Log LDI (I$ per capita)	prevalence	-0.004	0.9938 (0.9737–1.007)
Log SEV scalar: asthma	prevalence	1.202	3.33 (2.97–3.49)
Log LDI (I$ per capita)	excess mortality rate	-0.42	0.65 (0.64–0 .67)

Severity split inputs

Lay descriptions and disability weights for the asthma health states are shown in table below. The distribution between the three health states is derived from an analysis of the US Medical Expenditure

Panel Surveys (MEPS). Briefly, MEPS is an ongoing survey of health service encounters with the main objective to collect data on health expenditure. Panels are recruited every year and followed up for a period of two years. Diagnostic information provided by respondents on the reasons for any health care contact is coded into three-digit ICD-9 codes by professional coders.

Twice over the two-year follow-up period respondents are asked to fill in SF-12. From convenience samples asking respondents to fill in SF-12 for 60 of the GBD health states, IHME has created a mapping from SF-12 scores to GBD disability weights (DW). We perform a regression with indicator variables for all GBD causes that we can identify from the ICD codes in MEPS to derive for each individual with a diagnosis the amount of disability that can be attributed to that condition after controlling for any comorbid conditions. Anyone with a diagnosis of asthma in whom the disability assigned to asthma is negative or zero we assume is asymptomatic (at the time of asking SF-12 question relating to their health status in the past four weeks). Non-zero values we bin into the three health states assuming a split between these at the midpoint between DW values. The table below gives the proportions in MEPS in each of the health states and an asymptomatic state.

Severity level	Lay description	DW (95% CI)	Severity distribution
Asymptomatic			30.2% (29.2–31.3)
Controlled	This person has wheezing and cough once a month, which does not cause difficulty with daily activities.	0.015 (0.007–0.026)	20.5% (13.7–28.6)
Partially controlled	This person has wheezing and cough once a week, which causes some difficulty with daily activities.	0.036 (0.022–0.055)	22.3% (16.6–28.1)
Uncontrolled	This person has wheezing, cough, and shortness of breath more than twice a week, which causes difficulty with daily activities and sometimes wakes the person at night.	0.133 (0.086–0.192)	26.9% (21.4–35.1)

Smoking risk factor estimation process

Smoking

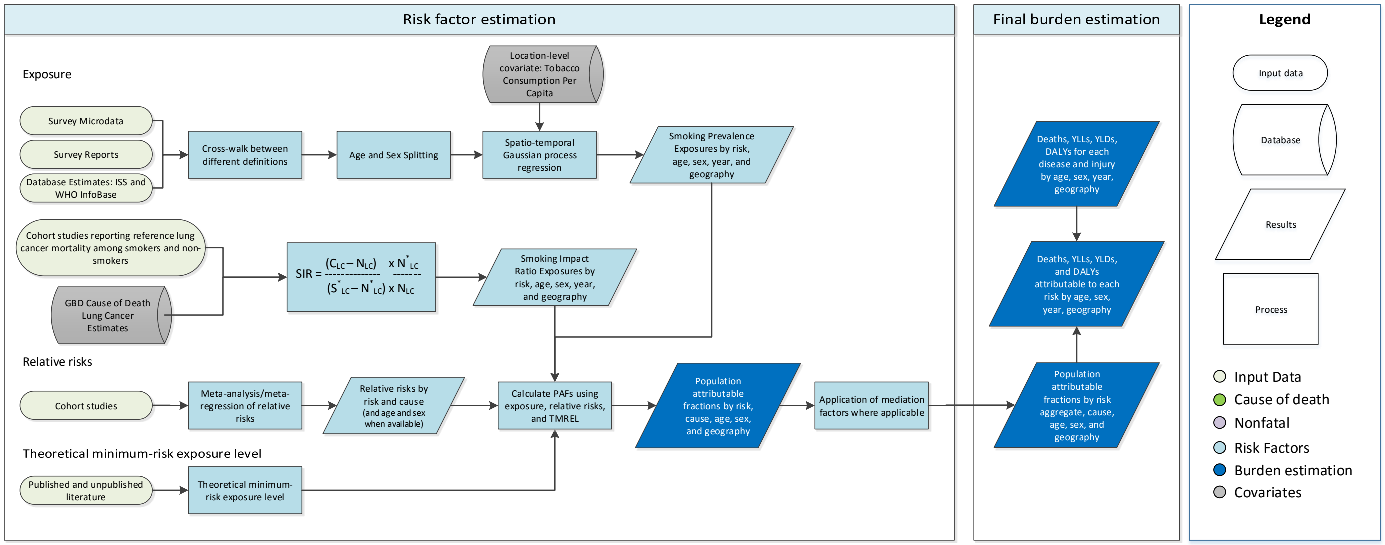

Input data & methodological summary

Exposure

Case definition

We used the Smoking Impact Ratio (SIR) for modelling burden attributable to smoking for cancers, chronic obstructive pulmonary disease (COPD), interstitial lung disease, other chronic respiratory diseases, and pneumoconiosis. SIR is the population lung cancer mortality in excess of lung cancer mortality among never-smokers, relative to excess lung-cancer mortality observed in a known reference group of smokers. Currently, SIR is adjusted to account for differences in baseline never-smoker lung cancer mortality across geography, age, and sex, but not for differences across time.

We used 5-year lagged smoking prevalence for modelling burden attributable to smoking for cardiovascular diseases, TB, diabetes, lower respiratory infections, asthma, cataracts, macular degeneration, fractures, rheumatoid arthritis, and peptic ulcer disease. Smoking is a dichotomous exposure defined as current daily use of smoked tobacco.

A full list of outcomes included in GBD 2015 and their exposure definition is available in the table below.

Outcome	Exposure
Atrial fibrillation and flutter	5-year lagged smoking prevalence
Aortic aneurysm	5-year lagged smoking prevalence
Hypertensive heart disease	5-year lagged smoking prevalence
Ischaemic heart disease	5-year lagged smoking prevalence
Other cardiovascular and circulatory diseases	5-year lagged smoking prevalence

Peripheral vascular disease	5-year lagged smoking prevalence
Haemorrhagic stroke	5-year lagged smoking prevalence
Ischaemic stroke	5-year lagged smoking prevalence
Diabetes	5-year lagged smoking prevalence
Lower respiratory infections	5-year lagged smoking prevalence
Asthma	5-year lagged smoking prevalence
Tuberculosis	5-year lagged smoking prevalence
Peptic ulcer disease*	5-year lagged smoking prevalence
Rheumatoid arthritis*	5-year lagged smoking prevalence
Cataract*	5-year lagged smoking prevalence
Macular degeneration*	5-year lagged smoking prevalence
Hip fracture*	5-year lagged smoking prevalence
Non-hip fracture*	5-year lagged smoking prevalence
Bladder cancer	Smoking Impact Ratio (SIR)
Colon and rectum cancer	Smoking Impact Ratio (SIR)
Oesophageal cancer	Smoking Impact Ratio (SIR)
Kidney cancer	Smoking Impact Ratio (SIR)
Leukaemia	Smoking Impact Ratio (SIR)
Liver cancer	Smoking Impact Ratio (SIR)
Tracheal, bronchus, and lung cancer	Smoking Impact Ratio (SIR)
Lip and oral cavity cancer	Smoking Impact Ratio (SIR)
Nasopharynx cancer	Smoking Impact Ratio (SIR)
Pancreatic cancer	Smoking Impact Ratio (SIR)
Stomach cancer	Smoking Impact Ratio (SIR)
Larynx cancer*	Smoking Impact Ratio (SIR)
Chronic obstructive pulmonary disease	Smoking Impact Ratio (SIR)
Interstitial lung disease and pulmonary sarcoidosis	Smoking Impact Ratio (SIR)
Other chronic respiratory diseases	Smoking Impact Ratio (SIR)
Pneumoconiosis	Smoking Impact Ratio (SIR)

* New outcome in GBD 2015

Input data

Consistent with GBD 2013, we used nationally representative survey data to estimate smoking prevalence. Survey and report data identified in the Global Health Data Exchange (GHDx), the WHO InfoBase, and the International Smoking Statistics (ISS) Database.

Inclusion criteria

- *Nationally representative*
- *Report current use of any of the following frequency-type combinations:*
    - *Daily use of smoked tobacco*
    - *Any use (both daily and occasional) of smoked tobacco*
    - *Daily use of cigarettes*
    - *Any use (both daily and occasional) of cigarettes*
    - *Daily use of any tobacco (both smoked and smokeless)*
    - *Any use (both daily and occasional) of any tobacco (both smoked and smokeless)*
    - *Daily use of any tobacco excluding cigarettes*

- *Report data within the time period of* January 1, 1980 – December 31, 2015 for any geography estimated in the GBD framework
- Smoking prevalence reported among individuals ages 10+

Global Health Data Exchange (GHDx)
Sources were identified through a systematic search of the GHDx.

- Search Terms (Keywords): Tobacco Use
- Time Period: January 1, 1980 – December 31, 2015
- Data Type: Survey OR Report
- Search Date: February 16, 2016

Out of 3,912 sources identified in the GHDx, 2,818 sources were included.

WHO InfoBase and International Smoking Statistics (ISS) Database
An effort was made to replace database-derived estimates used in GBD 2013 with original extractions from primary data sources. In GBD 2013, [851] sources were derived from the WHO InfoBase or the ISS Database. In GBD 2015, we replaced [257] sources with extractions from primary data sources and continued to use [594] sources from the WHO InfoBase (n=[281]) and the ISS Databse (n=[313]).

Outliers
Throughout the modelling process, data were assessed for bias and outliers were flagged. A data point was flagged as a candidate outlier if it was not consistent with the majority of other data points in a country with respect to level, age-pattern, sex-pattern, or temporal trend. In data-scarce countries, data points were also compared to data from other countries in a region. Candidate outliers were scrutinised for potential sources of bias and were ultimately excluded if the point or source was deemed to not be representative.

Modelling strategy

Data extraction

When possible, we extracted individual smoking status for all available frequency-type categories (listed above) from person-level microdata and collapsed these data to produce prevalence estimates in the standard GBD 5-year age-sex groups. If microdata were unavailable we extracted the most granular age-sex groups available from survey reports. Any available measures of uncertainty were extracted, including standard error, confidence or uncertainty intervals, and sample size.

Data preparation: crosswalking
Regressions to crosswalk other frequency-type categories to the gold-standard definition of daily use of smoked tobacco were estimated in the form:

$$p_{daily-smoked,k} = \beta_1 p_{i,k} + \epsilon_k$$

where $p_{daily\text{-}smoked,k}$ is the prevalence of daily smoking reported in survey k, and $p_{i,k}$ is the prevalence of an alternative frequency-type combination i also reported in survey k. Consistent with previous GBD smoking crosswalks, the intercept was omitted from the regression. The estimated regression coefficient β_1 was used to crosswalk alternative frequency-type categories to the gold-standard daily smoking definition in

sources only providing the alternative category. Predication error at the data-point level was used to propagate uncertainty and was calculated using the following equation:

$$\mathrm{PE_k} = \sigma_\epsilon^2 + \mathrm{X_k^2 var}(\hat{\beta})$$

Compared to the separate frequency and type crosswalks used in GBD 2013, the combined frequency-type crosswalk used in GBD 2015 represents an improvement because patterns in frequency that may vary by type and patterns in type that may vary by frequency are captured.

Data preparation: age and sex splitting

Report data provided in age groups wider than the standard GBD 5-year age groups or as both sexes combined were split using the approach used in Ng et al. Briefly, age-sex patterns were identified using sources with data on multiple age-sex groups and these patterns were applied to split aggregated report data. Uncertainty in the age-sex split was propagated by multiplying the standard error of the data (including the predication error of the crosswalk) by the square root of the number of splits performed.

Modelling: Linear Model

After data preparation, the dataset consisted of prevalence estimates of daily smoked tobacco use in standard GBD country-year-age-sex groups. The mean function used in ST-GPR was estimated using the following hierarchical mixed-effects linear regression, run separately by sex:

$$\mathrm{logit}(p_{c,a,t}) = \beta_0 + \beta_1 \mathrm{CPC}_{c,t} + \sum_{k=2}^{16} \beta_k I_{A[a]} + \alpha_s + \alpha_r + \alpha_c + \epsilon_{c,a,t}$$

where $CPC_{c,t}$ is the annual tobacco consumption per capita covariate, $I_{A[a]}$ is a dummy variable indicating specific age group A that the prevalence point $p_{c,a,t}$ is capturing, and α_s, α_r, and α_c are super-region-, region-, and country-specific random effects.

Modelling: spatiotemporal Gaussian process regression (ST-GPR)

The estimated mean function was then propagated through the ST-GPR framework to obtain 1,000 draws of smoking prevalence estimates for each location, year, age, and sex. Parameter selection for the ST-GPR hyper-parameters were selected through out-of-sample cross-validation using the strategy described elsewhere in this appendix.

Smoking Impact Ratio estimation

We have made no substantive changes in the SIR estimation strategy from GBD 2013. The only change in input data for estimating never-smoker lung-cancer mortality was to update data from the China Kadoorie Biobank prospective cohort to include follow-up through 2014. Country-year-age-sex-specific lung cancer mortality rates are derived from GBD 2015 Cause of Death estimation and detailed in that capstone's appendix. The formula for calculating SIR is:

$$SIR = \frac{C_{LC} - N_{LC}}{S_{LC}^* - N_{LC}^*} \times \frac{N_{LC}^*}{N_{LC}}$$

C_{LC}: age-sex-specific lung cancer mortality rate in the population of interest
N_{LC}: age-sex-specific lung cancer mortality rate of never-smokers in the population of interest
S^*_{LC}: age-sex-specific lung cancer mortality rate for life-long smokers in a reference population
N^*_{LC}: age-sex-specific lung cancer mortality rate for never smokers in the reference population

Theoretical minimum-risk exposure level

The theoretical minimum-risk exposure level is that no one in the population smokes tobacco; that is, the smoking impact ratio is zero and smoking prevalence is zero.

Relative risk

We have made no substantive updates to relative risks for outcomes included in GBD 2013. The following outcomes using 5-year lagged smoking prevalence as the exposure were added in GBD 2015: peptic ulcer disease, rheumatoid arthritis, cataracts, macular degeneration, hip fracture, and non-hip fracture. Larynx cancer was the only new outcome added using SIR as the exposure. Relative risks for rheumatoid arthritis, cataracts, and macular degeneration were derived from recent published meta-analyses. We performed out own meta-analyses of prospective cohort studies to derive relative risks for peptic ulcer disease, hip fracture, and non-hip fracture. We used Kontis and colleagues' re-analysis of CPS-II smokers for the relative risk of larynx cancer.

Ambient particulate matter risk factor estimation process

Ambient PM2.5

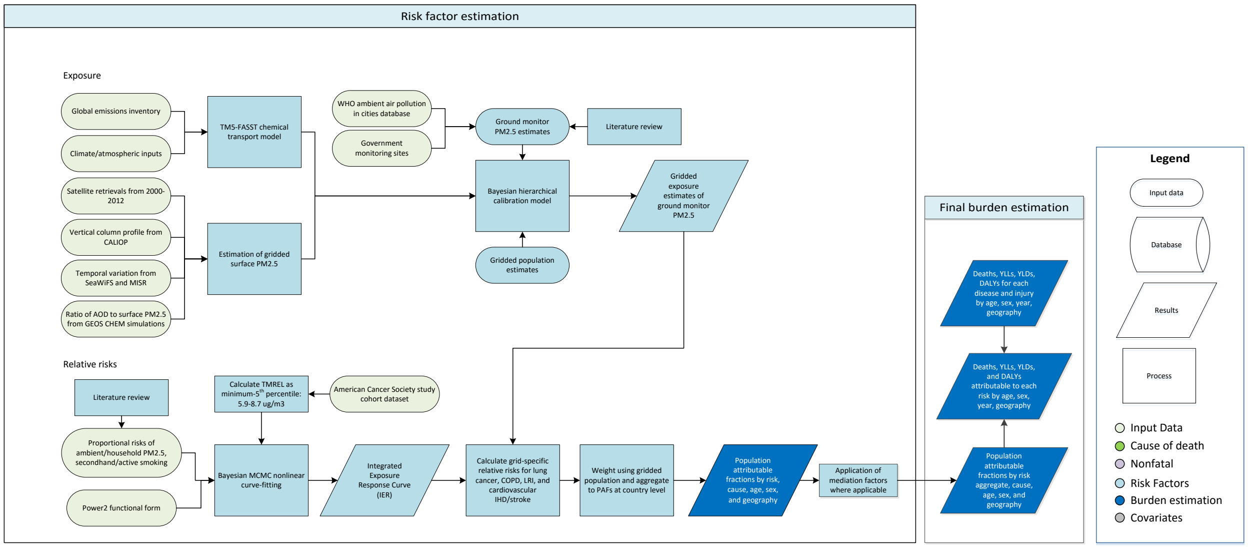

Input data & modelling strategy

Exposure

Definition

Exposure to ambient air pollution is defined as the population-weighted annual average mass concentration of particles with an aerodynamic diameter less than 2.5 micrometers ($PM_{2.5}$) in a cubic meter of air. This measurement is reported in µg/m^3.

Input data

The data to estimate exposure to ambient air pollution is drawn from estimates of annual concentration of $PM_{2.5}$ – generated using satellite observations of aerosols in the atmosphere. To correct for bias in the satellite modelling approach, a spatially varying flexible framework is used to combine modelled concentrations with observations from ground-level monitoring of particles in more than 75 countries. All input data for GBD 2015 were updated as follows:

Updated $PM_{2.5}$ ground measurement database

For the GBD 2015 update we updated the database of annual average PM measurements to include more recent data and to incorporate additional locations where measurement data have become available. To facilitate this we collaborated with WHO and contributed to their recently released WHO Air Pollution in Cities database. We then used disaggregated (monitor-specific values and not the city averages that are reported by WHO) measurements from this database with additional site-specific information (eg, all monitors in a city, monitor geo coordinates, monitor site type) such as that included in the GBD 2013 database. In total, measurements of concentrations of PM_{10} and $PM_{2.5}$ were retrieved from 6,003 ground monitors with the majority contributing measurements from 2014 (as there is a lag in reporting measurements, few data from 2015 were available). Where data were not available for 2014 (2,760 monitors), data were used from 2015 (18 monitors), 2013 (2,155), 2012 (564), 2011 (60), 2010 (375),

2009 (49), 2008 (21) and 2006 (1). For locations with only PM_{10} measurements, $PM_{2.5}$ measurements were estimated from PM_{10}. This was done by a locally derived conversion factor ($PM_{2.5}/PM_{10}$ ratio) estimated as population-weighted averages of location-specific conversion factors for the country. Location-specific conversion factors were estimated as the mean ratio of $PM_{2.5}$ to PM_{10} of stations for the same year. If national conversion factors were not available, regional ones were used, which were obtained by averaging country-specific conversion factors.

Updated satellite-based estimates

The updated satellite-based estimates are described in detail in van Donkelaar and colleagues, 2016[1]. These estimates (~11 x 11 km resolution at the equator) combine aerosol optical depth retrievals from multiple satellites with the GEOS Chem chemical transport model and land use information.

Updated population data

A comprehensive set of population data on a high-resolution grid was obtained from the Gridded Population of the World (GPW v4) database. These data are provided on a $0.0417^{o}\times0.0417^{o}$ resolution. To aggregate these estimates of population to each $0.1^{o}\times0.1^{o}$ grid cell, the central 3 × 3 population cells were summed. As this accounted for a resolution higher than necessary, the same was done four other times, offset by one cell in a North, South, East, and West direction. The average of five quantities was used as the aggregated population estimate for each cell. Estimates of population for 2000, 2005, 2010, 2015, and 2020 were extracted from GPW version 4, and estimates for 1990 and 1995 were extracted from GPW version 3 as described previously for GBD 2013[3].

Modelling strategy

The methodology used to estimate the burden of ambient particulate matter pollution has seen significant changes since GBD 2013.

The GBD 2010 and GBD 2013 estimates both used a single global function to calibrate the mean of the chemical transport model and satellite-based estimates to available ground measurements. In both instances the approach taken was recognised at the time to be a compromise between what could be easily implemented under tight timeframes and one that most efficiently utilised all of the data sources. In particular, the GBD 2013 exposure estimates were known to underestimate ground measurements in specific locations (see discussion in Brauer and colleagues, 2015[2]) such that it would be desirable to allow measurements to make a larger contribution to the final estimates where they were available. Therefore, for GBD 2015 we implemented a Bayesian hierarchical modelling approach using Integrated Nested Laplace Approximations (INLA) in which the satellite-based estimates, ground measurements, and land use information are combined in a spatially varying flexible framework. Formal external evaluation using ground measurements was conducted and shown to lead to improved predictions of ground measurements in all super-regions compared to GBD 2013 estimates and an alternative geographically weighted regression approach. Further, based on the external evaluation analyses, addition of the TM5 chemical transport model estimates of $PM_{2.5}$ annual average did not improve the estimates and these were therefore not included.

Bayesian hierarchical models (BHM) provide an extremely useful and flexible framework in which to model complex relationships and dependencies in data. Uncertainty can also be propagated through the model, allowing uncertainty arising from different components, both data sources and models, to be propagated through the models into estimates of uncertainty associated with the final estimates. In the hierarchical modelling approach coefficients associated with satellite-based estimates were estimated for each country. Where data were insufficient within a country, information can be “borrowed” from a higher aggregation (region) and if enough information is still not available, from an even higher level (super-region). Individual country-level estimates were therefore based on a combination of information from the country, its region, and its super-region.

All modelling was performed on the log-scale with the unit of measurement being a grid cell. The model was constructed with the inclusion of all variables assessed statistically, based on model fit (DIC, a measure of the relative quality of a model and closely related to that of AIC but for Bayesian models) and predictive ability. The hierarchical structure was applied to the intercept and slope terms with all modelling on the log scale. The model was of the form

$$\log(PM2.5_i) = \beta_0 + \beta_1 \log SAT_i + other\ variables + \varepsilon_i$$

where *i* denotes the grid cell. The following sets of variables were considering in developing the models:

Continuous explanatory variables:

- (SAT) Estimate of $PM_{2.5}$ (in μgm^{-3}) for 2014 from satellite remote sensing on the log-scale
- (CTM) Estimate of $PM_{2.5}$ (in μgm^{-3}) for 2014 from chemical transport models on the log-scale
- Estimate of population for 2014 on the log-scale.
- (SNAOC) Estimate of the sum of sulfate, nitrate, ammonium, and organic carbon as estimated from GEOS Chem
- (DST) Estimate of compositional concentrations for mineral dust from GEOS Chem
- (EDxDU) The log of the elevation difference between the elevation at the ground measurement location and the mean elevation within the GEOS Chem simulation grid cell multiplied by the inverse distance to the nearest urban land surface

Discrete explanatory variables:

- Binary variable indicating whether exact location of ground measurement is known
- Binary variable indicating whether exact type of ground monitor is known
- Binary variable indicating whether ground measurement is $PM_{2.5}$ or converted from PM_{10}

Random effects:

- Grid cell random effects on the intercept to allow for multiple ground monitors in a grid cell
- Country-region-super-region hierarchical random effects for the intercept
- Country-region-super-region hierarchical random effects for the satellite remote sensing term

- Country-region-super-region hierarchical random effects for the coefficient associated with the difference between estimates from CTM and SAT
- Country-region-super-region hierarchical random effects for the coefficient log(POP)
- Country-level random effects for intercept, satellite, and difference between CTM and SAT are independent and identically distributed
- Country-level random effects for population uses a neighbourhood structure allowing specific borrowing of information from neighbouring countries
- All region random effects are assumed to be independent and identically distributed
- All super-region random effects are assumed to be independent and identically distributed

Interactions*:*

- Interactions between the binary variables and the effects of log(SAT) and log(CTM/SAT)

Due to both the complexity of the models and the size of the data, notably the number of spatial predictions that are required in this setting, recently developed techniques that perform "approximate" Bayesian inference based on integrated nested Laplace approximations (INLA) have been developed as a computationally attractive alternative to Markov Chain Monte Carlo methods. Computation was performed using the R interface to the INLA computational engine (R-INLA) with the size of the task of fitting the models and performing predictions for each of the ca. 1.4 million grid cells requiring the use of a high-performance computing cluster (HPC) with high memory nodes. As in GBD 2010 and GBD 2013 the spatial model was built combining the different data sources for a single year (2014, corresponds to the most recent measurement data). The spatially varying functions from this model were then applied to the satellite-based estimates from all other years – in other words, assuming that the spatial relationship between the different data sources does not change over time. This is undoubtedly a simplification but to do otherwise would require assembling multi-year measurement databases, which is not feasible given current data availability and computational constraints. As the spatial model was built using the most recently available (2014) measurement and satellite-based estimates, 2015 estimates were based on extrapolation. Instead of extrapolating using an exponential model based on a one-year trend as in GBD 2013, splines based on a five-year trend (2010–2014) were fit and applied to the 2014 grid-cell values to estimate levels for 2015. This reduced the likelihood of 2015 estimates being overly influenced by meteorological events in a specific year and to better represent the duration of exposure relevant to the epidemiologic studies included in the integrated exposure-response functions.

Model evaluation

Model evaluation and comparison was performed by fitting models on a training set and predicting exposures at locations for which measurements were known (the validation set). The selection of the training (20%) and validation (80%) set consisted of taking a random sample of the total number of sites measuring $PM_{2.5}$ (or having a value converted from PM_{10} measurements). Sampling was performed using sampling probabilities based on the cross-tabulation of $PM_{2.5}$ categories (0–24.9, 25–49.9, 50–74.9, 75–99.9, 100+ μg/m^3) and super-regions. The resulting holdout evaluation dataset was a sample of 20% of the sites that have the same distribution over $PM_{2.5}$ categories and super-regions as the entire set of sites.

This process was used to generate multiple training and validation set combinations, allowing for example cross-validation to be performed. In the evaluation, 25 sets of training/validation data were used. The following models were considered in the evaluation phase:

(A) The GBD 2013 model, using a simple linear regression with a fused estimate of SAT and CTM together with interactions with three binary variables representing whether the measurement was converted from PM_{10} and whether the exact site type and location is known
(B) A hierarchical model with SAT, the TM5 CTM estimates, population, and the three binary variables described above
(C) A hierarchical model with SAT, population, SNAOC, DST, EDxDU, population, and the three binary variables
  - Estimate of population for 2014 on the log-scale
  - Estimate of the sum of sulfate, nitrate, ammonium, and organic carbon as estimated from GEOS Chem
  - Estimate of compositional concentrations for mineral dust from GEOS Chem
  - The log of the elevation difference between the elevation at the ground measurement location and the mean elevation within the GEOS Chem simulation grid cell multiplied by the inverse distance to the nearest urban land surface

For each training/evaluation set combination, model fit and prediction accuracy were evaluated for each of the 25 training/evaluation set combinations with the following metrics:

Model fit

- R^2
- DIC

Predictive accuracy

- R^2 arising from a linear regression of predicted versus actual measurements at each location
- RMSE – root mean squared error
- WRMSE – weighted (by population) root mean squared error
- MSE – mean square error
- MAE – mean absolute error

This evaluation indicated the final model improved predictions of ground measurements in all super-regions compared to GBD 2013 estimates (median global R^2 [population-weighted RMSE] 0.82 (12.1 $\mu g/m^3$), 0.60 [13.5 $\mu g/m^3$] for GBD 2015 and GBD 2013, respectively).

Figure 1 shows the RMSE (median from the 25 runs) for each of the three models, by super-region. The accuracy of the prediction varies between super-regions, with lower errors being observed in areas where there are more monitoring sites. In each of the super-regions, the largest errors are seen for model A which are considerably higher than those for models B and C, with model C showing a small improvement over B (except in super-region 5, North Africa/Middle East).

Figure 2 shows scatter plots of the observed and predicted measurements using the three models for each super-region. The predicted measurements are the median values over those obtained from the 25 training sets. Predictions from the two Bayesian hierarchical models (B&C) match the observed values more closely than the linear model (A), with much less spread around a straight line (with slope one and zero intercept, shown in red). In Central Europe and sub-Saharan Africa it is noticeable that, in addition to

reduced spread, models B and C are much better at predicting higher values. The same patterns of results in predictive ability were seen when looking at regions and individual countries. In all cases, model C performed better than model B, with both being considerably better than model A.

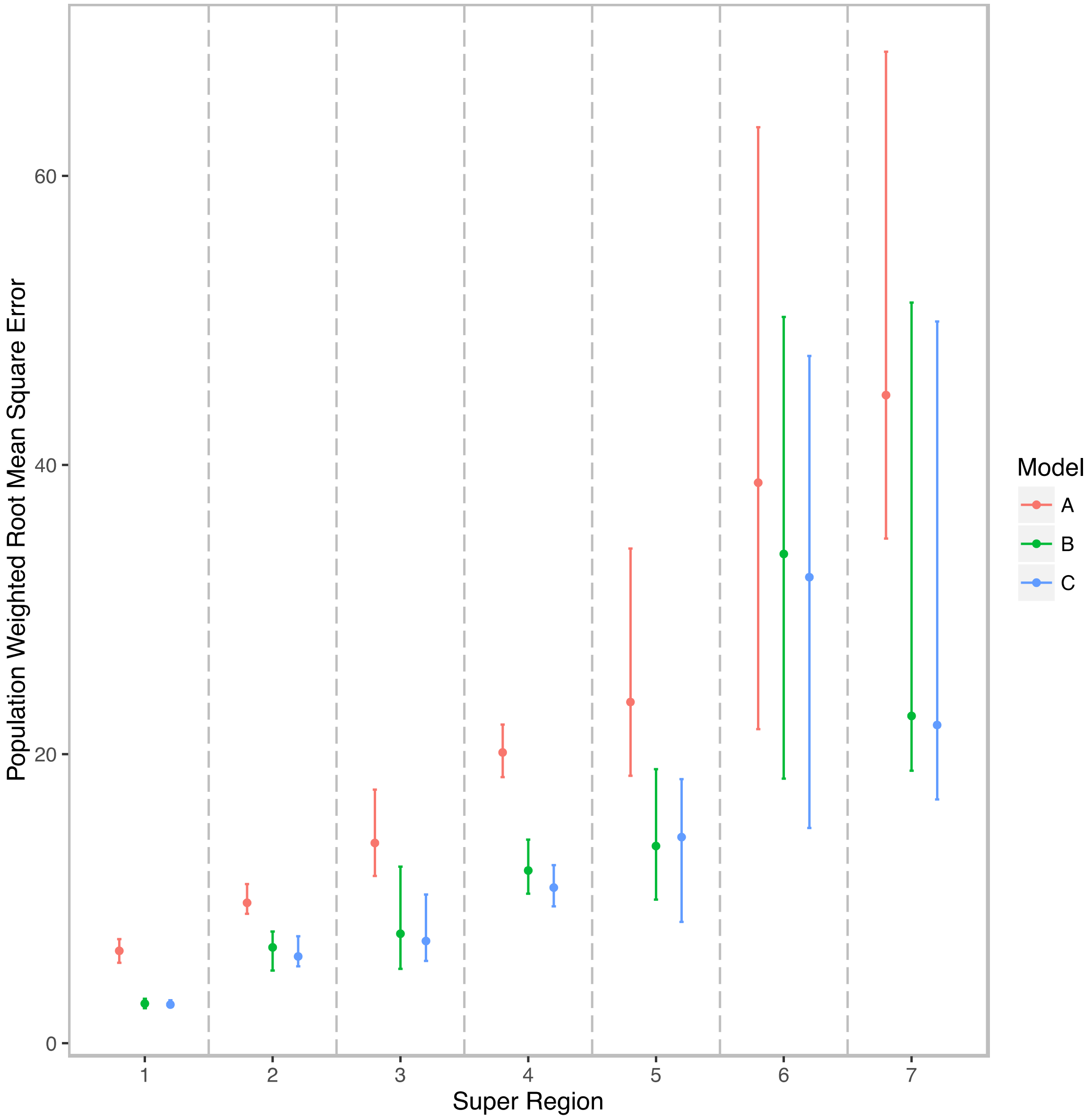

Figure 1: Comparison of RMSE from three models by super-region. Dots denote the median of the distribution from 25 training/evaluation sets and the vertical lines the range of values. Super-regions are 1: High-income, 2: Central Europe, Eastern Europe, Central Asia, 3: Latin America and Caribbean, 4: Southeast Asia, East Asia, and Oceania, 5: North Africa/Middle East, 6: Sub-Saharan Africa, 7: South Asia.

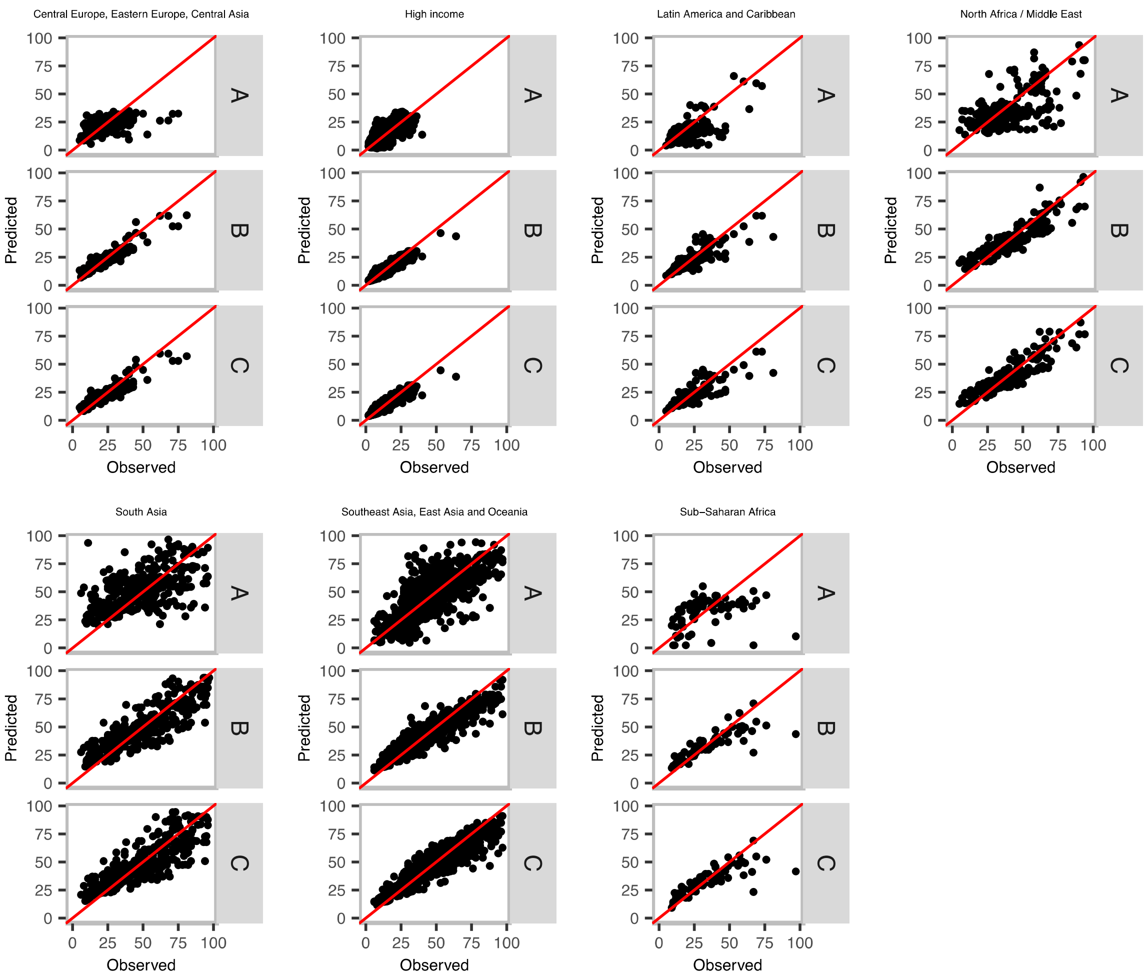

Figure 2: Comparison of observed and predicted measurements using three different models, by super-region. The red line has slope one and intercept zero.

Overall, the best model in terms of model fit and predictive ability was one with the following components:

- Estimates of $PM_{2.5}$ (in μgm^{-3}) from satellite remote sensing (SAT), population, and information on the GEOS Chem simulated chemical composition, elevation, and distance to urban land use (SNAOC, DST, and EDxDU).
- Binary variables indicating whether exact location and type of ground measurement is known, and whether the measurement was $PM_{2.5}$ or converted from PM_{10}.
- Interactions between the binary variables and the effects of estimates from satellite remote sensing.
- Grid cell random effects on the intercept to allow for multiple ground monitors in a grid cell.
- Country-region-super-region hierarchical random effects for intercepts, satellite remote sensing, and population terms.
- Country-level random effects for population using a neighbourhood structure allowing specific borrowing of information from neighbouring countries.

Theoretical minimum-risk exposure level

The TMREL for ambient PM is estimated using a uniform distribution between the minimum and fifth percentile of exposure observed in the studies used to generate the GBD estimates. This estimate was updated for GBD 2015 as new studies were added to the analysis and studies used previously were updated through continued follow-up. The newer estimates included several large studies that included exposure at lower levels of $PM_{2.5}$. As a result, the TMREL for GBD 2015 was ~U(2.4, 5.9), lower than GBD 2013's distribution ~U(5.9, 8.7), which had the effect, all things being equal, of increasing the estimated attributable burden relative to the GBD 2013 estimates.

Relative risk

Relative risks are generated using integrated exposure-response functions (IER) that are fit to available epidemiologic data using a Bayesian MCMC approach and a modified power function. The IER are estimated based on published relative risks for long-term exposure to ambient $PM_{2.5}$, household air pollution, secondhand smoking, and active (cigarette) smoking. The concentration of particulate matter for each type of exposure is estimated based on literature values and used to map the relative risks to a curve generated for the entire range of exposure from these sources. The input data for this curve-fitting process has been updated since GBD 2013, adding new studies that estimate exposure at finer spatial scales, including studies of within-city exposure that focus on traffic-related air pollution. In addition, changes were made to the curve-fitting process. In order to account for differences in study design, temporal patterns of exposure and other differences among the studies of the different sources of $PM_{2.5}$, a source-specific heterogeneity parameter was added to the IER. This resulted in much wider, and, in our view, more realistic, uncertainty intervals for the burden estimates, by propagating through the entire process the current uncertainty regarding the mechanisms and magnitude of health impacts of exposure to $PM_{2.5}$ from diverse sources.

IER functional form

Data Likelihood

$$\log(RR_i) \sim \mathcal{N}(\mu_i, \sqrt{\sigma_i^2 + \delta_{source_i}})$$

Model

$$\mu_i = \log\left(\frac{1+\alpha \times \left(1 - e^{-\beta \times (exposure_i - TMREL)^\gamma}\right)}{1+\alpha \times \left(1 - e^{-\beta \times (counterfactual_i - TMREL)^\gamma}\right)}\right)$$

Data

RR_i : measured relative risk for data point i
σ_i : variance of data point i based on study information
$source_i$: exposure source type (outdoor/household air pollution, secondhand/active smoking)
$TMREL$: theoretical minimum risk exposure level
$exposure_i$: measured exposure for data point i
$counterfactual_i$: counterfactual exposure for data point i

Priors

$$\alpha \sim \Gamma(1.0, 0.01)$$
$$\beta \sim \Gamma(1.0, 0.01)$$
$$\gamma \sim \Gamma(1.0, 0.01)$$
$$\delta \sim \Gamma(1.0, 0.01)$$

We also modified the way in which age-specific IER for IHD and stroke were estimated. In accordance with previously published work on other cardiovascular risk factors, the impact of air pollution on cardiovascular health is known to vary with age. To account for this phenomenon, age-specific RRs were based on a log-linear model of RR as a function of age, where the intercept (RR=1) is forced to age 110. In GBD 2010 and GBD 2013 the age for a relative risk estimate from a given study was estimated as the median age at death or disease incidence in that study. However, this may not accurately represent the age distribution of the entire study population, so we re-estimated this variable as the mean age at enrollment plus half of the average follow-up time to better represent the average age of the study population during the period of follow-up. When compared to GBD 2013, this change produced RRs that were generally lower for younger age groups, given that median age at event tends to produce a higher anchor age than average age during follow-up.

The relative risks are generated on the grid level based on estimated exposure, and then applied to generate PAFs. These PAFs are aggregated using the grid-level population to create population-weighted national estimates of attributable burden, using the following formula:

$PM_{2.5}$ aggregation formula

$$PAF_{A,\ C,\ L} = \frac{\sum ((RR_{A,\ C} - 1)\ *\ Pop_i)}{\sum(RR_{A,\ C}\ *\ Pop_i)}$$

A = age group, C = cause, L = location, i = grid, $RR_{A,\ C}$ = grid-level RR based on $PM_{2.5}$ and given age/cause IER curve

References

1. van Donkelaar, A.; Martin, R. V; Brauer, M.; Hsu, N. C.; Kahn, R. A.; Levy, R. C.; Lyapustin, A.; Sayer, A. M.; Winker, D. M. Global Estimates of Fine Particulate Matter using a Combined Geophysical-Statistical Method with Information from Satellites, Models, and Monitors. *Environ. Sci. Technol.* 2016, *50* (7), 3762–3772.

2. Brauer, M.; Freedman, G.; Frostad, J.; van Donkelaar, A.; Martin, R. V; Dentener, F.; Van Dingenen, R.; Estep, K.; Amini, H.; Apte, J. S.; et al. Ambient Air Pollution Exposure Estimation for the Global Burden of Disease 2013. *Environ. Sci. Technol.* 2015, *50* (1), 79–88.

3. Brauer, M.; Amann, M.; Burnett, R. T.; Cohen, A.; Dentener, F.; Ezzati, M.; Henderson, S. B.; Krzyzanowski, M.; Martin, R. V; Van Dingenen, R.; et al. Exposure assessment for estimation of the global burden of disease attributable to outdoor air pollution. *Environ. Sci. Technol.* 2012, *46* (2), 652–660.

Household air pollution risk factor estimation process

Household Air Pollution from Solid Fuels

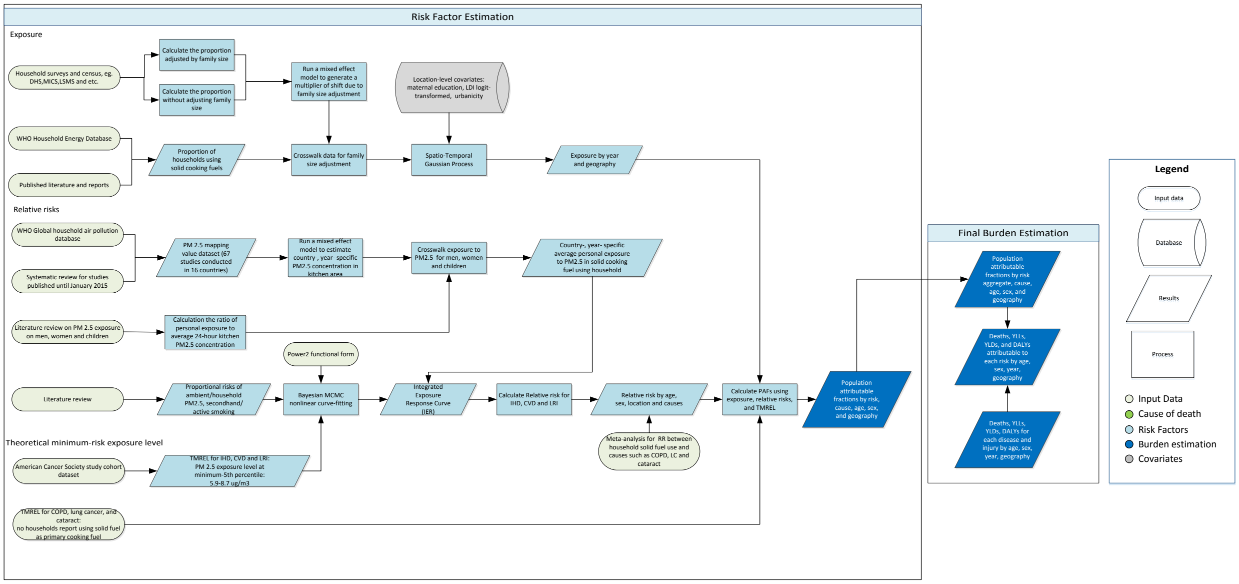

Input data & methodological summary

Exposure

Case definition

Exposure to household air pollution from solid fuels (HAP) is defined as the proportion of households using solid cooking fuels. The definition of solid fuel in our analysis includes coal, wood, charcoal, dung, and agricultural residues.

Input data

Data were extracted from the standard multi-country survey series such as Demographic and Health Surveys (DHS), Living Standards Measurement Surveys (LSMS), Multiple Indicator Cluster Surveys (MICS), and World Health Surveys (WHS), as well as country-specific survey series such as Kenya Welfare Monitoring Survey and South Africa General Household Survey. To fill the gaps of data in surveys and censuses, we also downloaded and updated HAP estimates from WHO Energy Database and extracted from literature through systematic review done at IHME. Each nationally or subnationally representative data point provided an estimate for the percentage of households using solid cooking fuels. Estimates for the usage of solid fuels for non-cooking purpose were excluded, ie, primary fuels for lighting. The database, with estimates from 1980 to 2015, contained 685 studies from 150 countries. Updates to systematic reviews are performed on an ongoing schedule across all GBD causes and risk factors; an update for household air pollution will be performed in the next one to two iterations.

Modelling strategy

Household air pollution was modelled at household level using a three-step modelling strategy ST-GPR that uses linear regression, spatiotemporal regression, and Gaussian Process Regression (GPR). The first step is a mixed-effect linear regression of logit-transformed proportion of households using solid cooking fuels. The linear model contains maternal education and proportion of population living in urban areas as covariates and has nested random effect by country, GBD region, and GBD super-region, respectively. The full ST-GPR process is specified elsewhere in this appendix.

Compared with GBD 2013, we have made changes in terms of the covariates utilised in the linear model. A variety of combinations of socioeconomic and environmental covariates in different transformation format were tested by running mixed-effect models with exposure data. The final list of covariates included in the exposure model are maternal education and the proportion of population living in urban area.

Theoretical minimum-risk exposure level

For outcomes where we extracted RR based on direct epidemiological evidence, ie, COPD, lung cancer, and cataract, TMREL was defined such that no households would report using solid fuel as their primary cooking fuel. For outcomes that utilise evidence based on the Integrated Exposure Response (IER), the TMREL is defined as uniform distribution between 33.3 and 41.9 $\mu g/m^3$. TMREL for household air pollution did not change from GBD 2013.

Relative risks

The disease-outcomes paired with household air pollution have not changed since GBD 2013. The list of outcomes paired with household air pollution has not changed since GBD 2013, which included lower respiratory infections (LRI), stroke, ischemic heart disease (IHD), chronic obstructive pulmonary disease (COPD), lung cancer, and cataract. The relative risks of all outcomes except cataract were generated by using the integrated exposure-response functions (IER). The relative risks for cataract were extracted from a meta-analysis paper (1). The IER curves are updated to reflect the newly updated data and utilization of a new method that is specified elsewhere.

$PM_{2.5}$ mapping value

The relative risk estimates describing the association of HAP with outcomes including ischemic heart disease (IHD), cardiovascular disease (CVD), and lower respiratory infections (LRI) were derived from the IER curves. This is done by first estimating the crosswalk values that map household use of solid fuel to $PM_{2.5}$ exposure because the IER curve measures exposure using $PM_{2.5}$. This step of the analysis relied on 67 studies conducted in 16 countries to generate the $PM_{2.5}$ mapping values, which remain the same sources as GBD 2013. The $PM_{2.5}$ exposure was then cross-walked to men, women, and children by generating the ratio of personal exposure to average 24-hour kitchen $PM_{2.5}$ concentration based on a study after the literature review in GBD 2013.

References

1. Smith KR, Bruce N, Balakrishnan K, Adair-Rohani H, Balmes J, Chafe Z, et al. Millions Dead: How Do We Know and What Does It Mean? Methods Used in the Comparative Risk Assessment of Household Air Pollution. *Annu Rev Public Health*. 2014;35(1):185–206.

Occupational risk factor estimation process

Occupational Risk Factors
(except abestos and injuries)

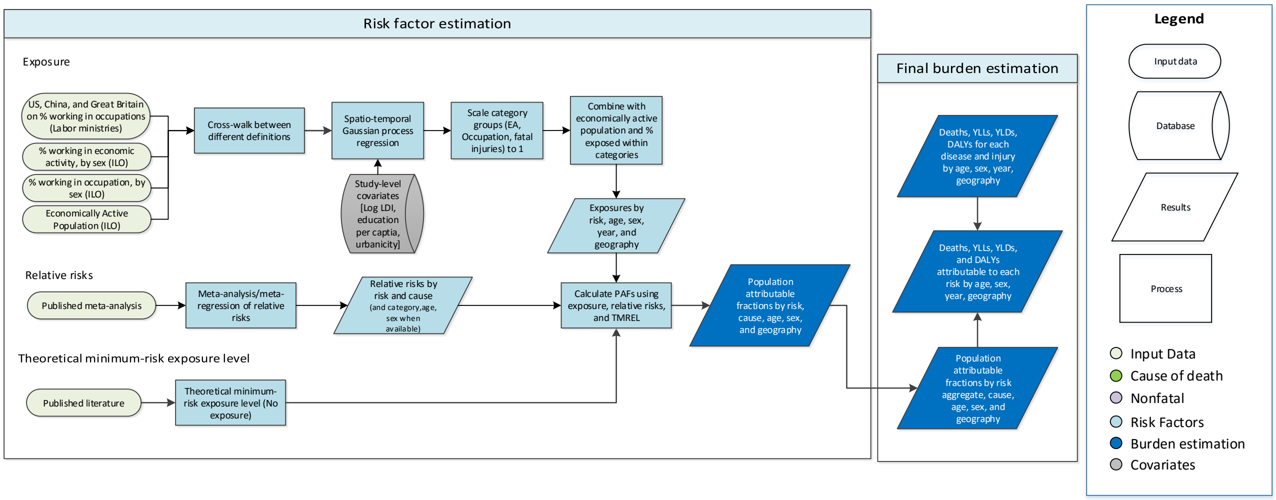

Occupational Risk Factors (Injuries)

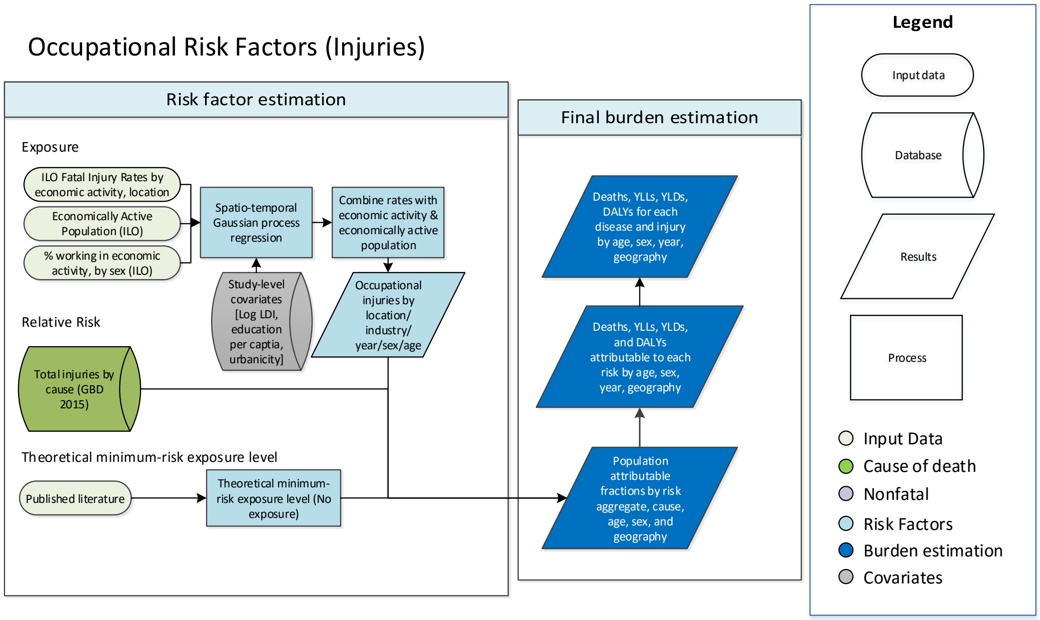

Occupational Risk Factors (abestos)

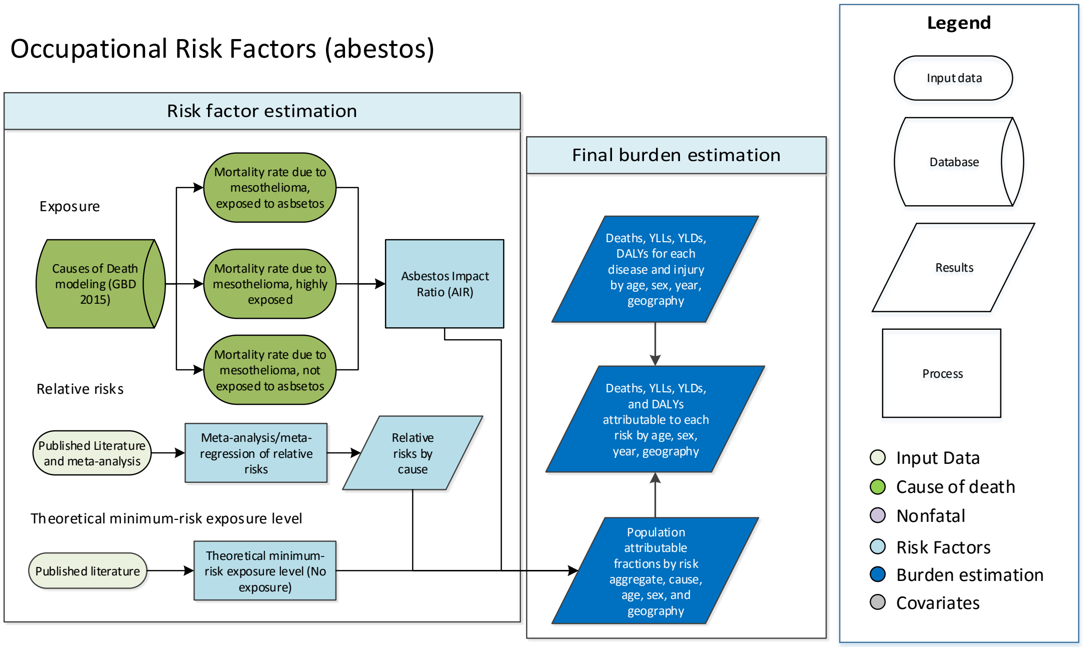

Input data and methodological summary

Exposure

Case definition

The following definitions were used for occupational risk factor exposures. All exposures were estimated only for ages 15+

Occupational asbestos	Cumulative exposure to occupational asbestos using mesothelioma death rate as an analogue
Occupational asthmagens	Proportion of working population exposed to asthmagens based on distribution of the population in seven occupational groups
Occupational carcinogens (arsenic, acid, benzene, beryllium, cadmium, chromium, diesel, formaldehyde, nickel, polycyclic aromatic hydrocarbons, secondhand smoke, silica, trichloroethylene)	Proportion of working population ever exposed to carcinogens in high- or low-exposure groups, based on distribution of the population in nine economic activity groups
Occupational injuries	Proportion of fatal injuries attributed to occupational work in nine economic activities, based on fatal injury rates in those economic activities
Occupational ergonomic factors	Proportion of working population exposed to lower back pain, based on distribution of the population in seven occupational groups

Occupational noise	Proportion of working population exposed to 85+ decibels of noise, based on distribution in nine economic activities
Occupational particulates	Proportion of working population exposed based on distribution in nine economic activities

Estimates of the proportion of population involved in economic activities and occupations were coded into the following categories:

**Economic activities**	**Occupations**
Agriculture, hunting, forestry, and fishing	Agriculture, animal husbandry, forestry workers, fishermen, and hunters
Mining and quarrying	Production, transport equipment operators and laborers, and related workers
Wholesale and retail trade, restaurants, and hotels	Professional, technical, and related workers
Manufacturing	Sales workers
Electricity, gas, and water	Administrative and managerial workers
Transport, storage, and communication	Clerical and related workers
Construction	Service workers
Financing, insurance, real estate, and business services	
Community, social, and personal services	

Input data

Primary inputs were obtained from the ILO [1-4], using raw data on economic activity proportions, occupation proportions, fatal injury rates, and economically active population estimates. For different ISIC classifications, estimates were recoded to one of the nine economic activities or occupations. Subnational estimates for UK and China were added to the datasets for economic activities and occupations [5-6].

For occupational asbestos, primary inputs were obtained through GBD 2015 cause of death estimates and published studies. [7,13-14]

Modelling strategy

A spatiotemporal Gaussian process regression was used to generate estimates for all year/locations for the primary inputs. Parameters were chosen by maximizing out-of-sample cross-validation and minimizing RMSE. For economic activity and occupation proportions, estimates from ST-GPR were then re-scaled to sum to 1 across categories by dividing each estimate by the sum of all the estimates.

The following sections describe the modelling approaches for each occupational risk's prevalence exposure.

Occupational carcinogens, occupational noise, occupational particulates

Prevalence of exposure to these risks was determined using the following equation:

$$Prevalence\ of\ Exposure_{c,y,s,a,r,l} = \sum_{EA} Proportion_{EA,c,y} * EAP_{c,y,s,a} * Exposure\ rate_{EA,r,l,d}$$

where:

EAP = economically active population	c = country	r = risk
EA = economic activity	d = duration	s = sex
a = age	l = level of exposure	y =year

Exposure rate was provided by expert group recommendations and literature [8-11] (see Table 1). Duration was only considered for occupational carcinogens, through application of occupational turnover factors [12].

Occupational ergonomic factors and asthmagens

Prevalence of exposure to these risks was determined using the following equation:

$$Prevalence\ of\ Exposure_{c,y,s,a,r} = \sum_{EA} Proportion_{OCC,c,y} * EAP_{c,y,s,a}$$

where:

EAP = economically active population	c = country	r = risk
OCC = occupation	a = age	s = sex
		y = year

Occupational injuries

Occupational injury counts were estimated using the following equation:

$$Occupational\ fatal\ injuries_{c,y,a,s} = \sum_{EA} Injury\ rate_{EA,c,y,s} * Population_{c,y,a,s} * EAP_{c,y,s,a} * Proportion_{EA,c,y}$$

where:

EAP = economically active population	c = country	y = year
EA = economic activity	a = age	s = sex

Occupational asbestos

Prevalence of exposure to asbestos was estimated using the asbestos impact ratio (AIR), which is equivalent to the excess deaths due to mesothelioma observed in a population divided by excess deaths due to mesothelioma in a population heavily exposed to asbestos. Formally, this is defined using the following equation:

$$AIR = \frac{Mort_{c,y,s} - N_{c,y,s}}{Mort^{*}_{c,ys,} - N_{c,y,s}}$$

where:

Mort = Mortality rate due to mesothelioma
Mort* = Mortality rate due to mesothelioma in population highly exposed to asbestos
N = Mortality rate due to mesothelioma in population not exposed to asbestos

c = country
y = year
s = sex

Mortality rate due to mesothelioma was estimated from GBD 2015 causes of death [7]. Mortality rate due to mesothelioma in population not exposed to asbestos was calculated using the model in Lin and colleagues [13], while the mortality rate due to high exposure to asbestos was estimated in Goodman and colleagues [14].

Theoretical minimum-risk exposure level

For all occupational risks, with the exception of occupational asbestos, the theoretical minimum-risk exposure level was assumed to be no exposure to that risk.

Relative risk

Relative risks were obtained for all occupational risks by conducting a systematic review of published meta-analysis.

Population attributable fraction

For all occupational risks, with the exception of injuries outlined below, PAFs were calculated using the prevalences estimated above, using the PAF formula.

Occupational injuries PAF

The PAF for occupational injuries was calculated using the following formula:

$$PAF_{c,y,a,s} = \frac{Occupational\ fatal\ injuries_{c,y,a,s} - TMREL}{Fatal\ injuries_{c,y,a,s}}$$

where:

c = country
y = year

a = age
s = sex

Fatal injuries total was obtained from GBD 2015 causes of death [7].

References

1. International Labour Organization (ILO). International Labour Organization Database (ILOSTAT) - Employment by Sex and Economic Activity. International Labour Organization (ILO).

2. International Labour Organization (ILO). International Labour Organization Database (ILOSTAT) - Employment by Sex and Occupation. International Labour Organization (ILO).

3. International Labour Organization (ILO). International Labour Organization Database (ILOSTAT) - Fatal Injuries by Sex and Economic Activity. International Labour Organization (ILO).

4. International Labour Organization (ILO). International Labour Organization LABORSTA Economically Active Population, Estimates and Projections, October 2011. International Labour Organization (ILO), 2011.

5. Office for National Statistics (United Kingdom). Nomis Official Labor Market Statistics - Annual Population Survey. Newport, United Kingdom: Office for National Statistics (United Kingdom).

6. National Bureau of Statistics of China. China 1% National Population Sample Survey 1995. Ann Arbor, United States: China Data Center, University of Michigan.

7. GBD 2015 Mortality and Causes of Death Collaborators. Global, regional, and national life expectancy, all-cause and cause-specific mortality for 249 causes of death, 1980–2015: a systematic analysis for the Global Burden of Disease Study 2015. *Lancet Rev*.

8. Wilson DH, Walsh PG, Sanchez L, *et al.* The epidemiology of hearing impairment in an Australian adult population. *Int J Epidemiol* 1999; 28: 247–52

9. Kauppinen T, Toikkanen J, Pederson D, Young R, Kogevinas M, Ahrens W, et al. Occupational Exposure to Carcinogens in the European Union in 1990-93. Helsinki, Finland: Finnish Institute of Occupational Health; 1998.

10. Kauppinen T, Toikkanen J, Pedersen D, Young R, Ahrens W, Boffetta P, et al. Occupational exposure to carcinogens in the European Union. *Occup Environ Med* 2000; 57(1): 10–18.

11. Driscoll T, et al. The global burden of non-malignant respiratory disease due to occupational airborne exposures. *American Journal of Industrial Medicine 2005; 48*(6): 432-445.

12. Nelson, D. I., Concha-Barrientos, M., Driscoll, T., Steenland, K., Fingerhut, M., Punnett, L. & Corvalan, C. (2005). The global burden of selected occupational diseases and injury risks: Methodology and summary. *American journal of industrial medicine*, *48*(6), 400-418

13. Lin R-T, Takahashi K, Karjalainen A, *et al.* Ecological association between asbestos-related diseases and historical asbestos consumption: an international analysis. *Lancet* 2007; **369**: 844–9.

14. Goodman M, Morgan RW, Ray R, Malloy CD, Zhao K. Cancer in asbestos-exposed occupational cohorts: a meta-analysis. *Cancer Causes Control* 1999; **10**: 453–65.

Table 1 – Exposure rate by economic activity (per 100,000 workers)

Risk factor	Agriculture, hunting, forestry and fishing	Mining and quarrying	Manufacturing	Electricity, gas, and water	Construction	Wholesale and retail trade and restaurants and hotels	Transport, storage, and communication	Financing, insurance, real estate, and business services	Community, social, and personal services
Arsenic	54	72	399	148	134	6	-	2	11
Asbestos	1,248	10,248	589	1,702	5,203	292	684	16	286
Benzene	59	197	308	91	75	1,037	520	41	2,330
Beryllium	-	55	207	70	4	2	11	-	3
Cadmium	-	-	486	287	291	2	65	-	48
Chromium VI	-	346	2,061	409	237	17	369	-	227
Diesel engine exhaust	646	21,970	1,192	3,359	5,816	485	13,432	-	920
Secondhand smoke	2,082	163	5,249	6,172	4,830	9,278	6,965	4,584	3,633
Formaldehyde	186	255	2,103	28	545	53	23	22	594
Nickel	-	2,025	1,663	352	47	7	3	-	43
Polycyclic aromatic hydrocarbons	-	1,021	1,650	3,066	1,328	106	905	-	388
Silica	372	23,049	2,316	1,415	18,860	17	476	2	60
Sulfuric acid	-	366	1,488	928	577	264	255	81	189
Noise, 90+ dB, high exposure	26,100	57,200	23,300	27,400	36,200	100	18,000	400	15,900
Noise, 85-90 dB, high exposure	16,700	25,400	32,200	13,800	21,000	23,100	28,700	23,000	17,600
Noise, 90+ dB, low exposure	18,000	39,300	10,600	20,400	25,100	0	7,900	0	900
Noise, 85-90 dB, low exposure	14,400	29,400	24,500	12,300	19,400	1,800	20,200	3,100	13,100
Particulates, developed, high exposure	10,000	10,000	10,000	10,000	10,000	0	10,000	0	0
Particulates, developed, low exposure	5,000	7,000	7,000	5,000	7,000	500	5,000	500	500
Particulates, developing, high exposure	10,000	40,000	40,000	10,000	40,000	0	10,000	0	0
Particulates, developing, low exposure	70,000	40,000	40,000	70,000	40,000	10,000	70,000	10,000	10,000

Ozone risk factor estimation process

Ambient ozone

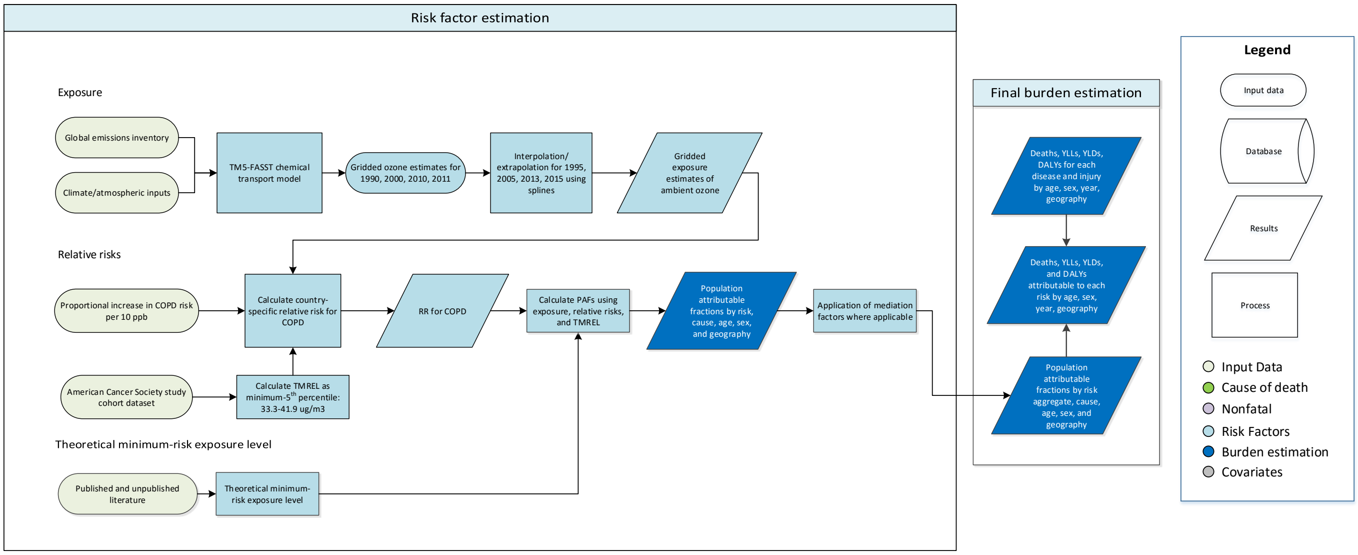

Input data and methodological summary

Exposure

Case definition

For GBD 2015, exposure to ozone pollution is defined as the number of parts-per-billion (ppb) of ozone (O^3).

Input data

Data for estimating ozone exposure is derived from the TM5-FASST chemical transport model, which generates a three-month running average of daily one-hour maximum ozone values at the 0.1° × 0.1° level for the years 1990, 2000, and 2010.[1]

Modelling strategy

The process for modelling ozone exposure has remained stable since GBD 2010 and GBD 2013. Natural cubic splines were used to interpolate for the years 1995, 2005, and 2011. Annualised rate of change was used to predict for the years 2013 and 2015. The uncertainty for exposure at the grid level was assumed to be ±6% of the estimated concentration, in accordance with previous work. Uncertainty for ozone was calculated by assuming a +/- 6% uncertainty interval around the estimation concentration.

Theoretical minimum-risk exposure level

The TMREL of ozone was defined based on the exposure distribution from American Cancer Society CPS-II study, which was the source of the GBD 2015 ozone mortality RR estimate. As with $PM_{2.5}$, a uniform

distribution was drawn around the minimum and fifth percentile values experienced by the cohort. This value was not updated for GBD 2015, and continues to be defined as ~U(33.3, 41.9), in ppb.

No other significant changes were made from GBD 2013 to GBD 2015.

Relative risks

The relative risk of ozone exposure for respiratory COPD was extracted from literature and was not updated for GBD 2015. The relative risk is applied linearly per 10 ppb of ozone exposure and is defined as 1.029 (1.010–1048).[2]

References

1. Brauer M, et al. Ambient Air Pollution Exposure Estimation for the Global Burden of Disease 2013. Environ Sci Technol 2016; 50: 79-88.
2. Jerrett M, Burnett RT, Pope CA, et al. Long-term ozone exposure and mortality. N Engl J Med 2009; 360: 1085–95.

Secondhand smoke risk factor estimation process

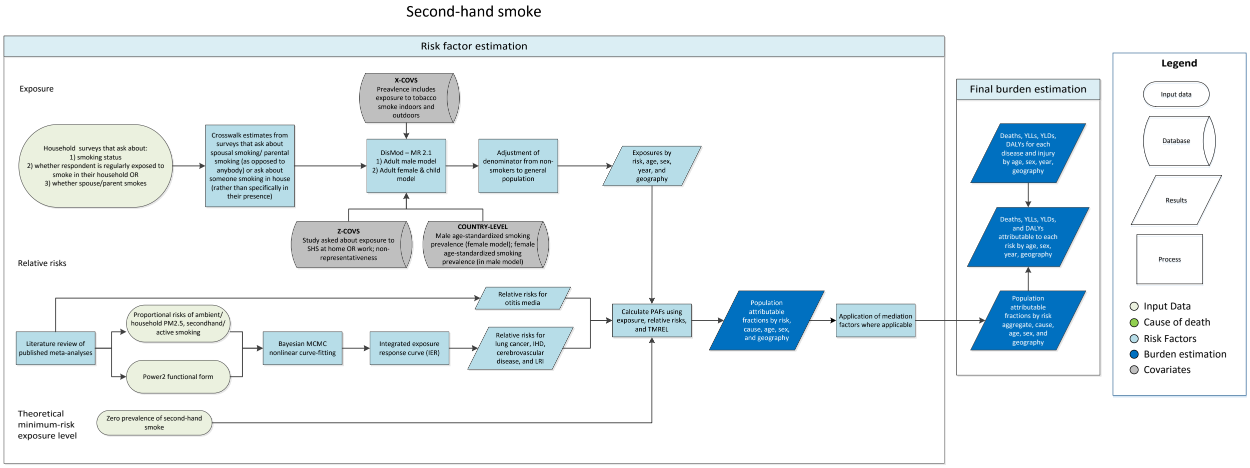

Input data and methodological summary

Exposure

Case definition

We measure exposure to any tobacco smoke inside the home among non-smokers. Ex-smokers and occasional smokers are considered non-smokers for the purposes of this analysis. Exposure was evaluated for both children and adults.

Input data

We included surveys that had at least one question about smoking status and also asked about either exposure to tobacco smoke inside the home, whether or not the respondent lives with any smokers or whether their spouse smokes. For children we also used surveys that asked about parental smoking. Some main sources include Global Adult Tobacco Survey (GATS), Global Youth Tobacco Survey (GYTS), DHS, NHANES, BRFSS, Eurobarometer, etc.

Updates to systematic reviews are performed on an ongoing schedule across all GBD causes and risk factors, an update for second-hand smoke will be performed in the next one to two iterations.

Many new surveys were added for GBD 2015, which were identified and accessed using GHDx. We cross-referenced with available sources used for smoking in order to evaluate whether these sources were also useful for secondhand smoke. Some of the big new survey series that were added included the National Adult Tobacco Survey and National Youth Tobacco survey series from the U.S., VIGITEL and Risk Factor Chronic Disease Surveillance data from Brazil, and the Chronic Disease Risk Factor Surveillance from China. All new Global Youth Tobacco Surveys (GYTS), Global Adult Tobacco Surveys (GATS), Global school-based student health surveys (GSHS) and Eurobarometer were added as well, in addition to other one-off surveys that evaluated secondhand smoke in the household.

Modelling strategy

We used the traditional PAF equation to estimate burden based on exposure and relative risks. Prevalence of secondhand smoke exposure among nonsmokers is modelled in Dismod-MR and all crosswalks/adjustments are done both within and outside of DisMod to account for alternative case definitions.

In GBD 2015, a new modelling change we implemented was to crosswalk surveys asking about spousal smoking or parental smoking (depending on adults versus children) to our gold standard of any exposure to secondhand smoke in the household by anyone. A sizable group of the DHS surveys do not ask directly about smoke exposure in the household, and thus exposure is ascertained indirectly through looking at the smoking status of each partner in the couple's module to see if there is a "mixed-status" relationship in which one partner is exposed to the other's smoke.

Another adjustment that we made prior to DisMod was for the act of smoking. In some surveys, such as the Global Youth Tobacco Survey, the survey only asks whether their parent smokes, not whether the child being interviewed is actively exposed to smoke on a regular basis (which we define as at least once a week). Thus, in addition to adjusting for spouse/parent versus anybody, we also adjusted for whether the survey asked the person whether they were directly exposed to smoke or just whether people smoked who lived in their home. The two-by-two table below helps illustrate the different potential combinations of alternate definitions that we adjusted for.

	Spouse/parent	Anybody
Act of smoking	A	B
Non-act	C	D

We used a mixed effects regression to crosswalk these alternative definitions, with interactions between anybody smoking and sex, fixed effects on act of smoking, and nested random effects at the super-region, region, and country levels. Previously, this crosswalk was done in DisMod.

Once we had crosswalked these alternative definitions, we modelled secondhand smoke prevalence as a single parameter prevalence model in DisMod-MR. Another modelling change that we made in GBD 2015 was to run separate models for male and female secondhand smoke exposure, with children included in the female model. This decision was made because the sex effect being estimated with the combined gender model was underestimating the sizably higher impact of secondhand smoke on women as compared to men. Thus, we decided to model them separately.

In the female model, we used with age mesh points at 0 5 10 15 18 20 30 40 50 60 80 & 100, while in the male model we used age mesh points at 0 15 18 20 30 40 50 60 80 & 100. The difference in age mesh points was due to the fact that all children were modelled as female due to similar rates of exposure, while the male model was limited to adult males greater than 15.

We use the age-standardised smoking prevalence among females as a country-level covariate in the male model, and the age-standardised smoking prevalence among males as a country-level covariate in the female model. This was a modelling change from GBD 2013, in which we only had one secondhand smoke

model and used the age-standardised smoking prevalence rate among men. In addition, we used one study-level fixed effects to account for the different case definitions in our dataset:

- Study-level fixed effects on integrand value (x-cov)
    - Prevalence figure includes exposure to tobacco smoke outdoors as well as indoors
- Study-level fixed effects on integrand variance (z-cov)
    - Study asked about exposure to secondhand smoke at home and/or work (rather than exposure inside the home only)
    - Study was not nationally representative

All raw input CSA data points had a measure of uncertainty going into DisMod – standard error, confidence interval, or effective sample size – and the uncertainty around final estimates also takes into account uncertainty from study-level covariate fixed effects on variance, as well as geographic random effects.

Theoretical minimum-risk exposure level

The theoretical minimum-risk exposure level for secondhand smoke is zero exposure among non-smokers to secondhand smoke in the home.

Relative risks

For children under 5 years of age, we estimate the burden of lower respiratory infections (LRI) and otitis media attributable to secondhand smoke exposure. For adults greater or equal to 25 years of age we estimate the burden of lung cancer, ischemic heart disease, cerebrovascular disease, and lower respiratory infections (LRI) attributable to secondhand smoke exposure. For GBD 2010 all of these pooled relative risks came from published meta-analyses, but for GBD 2015 we used country-specific relative risks that were created using integrated exposure response curves (IER). The relative risk for otitis media still comes from a published meta-analysis, as opposed to the IER approach.

Appendix Table 2. GATHER checklist of information that should be included in reports of global health estimates, with description of compliance and location of information for GBD 2015 paper on COPD and asthma

#	GATHER checklist item	Description of compliance	Reference
**Objectives and funding**			
1	Define the indicators, populations, and time periods for which estimates were made.	Narrative provided in paper and appendix describing indicators, definitions, and populations.	Manuscript; methods appendix, Section 1. GBD Overview
2	List the funding sources for the work.	Funding sources listed in paper.	Funding of GBD by Bill & Melinda Gates Foundation acknowledged
**Data Inputs**			
For all data inputs from multiple sources that are synthesised as part of the study:			
3	Describe how the data were identified and how the data were accessed.	Narrative description of data-seeking methodology provided.	• Main text, methods<br>• Appendix, description of methods of estimating fatal and non-fatal outcomes of COPD and asthma
4	Specify the inclusion and exclusion criteria. Identify all ad-hoc exclusions.	Narrative about inclusion and exclusion criteria provided.	• Main text, methods<br>• Appendix, description of methods of estimating fatal and non-fatal outcomes of COPD and asthma
5	Provide information on all included data sources and their main characteristics. For each data source used, report reference information or contact name/institution, population represented, data collection method, year(s) of data collection, sex and age range, diagnostic criteria or measurement method, and sample size, as relevant.	Interactive, online data source tool that provides metadata for data sources by component, geography, cause, risk, or impairment has been developed.	http://ghdx.healthdata.org/gbd-2015/data-input-sources
6	Identify and describe any categories of input data that have potentially important biases (e.g., based on characteristics listed in item 5).	Summary of known biases by cause included in appendix.	• Main text, methods, and discussion<br>• Appendix, description of methods of estimating fatal and non-fatal outcomes of COPD and asthma
For data inputs that contribute to the analysis but were not synthesised as part of the study:			
7	Describe and give sources for any other data inputs.	Included in online data source tool.	http://ghdx.healthdata.org/gbd-2015/data-input-sources
For all data inputs:			

8	Provide all data inputs in a file format from which data can be efficiently extracted (eg, a spreadsheet as opposed to a PDF), including all relevant meta-data listed in item 5. For any data inputs that cannot be shared due to ethical or legal reasons, such as third-party ownership, provide a contact name or the name of the institution that retains the right to the data.	Downloads of input data available through online tools, including data visualization tools and data query tools. Input data not available in tools will be made available upon request.	Online data tools http://ghdx.healthdata.org/gbd-2015/data-input-sources http://www.healthdata.org/results/data-visualizations; http://ghdx.healthdata.org/; http://ghdx.healthdata.org/gbd-data-tool
**Data analysis**			
9	Provide a conceptual overview of the data analysis method. A diagram may be helpful.	Flow diagrams and narrative of methodological processes have been provided.	• Main text, methods<br>• Appendix description of methods of estimating fatal and non-fatal outcomes of COPD and asthma
10	Provide a detailed description of all steps of the analysis, including mathematical formulae. This description should cover, as relevant, data cleaning, data pre-processing, data adjustments and weighting of data sources, and mathematical or statistical model(s).	Flow diagrams and narrative of methodological processes have been provided.	• Main text, methods<br>• Appendix description of methods of estimating fatal and non-fatal outcomes of COPD and asthma
11	Describe how candidate models were evaluated and how the final model(s) were selected.	Provided in the methodological write-ups.	• Appendix description of methods of estimating fatal and non-fatal outcomes of COPD and asthma
12	Provide the results of an evaluation of model performance, if done, as well as the results of any relevant sensitivity analysis.	Provided in the methodological write-ups.	Appendix description of methods of estimating fatal and non-fatal outcomes of COPD and asthma
13	Describe methods for calculating uncertainty of the estimates. State which sources of uncertainty were, and were not, accounted for in the uncertainty analysis.	Provided in the methodological write-ups.	• Main text, methods<br>• Appendix description of methods of estimating fatal and non-fatal outcomes of COPD and asthma
14	State how analytic or statistical source code used to generate estimates can be accessed.	Access statement provided.	http://ghdx.healthdata.org/gbd-2015-code
**Results and Discussion**			
15	Provide published estimates in a file format from which data can be efficiently extracted.	GBD 2015 results are available through online data visualization tools, the	Online data tools http://www.healthdata.org/results/data-visualizations;

		Global Health Data Exchange, and the online data query tool	http://ghdx.healthdata.org/; http://ghdx.healthdata.org/gbd-data-tool
16	Report a quantitative measure of the uncertainty of the estimates (eg, uncertainty intervals).	Uncertainty intervals are provided with all results.	Main text and online data tools (to go live with GBD 2015 at publication) http://www.healthdata.org/results/data-visualizations; http://ghdx.healthdata.org/; http://ghdx.healthdata.org/gbd-data-tool
17	Interpret results in light of existing evidence. If updating a previous set of estimates, describe the reasons for changes in estimates.	Discussion of methodological changes between GBD rounds provided in the narrative of the paper.	Main text, discussion
18	Discuss limitations of the estimates. Include a discussion of any modelling assumptions or data limitations that affect interpretation of the estimates.	Discussion of limitations provided in the narrative of the main paper as well as in the methodological write-ups in the appendix.	• Main text, discussion<br>• Appendix description of methods of estimating fatal and non-fatal outcomes of COPD and asthma

Appendix Table 3. DALYs caused by COPD and asthma in 2015 and percent change in all-age numbers and age-standardised rates between 1990 and 2015, by location						
	COPD			Asthma		
	DALYs	Change in DALY numbers from 1990 to 2015 (percent)	Change in DALYs from 1990 to 2015, age-standardised rates (percent)	DALYs	Change in DALY numbers from 1990 to 2015 (percent)	Change in DALYs from 1990 to 2015, age-standardised rates (percent)
**Global**	63,850,433 (61,215,316 to 66,288,554)	-1.0 (-7.1 to 6.2)	-43.7 (-47.0 to -39.8)	26,168,785 (20,501,407 to 32,582,992)	-14.6 (-26.0 to 2.1)	-42.8 (-52.0 to -29.5)
**High-income**	7,178,960 (6,860,198 to 7,523,479)	26.9 (23.8 to 31.6)	-20.1 (-22.1 to -17.0)	2,987,889 (2,050,480 to 4,023,604)	-18.6 (-22.6 to -15.2)	-31.2 (-34.7 to -28.3)
**High-income North America**	3,207,917 (3,055,194 to 3,350,917)	50.3 (45.9 to 55.0)	-3.6 (-6.3 to -0.6)	886,295 (616,105 to 1,190,056)	4.9 (-3.1 to 12.6)	-19.6 (-26.2 to -13.3)
Canada	175,370 (162,732 to 190,729)	36.7 (26.7 to 48.5)	-24.0 (-29.4 to -17.7)	93,562 (62,626 to 128,549)	4.5 (-4.6 to 14.9)	-19.2 (-26.8 to -11.1)
Greenland	416 (380 to 461)	11.1 (-0.4 to 23.7)	-35.8 (-42.3 to -28.5)	169 (119 to 224)	-20.8 (-28.4 to -14.1)	-33.5 (-41.7 to -26.9)
United States	3,031,070 (2,885,010 to 3,170,315)	51.1 (46.6 to 56.2)	-1.8 (-4.7 to 1.3)	792,258 (553,055 to 1,063,564)	4.9 (-3.4 to 13.4)	-19.6 (-26.5 to -12.9)
**Australasia**	147,393 (138,813 to 158,093)	9.5 (3.2 to 17.8)	-39.5 (-43.1 to -34.9)	128,635 (85,844 to 180,364)	-12.4 (-19.1 to -6.1)	-37.1 (-42.1 to -32.1)
Australia	118,336 (110,837 to 127,702)	7.9 (0.7 to 17.4)	-41.1 (-45.0 to -36.1)	106,989 (71,805 to 150,340)	-11.2 (-18.6 to -3.9)	-36.9 (-42.4 to -31.4)
New Zealand	29,057 (27,312 to 30,953)	16.6 (9.4 to 25.0)	-32.2 (-36.3 to -27.5)	21,647 (14,367 to 30,038)	-18.0 (-25.3 to -10.2)	-37.5 (-43.4 to -31.0)
**High-income Asia Pacific**	726,460 (674,245 to 790,533)	29.2 (22.8 to 39.5)	-36.6 (-40.0 to -31.8)	400,646 (279,419 to 540,325)	-44.3 (-48.9 to -40.1)	-53.4 (-58.1 to -49.2)
Brunei	1,883 (1,736 to 2,021)	47.7 (33.9 to 64.5)	-36.1 (-42.0 to -28.0)	1,657 (1,230 to 2,165)	25.3 (13.4 to 36.5)	-28.4 (-37.1 to -20.6)
Japan	535,770 (495,537 to 587,585)	23.2 (16.4 to 33.8)	-37.0 (-40.3 to -32.5)	267,851 (182,286 to 368,598)	-46.5 (-51.2 to -41.8)	-52.7 (-57.0 to -48.2)
Singapore	9,900 (9,156 to 10,581)	-27.1 (-32.2 to -21.2)	-72.2 (-74.2 to -69.9)	8,924 (6,080 to 12,099)	-30.1 (-39.6 to -22.0)	-55.8 (-62.4 to -50.2)
South Korea	178,907 (164,112 to 193,727)	59.2 (45.1 to 75.3)	-37.3 (-43.1 to -30.2)	122,214 (89,178 to 160,396)	-40.3 (-46.8 to -34.1)	-65.3 (-71.3 to -59.6)
**Western Europe**	2,725,245 (2,600,747 to 2,867,656)	5.9 (2.6 to 10.3)	-29.0 (-31.2 to -25.9)	1,348,291 (909,929 to 1,838,168)	-22.8 (-26.8 to -19.6)	-30.0 (-33.9 to -26.8)
Andorra	395 (338 to 461)	72.8 (46.8 to 104.0)	-20.3 (-31.4 to -7.4)	235 (155 to 327)	28.1 (16.3 to 40.0)	-18.8 (-26.6 to -11.1)
Austria	47,670 (44,506 to 51,123)	24.3 (16.6 to 32.4)	-11.7 (-16.9 to -6.1)	25,268 (16,785 to 34,868)	-27.0 (-35.0 to -20.3)	-32.1 (-39.8 to -24.9)
Belgium	91,554 (85,839 to 97,385)	6.1 (-0.6 to 13.6)	-23.8 (-28.6 to -18.5)	28,987 (19,894 to 39,533)	-35.4 (-40.3 to -30.5)	-42.2 (-46.8 to -37.7)
Cyprus	2,863 (2,629 to 3,116)	-12.5 (-21.4 to -2.4)	-52.0 (-56.8 to -46.4)	3,432 (2,404 to 4,659)	-3.8 (-11.7 to 4.0)	-31.0 (-36.9 to -25.3)
Denmark	58,476 (55,013 to 62,188)	12.5 (5.7 to 20.1)	-15.1 (-20.3 to -9.6)	15,597 (10,510 to 21,566)	-20.9 (-30.8 to -10.2)	-27.9 (-36.8 to -17.4)
Finland	25,050 (22,760 to 27,720)	10.8 (2.6 to 20.3)	-27.8 (-33.0 to -21.8)	21,232 (14,163 to 29,057)	3.6 (-6.5 to 15.0)	-8.2 (-18.9 to 1.7)
France	199,264 (183,435 to 216,245)	-17.5 (-23.8 to -10.9)	-46.4 (-50.3 to -42.3)	201,242 (135,844 to 279,186)	-26.2 (-31.9 to -20.6)	-35.9 (-41.5 to -30.5)
Germany	639,550 (602,240 to 678,297)	8.8 (2.5 to 15.4)	-25.0 (-29.3 to -20.4)	224,765 (154,757 to 306,679)	-41.4 (-47.2 to -36.7)	-44.5 (-49.9 to -39.4)
Greece	88,653 (82,805 to 95,107)	55.5 (46.2 to 64.6)	-1.3 (-6.8 to 3.9)	26,463 (17,478 to 36,407)	2.4 (-5.4 to 11.7)	-1.5 (-9.2 to 9.4)
Iceland	1,300 (1,194 to 1,412)	21.4 (13.2 to 29.8)	-26.0 (-30.9 to -20.9)	942 (622 to 1,292)	-6.7 (-13.7 to 1.0)	-27.2 (-33.2 to -21.1)
Ireland	23,077 (21,081 to 25,465)	-17.7 (-26.3 to -7.7)	-45.8 (-51.4 to -39.3)	18,024 (11,981 to 25,385)	-20.2 (-26.7 to -14.1)	-38.5 (-43.6 to -33.5)
Israel	24,457 (22,661 to 26,229)	42.1 (31.8 to 51.8)	-36.4 (-41.0 to -31.8)	22,185 (15,124 to 30,656)	12.6 (3.9 to 22.4)	-38.1 (-43.3 to -32.5)
Italy	333,073 (309,587 to 358,588)	6.8 (-1.2 to 16.6)	-35.0 (-39.5 to -29.2)	108,372 (71,741 to 150,467)	-30.6 (-39.3 to -23.5)	-33.6 (-43.0 to -25.3)
Luxembourg	2,730 (2,528 to 2,921)	1.9 (-5.1 to 8.9)	-35.4 (-39.7 to -31.0)	2,182 (1,476 to 3,003)	-4.4 (-12.7 to 3.3)	-33.5 (-39.5 to -28.0)
Malta	1,780 (1,634 to 1,948)	24.4 (14.1 to 36.8)	-35.9 (-41.3 to -29.5)	1,458 (963 to 2,019)	-11.4 (-20.2 to -2.5)	-24.6 (-32.6 to -16.4)
Netherlands	138,162 (129,168 to 148,032)	29.2 (21.0 to 38.3)	-14.9 (-20.1 to -9.0)	53,059 (34,741 to 73,952)	-2.3 (-9.1 to 5.9)	-14.3 (-20.3 to -7.0)
Norway	35,016 (32,722 to 37,513)	67.9 (56.6 to 80.2)	31.6 (23.2 to 40.9)	15,049 (10,218 to 20,678)	-29.9 (-37.5 to -22.1)	-39.1 (-45.5 to -31.3)
Portugal	66,726 (62,689 to 71,251)	1.2 (-5.0 to 8.2)	-34.8 (-38.8 to -30.2)	38,931 (25,937 to 53,847)	-21.8 (-29.1 to -14.2)	-26.1 (-33.7 to -17.3)
Spain	288,908 (270,702 to 308,065)	3.9 (-2.9 to 11.9)	-39.7 (-43.6 to -35.1)	153,197 (104,073 to 209,254)	-0.4 (-7.4 to 6.4)	-18.0 (-24.3 to -11.2)
Sweden	68,114 (62,490 to 74,107)	30.2 (23.2 to 37.6)	0.6 (-4.5 to 5.9)	26,620 (17,965 to 36,843)	-25.2 (-31.0 to -19.8)	-30.3 (-35.8 to -24.4)
Switzerland	37,351 (34,420 to 40,468)	4.7 (-2.9 to 13.2)	-30.1 (-35.2 to -24.7)	22,759 (14,997 to 31,673)	-13.5 (-23.3 to -2.8)	-27.3 (-35.8 to -18.2)
United Kingdom	548,554 (520,273 to 588,796)	-2.2 (-6.7 to 5.5)	-24.6 (-28.1 to -18.7)	336,960 (226,342 to 467,528)	-14.5 (-18.0 to -11.6)	-21.9 (-24.9 to -19.4)

	COPD			Asthma		
	DALYs	Change in DALY numbers from 1990 to 2015 (percent)	Change in DALYs from 1990 to 2015, age-standardised rates (percent)	DALYs	Change in DALY numbers from 1990 to 2015 (percent)	Change in DALYs from 1990 to 2015, age-standardised rates (percent)
England	447,608 (423,936 to 480,199)	-3.9 (-8.5 to 3.7)	-25.9 (-29.4 to -20.0)	271,853 (182,230 to 377,480)	-15.9 (-19.2 to -13.0)	-23.8 (-26.7 to -21.4)
Northern Ireland	14,703 (13,494 to 16,337)	15.6 (6.0 to 28.6)	-19.7 (-26.4 to -10.7)	8,156 (5,466 to 11,316)	-12.9 (-18.9 to -7.1)	-23.2 (-28.2 to -18.0)
Scotland	55,298 (51,528 to 59,784)	6.0 (-2.0 to 15.9)	-17.6 (-23.5 to -9.9)	34,201 (22,892 to 47,275)	-9.0 (-15.8 to -1.9)	-10.8 (-17.8 to -3.7)
Wales	30,945 (28,657 to 33,603)	3.0 (-5.2 to 13.7)	-19.3 (-25.6 to -11.3)	22,751 (15,261 to 31,905)	-5.5 (-11.1 to 0.5)	-9.2 (-15.1 to -3.1)
**Southern Latin America**	371,945 (353,118 to 391,691)	49.3 (41.4 to 57.8)	-10.6 (-15.3 to -5.5)	224,021 (152,853 to 305,997)	6.0 (-1.3 to 12.2)	-19.6 (-25.6 to -14.2)
Argentina	273,890 (258,275 to 289,737)	51.9 (42.3 to 61.5)	-2.6 (-8.7 to 3.5)	148,977 (102,022 to 202,719)	0.7 (-6.6 to 8.5)	-22.8 (-28.7 to -16.5)
Chile	69,386 (63,069 to 78,244)	38.3 (24.9 to 56.1)	-34.2 (-40.6 to -26.1)	61,203 (40,902 to 84,460)	33.1 (17.0 to 48.7)	-5.7 (-17.7 to 4.6)
Uruguay	28,643 (27,066 to 30,242)	54.1 (43.3 to 66.4)	14.9 (6.9 to 24.2)	13,830 (9,878 to 18,443)	-20.9 (-27.9 to -14.7)	-29.1 (-35.8 to -23.2)
**Central Europe, Eastern Europe, and Central Asia**	2,235,951 (2,138,230 to 2,324,989)	-25.6 (-28.6 to -23.0)	-42.7 (-45.0 to -40.9)	910,592 (655,937 to 1,197,029)	-43.4 (-48.5 to -38.6)	-45.0 (-50.9 to -39.7)
**Eastern Europe**	1,171,686 (1,106,306 to 1,235,865)	-34.0 (-38.0 to -30.5)	-47.1 (-50.1 to -44.4)	444,733 (317,326 to 582,638)	-56.2 (-61.3 to -51.2)	-54.4 (-60.1 to -49.1)
Belarus	68,958 (63,214 to 74,961)	-39.8 (-45.4 to -33.7)	-50.7 (-55.2 to -45.9)	27,615 (20,100 to 36,382)	-44.9 (-52.9 to -37.6)	-42.4 (-51.0 to -34.3)
Estonia	3,854 (3,554 to 4,189)	-34.2 (-39.8 to -28.5)	-44.4 (-49.1 to -39.7)	3,427 (2,440 to 4,584)	-47.9 (-54.6 to -41.8)	-40.9 (-49.0 to -33.5)
Latvia	6,642 (6,131 to 7,235)	-48.7 (-53.8 to -43.5)	-52.7 (-57.4 to -48.0)	5,321 (3,699 to 7,146)	-57.0 (-62.8 to -51.6)	-47.7 (-55.0 to -40.2)
Lithuania	14,280 (13,354 to 15,257)	-48.4 (-51.9 to -44.7)	-56.9 (-59.7 to -53.6)	6,403 (4,353 to 8,697)	-43.5 (-50.2 to -37.8)	-34.5 (-42.6 to -27.4)
Moldova	20,163 (18,969 to 21,409)	-48.9 (-52.3 to -45.1)	-56.6 (-59.4 to -53.6)	7,357 (5,077 to 10,088)	-40.5 (-47.6 to -33.3)	-36.9 (-45.2 to -28.7)
Russia	759,408 (706,800 to 813,347)	-24.6 (-30.3 to -19.3)	-41.9 (-45.9 to -38.0)	256,622 (181,742 to 341,235)	-56.9 (-61.8 to -51.7)	-56.3 (-61.9 to -50.4)
Ukraine	298,382 (274,576 to 323,798)	-47.3 (-52.7 to -41.8)	-53.3 (-57.7 to -48.4)	137,989 (96,977 to 181,771)	-57.7 (-64.9 to -50.0)	-52.4 (-60.6 to -44.6)
**Central Europe**	647,610 (621,777 to 672,773)	-18.0 (-20.7 to -15.2)	-40.0 (-41.9 to -37.9)	234,117 (163,257 to 317,640)	-34.3 (-39.9 to -29.2)	-31.9 (-38.5 to -25.9)
Albania	9,269 (8,307 to 10,261)	-25.8 (-35.9 to -15.3)	-54.3 (-59.7 to -48.2)	6,619 (5,020 to 8,468)	-22.3 (-30.6 to -13.1)	-29.9 (-39.3 to -21.6)
Bosnia and Herzegovina	17,007 (15,296 to 18,806)	-17.6 (-27.0 to -6.9)	-49.2 (-54.9 to -42.7)	6,108 (4,325 to 8,150)	-16.4 (-26.4 to -6.0)	-13.1 (-25.9 to -1.4)
Bulgaria	54,124 (51,033 to 57,307)	-8.5 (-14.5 to -2.2)	-20.2 (-25.5 to -15.0)	12,984 (8,787 to 17,859)	-31.5 (-39.0 to -24.2)	-13.6 (-23.0 to -3.9)
Croatia	26,014 (24,468 to 27,664)	22.1 (13.9 to 30.9)	-7.5 (-13.5 to -1.1)	8,382 (5,833 to 11,458)	-36.3 (-43.0 to -30.6)	-28.6 (-36.2 to -21.5)
Czech Republic	51,221 (47,927 to 55,058)	-5.2 (-11.8 to 2.6)	-32.4 (-36.9 to -26.8)	15,968 (10,965 to 21,607)	-34.6 (-40.7 to -29.2)	-35.3 (-41.7 to -29.9)
Hungary	85,505 (80,852 to 90,407)	-11.6 (-16.9 to -5.7)	-27.8 (-31.9 to -23.0)	17,044 (11,823 to 22,896)	-37.8 (-43.3 to -32.4)	-35.2 (-41.3 to -28.8)
Macedonia	7,671 (7,080 to 8,282)	-7.7 (-15.5 to 0.8)	-42.3 (-47.2 to -36.8)	4,776 (3,463 to 6,333)	-15.6 (-24.4 to -6.8)	-29.4 (-38.4 to -21.1)
Montenegro	1,078 (941 to 1,224)	31.5 (13.7 to 52.5)	-12.2 (-23.8 to 2.1)	994 (662 to 1,372)	9.1 (-0.4 to 19.7)	3.9 (-4.5 to 13.3)
Poland	199,135 (187,543 to 210,887)	-13.2 (-19.1 to -7.1)	-41.0 (-44.8 to -37.0)	86,256 (59,157 to 117,697)	-38.9 (-46.8 to -30.6)	-40.8 (-49.3 to -32.2)
Romania	119,465 (109,349 to 128,144)	-44.2 (-48.5 to -39.8)	-55.2 (-58.7 to -51.6)	40,924 (28,043 to 55,471)	-33.3 (-40.0 to -27.5)	-22.8 (-31.3 to -14.7)
Serbia	50,527 (47,436 to 53,640)	10.7 (1.4 to 20.8)	-21.8 (-28.0 to -14.8)	22,144 (15,885 to 29,213)	-32.4 (-37.2 to -27.4)	-33.4 (-38.4 to -28.3)
Slovakia	17,961 (16,688 to 19,240)	6.3 (-1.4 to 14.8)	-24.2 (-29.7 to -18.3)	8,929 (6,035 to 12,079)	-14.1 (-22.9 to -5.4)	-17.0 (-25.6 to -8.0)
Slovenia	8,633 (7,993 to 9,331)	-16.6 (-22.8 to -9.5)	-50.5 (-54.1 to -46.4)	2,989 (2,019 to 4,010)	-29.1 (-36.0 to -22.5)	-32.0 (-39.7 to -25.4)
**Central Asia**	416,655 (396,832 to 437,552)	-5.8 (-10.8 to -0.5)	-36.4 (-39.6 to -33.0)	231,742 (171,921 to 301,714)	-2.3 (-11.1 to 5.1)	-29.6 (-37.5 to -23.1)
Armenia	17,957 (16,441 to 19,606)	-24.0 (-30.8 to -16.8)	-46.2 (-51.0 to -41.1)	5,641 (3,850 to 7,675)	-17.0 (-23.6 to -10.2)	-11.6 (-18.9 to -5.1)
Azerbaijan	37,777 (33,912 to 42,303)	-7.0 (-17.1 to 4.3)	-51.6 (-56.8 to -46.1)	20,603 (14,279 to 28,099)	15.5 (1.1 to 30.0)	-26.7 (-38.0 to -15.7)
Georgia	30,212 (27,829 to 32,813)	36.0 (22.6 to 51.2)	43.1 (28.5 to 58.1)	9,520 (6,933 to 12,539)	-46.3 (-52.5 to -38.5)	-26.8 (-36.0 to -15.3)
Kazakhstan	132,678 (121,929 to 143,977)	1.6 (-7.8 to 12.8)	-18.7 (-25.9 to -10.1)	50,927 (39,064 to 65,469)	-24.0 (-32.6 to -15.1)	-34.2 (-43.1 to -25.4)
Kyrgyzstan	37,894 (35,220 to 40,603)	-31.1 (-36.9 to -24.7)	-48.3 (-52.6 to -43.8)	12,740 (8,837 to 17,391)	1.7 (-12.6 to 14.1)	-28.9 (-40.2 to -19.5)
Mongolia	7,963 (7,223 to 8,824)	-32.0 (-50.6 to -10.2)	-46.2 (-55.4 to -36.0)	7,241 (5,331 to 9,471)	-4.0 (-17.5 to 8.1)	-34.9 (-45.0 to -24.8)

	COPD			Asthma		
	DALYs	Change in DALY numbers from 1990 to 2015 (percent)	Change in DALYs from 1990 to 2015, age-standardised rates (percent)	DALYs	Change in DALY numbers from 1990 to 2015 (percent)	Change in DALYs from 1990 to 2015, age-standardised rates (percent)
Tajikistan	30,854 (27,505 to 34,352)	-18.0 (-28.1 to -6.6)	-49.9 (-55.9 to -43.1)	18,530 (13,027 to 24,889)	24.5 (7.1 to 41.2)	-25.5 (-38.4 to -13.2)
Turkmenistan	14,794 (13,318 to 16,017)	-31.3 (-37.2 to -24.9)	-64.0 (-66.9 to -60.7)	11,899 (8,037 to 16,117)	-16.3 (-27.7 to -4.4)	-47.7 (-56.5 to -38.9)
Uzbekistan	106,527 (96,928 to 116,377)	7.3 (-4.1 to 20.2)	-43.1 (-49.5 to -36.2)	94,640 (71,354 to 121,039)	20.2 (7.5 to 33.3)	-31.9 (-41.0 to -23.1)
**Latin America and Caribbean**	2,485,103 (2,380,326 to 2,605,007)	31.9 (26.0 to 37.8)	-29.8 (-32.1 to -27.0)	2,161,934 (1,491,815 to 2,932,877)	-5.5 (-11.0 to -0.7)	-27.9 (-32.7 to -24.1)
**Central Latin America**	892,126 (850,400 to 934,854)	25.4 (18.7 to 31.8)	-23.5 (-26.2 to -20.6)	773,568 (536,001 to 1,056,451)	-9.1 (-15.6 to -3.8)	-35.3 (-41.0 to -31.0)
Colombia	208,166 (195,866 to 221,371)	45.2 (35.0 to 56.4)	-20.0 (-24.9 to -14.7)	179,002 (118,827 to 251,648)	10.3 (0.3 to 20.5)	-15.8 (-25.0 to -7.1)
Costa Rica	14,825 (13,709 to 15,878)	88.6 (69.0 to 109.5)	-21.9 (-28.2 to -14.8)	17,513 (11,821 to 24,142)	17.9 (-10.5 to 35.2)	-16.6 (-35.1 to -5.4)
El Salvador	15,281 (13,950 to 16,707)	-56.6 (-67.8 to -39.5)	-59.4 (-66.6 to -50.4)	24,164 (16,509 to 33,187)	-51.2 (-59.7 to -42.8)	-51.9 (-59.4 to -44.2)
Guatemala	32,035 (28,532 to 35,492)	-68.4 (-75.2 to -59.1)	-63.8 (-70.2 to -55.9)	57,693 (40,797 to 78,705)	-22.6 (-37.1 to -8.2)	-51.8 (-60.1 to -43.3)
Honduras	33,111 (26,804 to 40,800)	23.0 (-34.4 to 98.7)	-7.3 (-31.2 to 21.1)	49,535 (36,046 to 65,431)	-27.4 (-42.0 to -10.9)	-41.1 (-51.2 to -31.0)
Mexico	469,304 (443,468 to 493,320)	38.3 (32.4 to 44.7)	-20.1 (-23.4 to -16.9)	306,625 (212,416 to 419,730)	-7.7 (-12.3 to -3.7)	-38.1 (-43.1 to -34.3)
Nicaragua	13,904 (12,654 to 15,150)	53.6 (1.0 to 120.2)	14.0 (-5.3 to 33.0)	19,259 (13,447 to 26,259)	-32.2 (-42.6 to -21.5)	-41.6 (-49.2 to -34.2)
Panama	11,687 (10,415 to 13,115)	28.7 (7.2 to 53.5)	-31.6 (-40.0 to -21.9)	18,470 (12,762 to 25,248)	12.6 (0.7 to 26.3)	-19.8 (-28.2 to -11.2)
Venezuela	93,813 (84,709 to 103,644)	140.3 (115.4 to 167.5)	4.1 (-6.3 to 15.2)	101,307 (69,696 to 140,888)	-3.2 (-12.9 to 6.6)	-32.6 (-38.8 to -26.3)
**Andean Latin America**	135,913 (124,252 to 150,430)	-29.4 (-44.7 to -13.3)	-49.6 (-56.9 to -42.3)	190,735 (126,609 to 267,865)	-24.5 (-37.0 to -14.4)	-39.9 (-49.5 to -32.3)
Bolivia	30,602 (25,928 to 36,580)	1.4 (-18.1 to 26.3)	-48.2 (-58.4 to -35.4)	34,647 (22,804 to 48,872)	23.7 (7.7 to 38.4)	-20.3 (-32.7 to -8.9)
Ecuador	39,210 (35,616 to 43,464)	-38.9 (-52.6 to -18.7)	-54.1 (-61.4 to -45.1)	42,019 (27,548 to 59,408)	-31.7 (-43.6 to -20.1)	-47.3 (-55.6 to -39.2)
Peru	66,102 (58,187 to 74,334)	-32.7 (-52.9 to -6.5)	-47.4 (-58.5 to -34.9)	114,069 (75,313 to 160,832)	-30.1 (-43.6 to -18.5)	-40.6 (-51.4 to -31.9)
**Caribbean**	184,299 (169,828 to 204,311)	36.6 (5.1 to 65.7)	-9.1 (-23.1 to 4.2)	265,706 (192,315 to 351,780)	-11.3 (-21.5 to -1.9)	-26.6 (-34.8 to -19.0)
Antigua and Barbuda	118 (104 to 132)	22.1 (7.1 to 36.6)	-21.5 (-31.5 to -11.6)	378 (253 to 522)	24.5 (10.7 to 38.3)	-13.3 (-23.2 to -3.6)
The Bahamas	765 (678 to 858)	27.4 (11.5 to 46.3)	-45.0 (-51.3 to -37.8)	1,747 (1,209 to 2,368)	3.7 (-7.3 to 14.2)	-26.8 (-34.7 to -19.4)
Barbados	586 (528 to 657)	7.7 (-3.8 to 22.0)	-28.7 (-36.3 to -19.5)	1,289 (921 to 1,725)	-14.6 (-21.6 to -7.5)	-20.9 (-28.2 to -13.0)
Belize	998 (895 to 1,102)	54.4 (29.6 to 81.9)	-7.8 (-18.9 to 4.8)	1,864 (1,282 to 2,583)	37.9 (21.8 to 53.8)	-20.1 (-28.9 to -11.9)
Bermuda	114 (102 to 127)	-23.6 (-32.0 to -13.3)	-51.3 (-56.6 to -45.1)	275 (183 to 387)	-11.1 (-21.7 to 0.4)	-24.8 (-34.0 to -15.2)
Cuba	65,176 (60,674 to 69,752)	102.0 (88.5 to 117.2)	20.9 (13.1 to 30.2)	49,718 (34,776 to 66,670)	-4.1 (-10.9 to 5.9)	-7.0 (-13.5 to 2.6)
Dominica	220 (194 to 253)	22.1 (3.6 to 44.1)	-9.1 (-23.0 to 7.2)	410 (293 to 545)	-11.3 (-19.7 to -2.6)	-12.3 (-21.4 to -3.4)
Dominican Republic	21,733 (19,934 to 23,738)	42.9 (3.5 to 81.2)	-17.2 (-32.6 to -3.4)	55,163 (39,039 to 74,619)	-2.6 (-13.3 to 8.1)	-28.7 (-36.4 to -21.4)
Grenada	315 (288 to 345)	-18.4 (-27.6 to -8.5)	-30.3 (-37.4 to -22.0)	523 (365 to 712)	-9.2 (-18.1 to -1.0)	-11.2 (-20.2 to -3.5)
Guyana	1,888 (1,657 to 2,126)	-2.0 (-17.8 to 13.7)	-21.6 (-31.9 to -10.7)	4,351 (3,158 to 5,855)	-17.5 (-24.9 to -10.2)	-21.2 (-28.1 to -14.3)
Haiti	48,631 (36,294 to 66,520)	-8.5 (-47.5 to 50.5)	-25.5 (-46.8 to 3.3)	96,968 (71,792 to 128,825)	-18.1 (-37.2 to 4.3)	-41.9 (-53.5 to -26.3)
Jamaica	10,000 (8,823 to 11,218)	63.0 (41.6 to 85.6)	12.7 (-1.8 to 28.1)	14,687 (10,359 to 19,783)	-2.5 (-12.7 to 7.7)	-9.9 (-18.8 to -1.0)
Puerto Rico	18,862 (17,396 to 20,712)	42.8 (31.7 to 55.1)	-6.0 (-13.2 to 2.1)	16,184 (11,183 to 21,851)	-22.9 (-30.8 to -14.1)	-23.2 (-32.1 to -13.8)
Saint Lucia	694 (626 to 763)	9.8 (-5.5 to 24.7)	-32.9 (-40.8 to -24.2)	995 (706 to 1,337)	-12.6 (-20.7 to -4.4)	-29.1 (-36.5 to -22.1)
Saint Vincent and the Grenadines	258 (234 to 283)	0.1 (-13.8 to 15.9)	-18.6 (-28.3 to -7.8)	556 (397 to 751)	-19.4 (-26.4 to -12.4)	-13.5 (-21.5 to -6.5)
Suriname	1,394 (1,235 to 1,587)	5.1 (-12.6 to 24.3)	-35.0 (-43.9 to -24.8)	2,804 (1,947 to 3,799)	-5.7 (-15.3 to 3.3)	-26.2 (-34.0 to -19.5)
Trinidad and Tobago	3,476 (3,112 to 3,874)	13.6 (-0.8 to 28.6)	-29.5 (-38.3 to -20.5)	7,559 (5,332 to 10,321)	-23.6 (-29.0 to -18.1)	-27.7 (-34.2 to -22.1)
Virgin Islands, U.S.	363 (328 to 402)	59.5 (41.7 to 78.9)	-22.8 (-30.7 to -13.9)	518 (364 to 692)	-18.4 (-26.3 to -10.3)	-20.8 (-29.9 to -13.4)
**Tropical Latin America**	1,272,765 (1,206,016 to 1,345,092)	50.7 (43.0 to 58.9)	-35.6 (-38.9 to -32.1)	931,925 (635,954 to 1,271,815)	5.3 (-0.4 to 11.0)	-16.0 (-19.3 to -12.5)

	COPD			Asthma		
	DALYs	Change in DALY numbers from 1990 to 2015 (percent)	Change in DALYs from 1990 to 2015, age-standardised rates (percent)	DALYs	Change in DALY numbers from 1990 to 2015 (percent)	Change in DALYs from 1990 to 2015, age-standardised rates (percent)
Brazil	1,252,840 (1,187,098 to 1,324,957)	50.3 (42.8 to 58.6)	-36.1 (-39.4 to -32.5)	910,480 (621,210 to 1,241,327)	5.0 (-0.8 to 10.7)	-15.9 (-19.3 to -12.4)
Paraguay	19,926 (17,368 to 22,879)	73.1 (38.9 to 114.5)	-4.0 (-20.6 to 14.2)	21,445 (14,609 to 29,965)	22.8 (4.6 to 35.6)	-13.9 (-24.2 to -6.1)
**Southeast Asia, East Asia, and Oceania**	20,206,079 (19,085,645 to 21,369,813)	-32.8 (-37.8 to -27.2)	-66.3 (-68.8 to -63.6)	5,807,258 (4,655,499 to 7,135,602)	-7.4 (-17.6 to 3.1)	-35.2 (-44.5 to -26.1)
**East Asia**	15,974,705 (15,049,247 to 16,989,072)	-41.2 (-46.1 to -35.7)	-70.9 (-73.2 to -68.3)	1,918,723 (1,396,238 to 2,490,197)	-31.0 (-40.3 to -23.9)	-47.4 (-55.9 to -40.4)
China	15,389,017 (14,480,887 to 16,349,490)	-42.7 (-47.5 to -37.3)	-71.5 (-73.9 to -69.0)	1,797,438 (1,299,396 to 2,335,122)	-32.8 (-42.1 to -25.7)	-48.5 (-57.1 to -41.4)
North Korea	463,380 (347,829 to 590,756)	89.9 (39.9 to 163.3)	-13.7 (-35.6 to 19.8)	70,130 (46,799 to 103,143)	13.7 (-9.0 to 44.4)	-21.4 (-39.7 to -0.1)
Taiwan (province of China)	122,309 (107,685 to 139,161)	149.5 (122.6 to 179.6)	6.7 (-4.5 to 19.6)	51,155 (36,505 to 68,072)	11.5 (2.5 to 21.0)	-12.4 (-19.6 to -4.9)
**Southeast Asia**	4,022,869 (3,660,623 to 4,399,803)	44.6 (20.4 to 68.8)	-17.5 (-28.0 to -5.2)	3,731,148 (3,090,111 to 4,498,360)	10.0 (-4.3 to 26.9)	-31.9 (-42.7 to -19.6)
Cambodia	96,483 (79,440 to 116,284)	13.4 (-35.1 to 101.8)	-18.6 (-44.6 to 26.8)	111,582 (86,118 to 138,273)	-12.3 (-35.4 to 23.6)	-50.2 (-64.0 to -24.5)
Indonesia	1,460,134 (1,231,751 to 1,702,772)	21.4 (-13.1 to 61.3)	-27.4 (-42.9 to -7.8)	1,407,277 (1,123,754 to 1,726,919)	0.1 (-20.8 to 26.2)	-38.7 (-52.8 to -20.9)
Laos	46,405 (36,045 to 63,101)	-17.0 (-54.7 to 56.9)	-27.4 (-52.6 to 20.5)	58,787 (45,366 to 73,199)	-24.1 (-46.1 to 14.0)	-52.6 (-65.9 to -26.7)
Malaysia	138,017 (120,909 to 154,909)	90.8 (67.3 to 117.4)	-22.4 (-33.1 to -10.4)	116,433 (89,595 to 147,538)	43.6 (29.9 to 57.3)	-25.4 (-34.2 to -17.0)
Maldives	1,329 (1,167 to 1,496)	-45.6 (-63.8 to -18.4)	-64.9 (-71.4 to -55.2)	1,202 (872 to 1,596)	-37.3 (-56.7 to -9.3)	-58.9 (-69.0 to -46.0)
Mauritius	6,368 (5,845 to 6,900)	12.5 (4.4 to 22.1)	-45.7 (-49.8 to -41.1)	7,454 (6,075 to 9,028)	-4.9 (-13.1 to 3.9)	-42.4 (-48.9 to -36.1)
Myanmar	515,742 (378,116 to 672,662)	26.6 (-11.4 to 90.0)	-18.1 (-42.7 to 23.8)	443,165 (321,779 to 586,951)	-5.7 (-33.0 to 34.7)	-35.8 (-56.7 to -2.4)
Philippines	642,348 (591,718 to 695,030)	112.4 (86.4 to 142.1)	20.2 (8.0 to 35.3)	686,634 (551,371 to 848,366)	34.7 (21.1 to 51.1)	-22.7 (-31.4 to -13.7)
Sri Lanka	110,319 (94,230 to 127,932)	83.9 (56.1 to 114.4)	17.8 (-0.3 to 37.6)	167,349 (134,991 to 202,549)	22.8 (6.9 to 40.5)	-14.1 (-25.2 to -2.2)
Seychelles	495 (442 to 546)	21.4 (9.0 to 35.4)	-28.8 (-36.2 to -20.4)	410 (316 to 515)	11.1 (-0.5 to 23.2)	-23.5 (-32.9 to -14.7)
Thailand	474,367 (407,013 to 544,437)	104.7 (73.9 to 137.4)	-2.6 (-17.2 to 12.9)	354,122 (272,918 to 440,945)	45.6 (28.2 to 70.7)	10.0 (-1.1 to 24.0)
Timor-Leste	6,188 (4,567 to 8,766)	-4.5 (-53.5 to 129.9)	-26.4 (-54.1 to 26.7)	7,708 (5,846 to 9,913)	-17.8 (-48.9 to 42.0)	-51.9 (-67.3 to -21.9)
Vietnam	516,950 (430,085 to 603,334)	50.1 (17.3 to 96.6)	-14.9 (-32.9 to 9.6)	363,196 (281,924 to 461,807)	14.9 (-10.1 to 45.7)	-28.3 (-46.1 to -4.5)
**Oceania**	208,506 (148,923 to 288,571)	48.4 (3.4 to 117.0)	-25.4 (-46.4 to 7.6)	157,388 (117,550 to 212,539)	52.8 (14.2 to 105.2)	-23.1 (-43.5 to 6.6)
American Samoa	428 (377 to 489)	16.0 (-1.0 to 36.1)	-36.5 (-45.4 to -26.2)	424 (305 to 567)	67.9 (46.9 to 87.8)	-13.4 (-25.9 to -0.9)
Federated States of Micronesia	730 (529 to 1,090)	-25.7 (-49.2 to 12.8)	-41.5 (-59.7 to -9.3)	666 (480 to 917)	-9.8 (-29.5 to 15.2)	-27.6 (-46.6 to 1.4)
Fiji	4,714 (4,087 to 5,450)	32.9 (8.5 to 62.9)	-24.5 (-37.8 to -7.2)	10,116 (8,346 to 12,128)	15.5 (-2.7 to 35.6)	-27.8 (-40.0 to -13.6)
Guam	1,231 (1,106 to 1,378)	65.0 (44.2 to 87.6)	-18.2 (-28.0 to -7.6)	844 (602 to 1,120)	64.5 (53.6 to 76.2)	8.5 (-0.1 to 15.8)
Kiribati	712 (595 to 861)	6.6 (-21.3 to 41.5)	-33.7 (-46.3 to -16.9)	1,440 (1,169 to 1,732)	24.4 (3.8 to 47.5)	-24.1 (-36.8 to -9.4)
Marshall Islands	502 (405 to 604)	18.8 (-9.0 to 50.5)	-32.9 (-45.4 to -17.4)	462 (345 to 594)	40.9 (17.6 to 65.8)	-18.6 (-35.3 to 0.4)
Northern Mariana Islands	380 (338 to 430)	56.2 (34.1 to 82.8)	-40.0 (-48.0 to -30.3)	405 (281 to 542)	125.7 (101.7 to 148.4)	-21.5 (-32.5 to -11.5)
Papua New Guinea	167,558 (114,428 to 239,800)	51.8 (0.8 to 131.6)	-26.1 (-49.0 to 11.6)	120,758 (86,285 to 167,937)	59.6 (12.6 to 126.7)	-24.7 (-47.6 to 11.3)
Samoa	973 (786 to 1,161)	-25.4 (-41.4 to -6.8)	-49.2 (-60.2 to -36.6)	985 (722 to 1,304)	-2.7 (-19.7 to 14.6)	-31.9 (-45.8 to -15.9)
Solomon Islands	6,215 (4,320 to 9,028)	34.5 (-8.6 to 111.8)	-36.3 (-56.2 to -1.3)	4,632 (3,234 to 6,348)	57.6 (14.1 to 112.6)	-26.2 (-49.4 to 7.6)
Tonga	725 (626 to 845)	-6.8 (-22.5 to 13.2)	-30.7 (-42.7 to -15.8)	717 (511 to 931)	13.9 (-0.8 to 28.7)	-10.6 (-25.1 to 3.3)
Vanuatu	2,713 (2,006 to 3,753)	3.6 (-33.3 to 66.7)	-50.1 (-67.4 to -21.4)	2,088 (1,499 to 2,805)	79.6 (28.8 to 152.6)	-9.3 (-39.9 to 37.4)
**North Africa and Middle East**	1,885,767 (1,726,137 to 2,047,890)	0.6 (-11.4 to 17.5)	-40.9 (-45.9 to -34.0)	2,132,523 (1,619,890 to 2,748,566)	15.1 (2.9 to 27.9)	-32.2 (-40.6 to -23.7)
**North Africa and Middle East**	1,885,767 (1,726,137 to 2,047,890)	0.6 (-11.4 to 17.5)	-40.9 (-45.9 to -34.0)	2,132,523 (1,619,890 to 2,748,566)	15.1 (2.9 to 27.9)	-32.2 (-40.6 to -23.7)
Afghanistan	225,295 (163,556 to 293,197)	176.5 (90.8 to 327.0)	8.8 (-23.6 to 66.6)	266,353 (200,434 to 339,266)	97.8 (48.2 to 173.2)	-30.6 (-49.9 to 1.0)
Algeria	60,856 (53,739 to 68,798)	40.4 (14.9 to 69.0)	-33.8 (-44.4 to -21.1)	133,923 (96,426 to 177,272)	37.3 (23.6 to 50.4)	-20.5 (-30.4 to -11.3)

	COPD			Asthma		
	DALYs	Change in DALY numbers from 1990 to 2015 (percent)	Change in DALYs from 1990 to 2015, age-standardised rates (percent)	DALYs	Change in DALY numbers from 1990 to 2015 (percent)	Change in DALYs from 1990 to 2015, age-standardised rates (percent)
Bahrain	1,985 (1,712 to 2,257)	19.9 (0.3 to 43.3)	-57.5 (-64.2 to -50.0)	3,486 (2,416 to 4,751)	105.9 (81.6 to 129.1)	-32.2 (-41.2 to -24.1)
Egypt	329,370 (305,686 to 358,820)	-45.2 (-54.7 to -19.0)	-56.1 (-61.1 to -44.3)	282,077 (200,919 to 380,319)	0.9 (-14.3 to 16.0)	-32.7 (-42.3 to -22.3)
Iran	253,619 (212,532 to 297,629)	27.1 (-8.3 to 72.7)	-16.6 (-34.5 to 4.4)	274,162 (206,284 to 348,408)	0.3 (-14.6 to 16.8)	-34.5 (-44.5 to -22.8)
Iraq	47,418 (36,502 to 59,305)	83.0 (30.7 to 148.3)	-5.6 (-34.2 to 29.0)	113,995 (80,893 to 153,424)	79.8 (56.0 to 103.6)	-12.4 (-27.0 to 3.1)
Jordan	9,134 (7,908 to 10,392)	17.5 (-4.3 to 42.2)	-54.2 (-63.4 to -43.7)	25,171 (17,240 to 34,428)	84.7 (64.5 to 104.5)	-22.7 (-33.5 to -13.7)
Kuwait	2,573 (2,239 to 2,980)	105.6 (79.9 to 132.8)	-7.1 (-19.6 to 6.6)	10,609 (6,987 to 14,644)	43.1 (29.3 to 57.1)	-22.7 (-30.8 to -15.7)
Lebanon	14,415 (11,401 to 17,515)	33.6 (2.9 to 68.1)	-49.0 (-61.2 to -34.7)	22,565 (15,705 to 30,483)	54.2 (32.7 to 74.8)	-30.3 (-41.6 to -20.0)
Libya	16,271 (14,066 to 18,660)	40.1 (13.6 to 69.5)	-20.6 (-35.3 to -4.2)	21,246 (15,296 to 28,070)	12.8 (-3.7 to 28.5)	-21.2 (-31.8 to -10.7)
Morocco	78,972 (64,508 to 99,207)	61.4 (16.7 to 125.9)	-11.6 (-36.7 to 24.0)	144,468 (111,241 to 184,491)	0.2 (-17.3 to 23.2)	-38.7 (-51.6 to -22.6)
Palestine	7,318 (6,032 to 8,790)	103.8 (56.9 to 163.9)	-18.7 (-39.5 to 6.7)	14,664 (10,275 to 19,943)	105.6 (83.7 to 127.6)	-13.3 (-26.7 to -0.8)
Oman	5,473 (4,705 to 6,277)	107.5 (58.7 to 160.2)	-13.1 (-32.3 to 10.1)	10,221 (6,983 to 13,965)	96.2 (74.6 to 122.1)	-5.5 (-15.6 to 5.6)
Qatar	1,787 (1,505 to 2,119)	190.1 (142.8 to 238.9)	-34.8 (-46.8 to -21.0)	3,653 (2,411 to 5,017)	291.6 (251.6 to 330.6)	-4.6 (-12.6 to 3.5)
Saudi Arabia	31,046 (27,857 to 34,451)	79.3 (60.3 to 102.3)	-24.4 (-33.2 to -13.5)	82,921 (57,907 to 111,862)	48.5 (35.3 to 61.3)	-20.9 (-28.4 to -14.4)
Sudan	190,964 (134,885 to 263,128)	67.8 (13.0 to 146.3)	-9.9 (-37.9 to 28.4)	212,943 (151,134 to 286,632)	7.2 (-21.8 to 44.0)	-47.6 (-61.1 to -27.6)
Syria	28,085 (24,206 to 32,390)	-28.1 (-52.3 to 8.5)	-47.7 (-59.7 to -31.9)	90,274 (69,943 to 115,584)	-18.0 (-34.4 to 1.7)	-49.2 (-57.9 to -38.9)
Tunisia	37,914 (30,778 to 46,102)	21.2 (-5.5 to 54.8)	-40.4 (-53.8 to -23.0)	43,397 (32,011 to 56,453)	-0.8 (-15.5 to 13.8)	-34.1 (-45.0 to -23.9)
Turkey	388,003 (355,673 to 426,469)	-27.2 (-42.7 to -11.0)	-57.2 (-64.0 to -49.4)	213,948 (151,528 to 286,639)	-19.7 (-34.9 to -2.6)	-41.1 (-51.6 to -29.7)
United Arab Emirates	34,767 (25,331 to 46,621)	297.2 (166.0 to 471.8)	-38.6 (-55.6 to -12.2)	32,805 (23,888 to 42,919)	252.0 (179.4 to 335.4)	-36.3 (-48.1 to -23.2)
Yemen	118,744 (74,827 to 181,921)	29.6 (-23.7 to 124.3)	-29.6 (-58.9 to 26.2)	127,564 (90,238 to 176,823)	20.5 (-17.1 to 70.6)	-43.3 (-62.4 to -16.8)
**South Asia**	26,960,854 (25,334,541 to 28,511,629)	35.4 (15.8 to 59.5)	-29.0 (-39.4 to -16.5)	7,453,917 (6,049,252 to 8,967,827)	-35.3 (-50.9 to -1.3)	-64.3 (-74.5 to -38.3)
**South Asia**	26,960,854 (25,334,541 to 28,511,629)	35.4 (15.8 to 59.5)	-29.0 (-39.4 to -16.5)	7,453,917 (6,049,252 to 8,967,827)	-35.3 (-50.9 to -1.3)	-64.3 (-74.5 to -38.3)
Bangladesh	1,579,294 (1,374,227 to 1,829,318)	20.4 (-11.2 to 74.0)	-32.1 (-49.8 to -4.4)	796,233 (612,113 to 993,582)	-39.7 (-55.6 to -5.0)	-65.6 (-75.7 to -38.0)
Bhutan	7,469 (6,030 to 9,041)	-10.3 (-36.0 to 25.9)	-44.9 (-58.1 to -28.2)	3,002 (2,187 to 3,891)	-31.7 (-54.9 to -1.5)	-50.8 (-65.8 to -30.7)
India	23,363,904 (21,832,725 to 24,891,843)	34.0 (14.9 to 57.4)	-30.4 (-40.1 to -18.6)	5,635,384 (4,596,138 to 6,759,944)	-39.4 (-55.0 to -5.4)	-66.6 (-76.4 to -41.4)
Nepal	373,300 (302,597 to 458,721)	48.0 (2.2 to 121.0)	-14.2 (-39.6 to 24.8)	169,932 (124,218 to 220,914)	-21.4 (-47.9 to 28.5)	-55.0 (-72.5 to -17.2)
Pakistan	1,636,887 (1,407,817 to 1,921,491)	81.1 (33.7 to 140.7)	1.7 (-19.0 to 28.4)	849,365 (639,064 to 1,097,087)	24.9 (-5.3 to 61.8)	-31.1 (-48.8 to -5.6)
**Sub-Saharan Africa**	2,897,718 (2,599,236 to 3,228,864)	41.1 (23.9 to 61.9)	-25.4 (-34.2 to -14.4)	4,714,673 (3,699,646 to 5,914,317)	37.4 (21.6 to 53.1)	-32.2 (-40.3 to -22.6)
**Southern sub-Saharan Africa**	508,747 (465,366 to 558,398)	35.9 (23.3 to 51.4)	-22.7 (-30.0 to -13.4)	485,667 (387,533 to 606,688)	11.6 (2.9 to 22.3)	-25.3 (-32.7 to -16.4)
Botswana	14,741 (7,686 to 35,910)	72.6 (-29.4 to 358.8)	-22.9 (-69.0 to 103.0)	16,216 (9,400 to 34,076)	45.6 (-20.3 to 194.5)	-17.7 (-61.5 to 90.7)
Lesotho	21,255 (13,574 to 30,268)	74.0 (8.4 to 171.4)	31.9 (-18.9 to 107.8)	22,485 (15,520 to 31,248)	48.1 (3.7 to 113.4)	17.3 (-25.5 to 81.8)
Namibia	12,112 (8,382 to 17,474)	23.4 (-15.1 to 80.9)	-35.4 (-56.4 to -4.8)	15,008 (10,617 to 20,204)	22.1 (-1.0 to 52.2)	-35.2 (-51.1 to -14.1)
South Africa	397,475 (366,246 to 430,305)	31.5 (19.3 to 44.9)	-27.0 (-33.9 to -19.2)	331,109 (263,984 to 415,264)	0.0 (-8.2 to 8.3)	-31.9 (-38.8 to -24.6)
Swaziland	10,535 (6,259 to 15,828)	69.4 (-4.0 to 171.6)	-6.8 (-48.0 to 52.0)	12,297 (8,358 to 17,309)	54.4 (13.8 to 114.1)	3.0 (-33.7 to 59.2)
Zimbabwe	52,629 (37,837 to 72,051)	49.2 (3.5 to 108.7)	1.8 (-32.2 to 46.2)	88,552 (63,561 to 117,156)	54.7 (26.8 to 95.7)	7.6 (-20.9 to 49.4)
**Western sub-Saharan Africa**	798,640 (682,146 to 932,871)	30.3 (3.9 to 63.3)	-32.2 (-45.9 to -14.5)	1,565,132 (1,212,360 to 1,989,966)	39.6 (17.1 to 62.2)	-34.4 (-46.9 to -20.7)
Benin	32,509 (19,832 to 51,175)	80.0 (8.0 to 192.8)	-15.5 (-50.2 to 36.7)	49,682 (34,641 to 68,997)	82.4 (33.2 to 155.4)	-16.6 (-44.0 to 25.0)
Burkina Faso	33,218 (23,171 to 47,644)	47.9 (-5.1 to 132.5)	-14.1 (-45.5 to 33.2)	65,919 (47,374 to 87,290)	54.3 (16.4 to 98.6)	-24.5 (-46.1 to 8.3)
Cameroon	62,337 (40,458 to 94,880)	40.4 (-11.4 to 122.2)	-25.4 (-54.1 to 17.4)	101,907 (70,234 to 137,886)	55.3 (19.4 to 101.2)	-23.5 (-46.0 to 7.3)

	COPD			Asthma		
	DALYs	Change in DALY numbers from 1990 to 2015 (percent)	Change in DALYs from 1990 to 2015, age-standardised rates (percent)	DALYs	Change in DALY numbers from 1990 to 2015 (percent)	Change in DALYs from 1990 to 2015, age-standardised rates (percent)
Cape Verde	1,141 (967 to 1,367)	-38.2 (-50.6 to -24.0)	-64.8 (-71.9 to -55.9)	1,677 (1,271 to 2,134)	-20.3 (-33.8 to -7.4)	-55.9 (-64.3 to -47.2)
Chad	31,722 (19,383 to 48,915)	119.6 (21.1 to 292.9)	3.0 (-44.4 to 92.8)	71,498 (48,132 to 101,314)	100.5 (40.0 to 176.2)	-21.1 (-51.1 to 22.5)
Cote d'Ivoire	71,504 (46,681 to 106,802)	37.1 (-12.3 to 116.5)	-29.6 (-55.6 to 11.1)	111,043 (80,312 to 150,421)	41.1 (7.9 to 83.4)	-29.0 (-49.2 to -1.0)
The Gambia	3,579 (2,615 to 4,765)	16.4 (-31.1 to 104.8)	-42.5 (-66.3 to 3.6)	6,923 (4,959 to 9,065)	68.3 (22.4 to 125.5)	-25.5 (-50.8 to 13.4)
Ghana	62,477 (39,896 to 94,740)	60.8 (-7.3 to 185.8)	-24.1 (-56.4 to 35.3)	91,376 (63,211 to 126,002)	51.6 (9.1 to 119.7)	-23.7 (-49.5 to 20.7)
Guinea	41,905 (28,575 to 59,946)	89.6 (25.2 to 193.2)	-3.3 (-37.4 to 48.6)	66,418 (48,077 to 88,823)	62.5 (26.1 to 107.4)	-22.7 (-44.3 to 4.8)
Guinea-Bissau	7,475 (3,458 to 18,030)	22.8 (-57.2 to 247.0)	-28.4 (-76.5 to 105.2)	11,565 (6,584 to 23,279)	57.4 (-22.4 to 240.8)	-13.4 (-63.1 to 118.1)
Liberia	8,507 (6,146 to 11,934)	45.1 (-9.5 to 130.9)	-16.2 (-44.2 to 25.2)	15,571 (11,445 to 20,488)	51.7 (17.6 to 95.6)	-20.8 (-41.5 to 7.7)
Mali	53,406 (36,219 to 79,111)	26.0 (-19.1 to 99.0)	-21.9 (-49.1 to 21.0)	85,302 (60,223 to 117,292)	32.2 (-9.5 to 93.0)	-32.2 (-56.0 to 5.5)
Mauritania	6,653 (4,555 to 9,490)	13.2 (-28.9 to 80.5)	-48.7 (-67.7 to -16.5)	16,228 (11,619 to 21,723)	26.5 (-3.0 to 59.7)	-48.4 (-63.6 to -28.5)
Niger	43,146 (29,753 to 60,987)	91.4 (14.7 to 238.3)	-21.9 (-52.6 to 40.8)	86,380 (62,640 to 114,059)	68.5 (25.4 to 127.3)	-36.7 (-57.9 to -4.1)
Nigeria	262,068 (187,195 to 366,732)	1.0 (-33.7 to 60.8)	-47.1 (-65.2 to -15.6)	655,915 (462,331 to 894,035)	24.3 (-7.3 to 61.7)	-43.5 (-60.3 to -20.4)
Sao Tome and Principe	1,318 (805 to 1,988)	28.2 (-19.2 to 93.3)	-1.9 (-40.6 to 48.5)	1,075 (754 to 1,476)	29.0 (-5.3 to 75.5)	-9.7 (-38.7 to 32.2)
Senegal	36,101 (22,903 to 55,877)	50.7 (-8.6 to 142.1)	-23.5 (-54.0 to 21.5)	59,656 (41,284 to 83,959)	40.7 (2.7 to 95.4)	-32.0 (-55.1 to 2.1)
Sierra Leone	19,830 (13,185 to 28,656)	32.6 (-17.3 to 117.0)	-10.9 (-45.8 to 47.2)	31,863 (22,904 to 42,384)	37.2 (-1.7 to 88.9)	-14.4 (-43.2 to 28.3)
Togo	19,721 (13,259 to 27,656)	47.6 (-4.8 to 121.8)	-24.2 (-50.7 to 15.8)	35,112 (25,534 to 46,564)	50.7 (18.6 to 89.3)	-26.2 (-45.7 to 1.5)
**Eastern sub-Saharan Africa**	1,065,419 (906,695 to 1,250,429)	38.4 (16.6 to 66.3)	-25.9 (-38.3 to -11.6)	1,793,733 (1,391,074 to 2,264,531)	30.5 (12.7 to 49.4)	-37.4 (-47.5 to -25.8)
Burundi	38,509 (26,184 to 55,926)	-17.2 (-47.7 to 33.5)	-53.6 (-71.9 to -23.6)	60,533 (43,650 to 81,809)	-6.3 (-34.2 to 32.7)	-56.0 (-71.4 to -31.4)
Comoros	1,980 (1,421 to 2,791)	-5.0 (-41.7 to 53.0)	-43.8 (-64.7 to -10.6)	3,588 (2,551 to 4,877)	9.4 (-19.2 to 42.9)	-43.6 (-61.8 to -20.9)
Djibouti	2,808 (1,687 to 4,650)	41.3 (-23.3 to 154.9)	-19.4 (-55.5 to 48.3)	4,406 (2,967 to 6,304)	21.0 (-11.8 to 73.4)	-19.6 (-45.3 to 23.7)
Eritrea	16,766 (10,144 to 26,389)	23.0 (-31.3 to 116.9)	-29.2 (-60.8 to 24.0)	28,613 (19,444 to 40,949)	10.4 (-24.3 to 63.2)	-35.9 (-60.6 to 5.4)
Ethiopia	233,383 (158,142 to 347,698)	12.3 (-27.0 to 74.0)	-42.4 (-62.9 to -9.3)	410,688 (292,188 to 553,900)	2.2 (-25.1 to 38.1)	-54.9 (-68.4 to -35.6)
Kenya	98,293 (85,248 to 113,027)	88.3 (64.8 to 113.8)	-8.1 (-20.3 to 4.9)	157,871 (118,272 to 203,919)	57.0 (44.8 to 69.3)	-20.7 (-29.6 to -11.9)
Madagascar	135,109 (91,132 to 191,435)	48.1 (-2.8 to 124.5)	-15.8 (-43.8 to 22.3)	212,223 (149,005 to 289,772)	52.4 (12.8 to 107.8)	-20.3 (-44.1 to 12.0)
Malawi	42,111 (30,406 to 60,186)	34.7 (-13.3 to 108.7)	-20.4 (-45.9 to 21.2)	72,522 (51,760 to 96,667)	38.3 (5.1 to 77.6)	-24.0 (-43.1 to 2.4)
Mozambique	78,780 (52,720 to 112,393)	87.2 (7.8 to 199.8)	8.0 (-33.4 to 62.6)	128,816 (91,742 to 174,836)	72.1 (30.7 to 121.9)	-14.6 (-41.8 to 21.3)
Rwanda	36,110 (25,356 to 53,249)	-6.9 (-38.7 to 51.7)	-43.7 (-63.4 to -8.5)	62,410 (44,955 to 84,665)	-17.9 (-38.1 to 7.2)	-54.5 (-66.8 to -34.8)
Somalia	36,279 (18,446 to 68,206)	14.4 (-45.9 to 162.7)	-14.2 (-63.1 to 116.4)	79,036 (46,536 to 144,914)	14.2 (-37.3 to 113.5)	-24.6 (-65.6 to 76.6)
South Sudan	50,065 (25,919 to 101,836)	62.8 (-30.8 to 281.8)	-15.1 (-66.4 to 120.0)	75,006 (44,762 to 131,315)	46.7 (-16.0 to 168.7)	-26.4 (-65.0 to 62.5)
Tanzania	127,306 (87,332 to 190,630)	75.2 (14.4 to 170.7)	-15.0 (-44.2 to 33.3)	227,278 (160,158 to 304,434)	67.0 (34.2 to 108.0)	-21.4 (-42.2 to 7.2)
Uganda	109,027 (73,627 to 153,859)	36.3 (-12.2 to 110.0)	-26.5 (-53.5 to 13.5)	192,132 (135,517 to 255,836)	46.9 (11.1 to 88.0)	-35.4 (-54.8 to -9.1)
Zambia	58,121 (41,698 to 78,419)	118.3 (44.2 to 220.3)	26.5 (-16.7 to 84.5)	77,425 (56,796 to 102,087)	76.1 (40.1 to 121.2)	2.1 (-23.7 to 39.4)
**Central sub-Saharan Africa**	524,912 (393,633 to 693,446)	77.2 (24.0 to 152.9)	-7.8 (-36.7 to 33.4)	870,140 (650,408 to 1,136,882)	74.0 (34.0 to 123.1)	-16.9 (-38.6 to 13.0)
Angola	114,458 (61,923 to 236,761)	43.2 (-35.1 to 244.2)	-26.1 (-70.1 to 100.1)	200,833 (127,228 to 314,309)	41.0 (-16.6 to 136.2)	-35.0 (-67.0 to 36.2)
Central African Republic	37,183 (22,173 to 55,352)	41.3 (-17.8 to 123.5)	-9.0 (-48.8 to 46.0)	53,039 (34,229 to 76,744)	59.1 (8.3 to 129.8)	2.3 (-36.1 to 55.2)
Congo (Brazzaville)	22,433 (15,249 to 32,818)	23.5 (-19.4 to 86.5)	-35.3 (-58.6 to -1.2)	29,602 (20,789 to 40,110)	44.5 (10.2 to 87.1)	-29.1 (-50.0 to -0.9)
Democratic Republic of the Congo	339,561 (235,953 to 464,669)	110.9 (31.0 to 230.6)	7.9 (-33.8 to 77.8)	569,690 (419,370 to 769,783)	95.7 (40.8 to 163.9)	-9.2 (-38.4 to 33.7)
Equatorial Guinea	4,040 (2,301 to 8,771)	17.2 (-45.8 to 227.1)	-41.0 (-73.5 to 70.9)	6,091 (4,004 to 9,516)	29.5 (-25.7 to 138.0)	-48.6 (-72.7 to 11.3)

	COPD			Asthma		
	DALYs	Change in DALY numbers from 1990 to 2015 (percent)	Change in DALYs from 1990 to 2015, age-standardised rates (percent)	DALYs	Change in DALY numbers from 1990 to 2015 (percent)	Change in DALYs from 1990 to 2015, age-standardised rates (percent)
Gabon	7,237 (5,285 to 10,181)	-1.8 (-31.4 to 47.8)	-38.1 (-57.4 to -5.8)	10,886 (7,893 to 14,537)	34.8 (11.3 to 63.9)	-25.1 (-40.1 to -4.3)
